# Reversibly tuning hydrogel stiffness through photocontrolled dynamic covalent crosslinks†
†Electronic supplementary information (ESI) available. See DOI: 10.1039/c8sc02093k


**DOI:** 10.1039/c8sc02093k

**Published:** 2018-06-19

**Authors:** Joseph V. Accardo, Julia A. Kalow

**Affiliations:** a Department of Chemistry , Northwestern University , 2145 Sheridan Rd. , Evanston , IL 60208 , USA . Email: jkalow@northwestern.edu

## Abstract

Controlling the physical properties of soft materials with external stimuli enables researchers to mimic and study dynamic systems. Of particular interest are hydrogels, polymer networks swollen by water with broad applicability to biomedicine. To control hydrogel mechanics with light, researchers have relied on a limited number of photochemical reactions. Here we introduce an approach to reversibly tune hydrogel mechanics with light by manipulating the stability of dynamic covalent crosslinks at the molecular level. The equilibrium between a boronic acid and diol to form a boronic ester can be altered by the configuration of an adjacent azobenzene photoswitch. By irradiating branched polymers bearing azobenzene-boronic acid and diol end groups with two different wavelengths of light, we can stiffen or soften the resulting hydrogel. Alternating irradiation induces reversible mechanical changes. Rheological characterization reveals that the hydrogels are viscoelastic, exhibiting stress relaxation on the order of seconds, and the stiffness is tuned independently of the crossover frequency. We have also demonstrated that this approach can be extended to use visible light for both softening and stiffening. These photocontrolled dynamic covalent crosslinks provide a versatile platform for tunable dynamic materials.

## Abstract 2

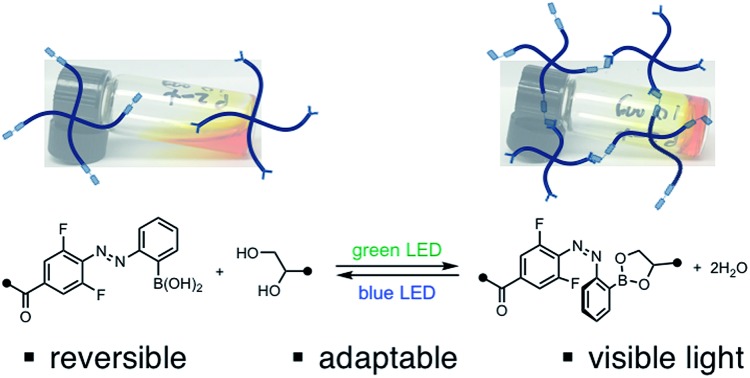
By controlling the stability of dynamic covalent crosslinks with adjacent photoswitches, the stiffness of an adaptable hydrogel is tuned reversibly.

## Introduction

Polymer networks crosslinked with dynamic bonds can be self-healing, adaptive, and recyclable.^1^ The conditions under which these properties are observed depend on the stability and lifetime of the dynamic bonds. By tailoring crosslink stability and reactivity, macroscopic properties can be programmed at the molecular level. Furthermore, if changes in crosslink density or dynamics occur in response to a stimulus, these materials exhibit tunable macroscopic properties. External stimuli such as pH, temperature, and magnetic field have been employed to reversibly tune the properties of polymer networks.^2^

As soft materials with mechanics and water content that approximate those of tissues, hydrogels benefit from the introduction of reversibly, externally controlled properties.^3^ While traditional stimuli such as pH or temperature present limitations on biocompatibility, light (particularly in the visible to near-IR range) represents an ideal stimulus. Light can be applied externally with precise spatial and temporal control, at controlled wavelengths and fluxes. However, the majority of photocontrolled hydrogels rely on irreversible photochemical reactions, such as photoinitiated radical polymerization and exchange, and *o*-nitrobenzyl cleavage.^4^ In addition to their irreversibility, these reactions can suffer from the requirement for exogeneous reagents, generation of byproducts, or sensitivity to oxygen. While many clever designs for reversible photocontrol have been described for organogels and liquid crystal elastomers, the translation of these chemistries to hydrogels may not be feasible.^5^

**Fig. 1:**
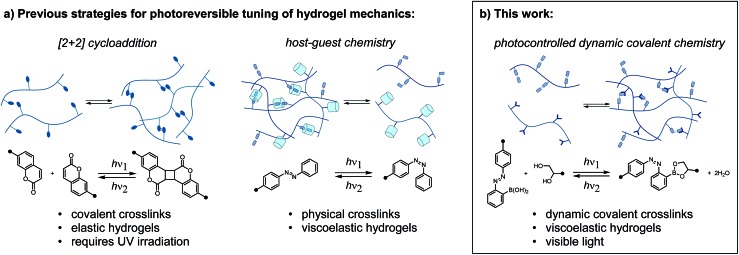
(a) Previous strategies for photoreversible control over hydrogel mechanics rely on photocycloadditions or supramolecular complexes with photoswitch guests. (b) In this work, the configuration of an adjacent photoswitch controls the stability of a dynamic covalent crosslink, thus reversibly tuning mechanics with light.

Reversibly tuning hydrogel mechanics has been challenging due to the limited number of aqueous photoreversible reactions that can be coupled to a change in crosslink density. Covalently linked hydrogels based on photoreversible [2 + 2] cycloadditions display reversible stiffening and softening (Fig. 1a).^6^ While recent work has achieved the cycloaddition with visible light, the reverse reaction invariably requires UV irradiation. As an alternative to photoreversible reactions, many researchers turn to the well-studied photoswitch azobenzene, which undergoes reversible *E*/*Z* isomerization in response to two different wavelengths of light.^7^ Rosales and coworkers enchained azobenzene in an elastic network and observed small but reproducible changes in stiffness.^8^ It is important to note that the above systems are not dynamic or adaptable in the absence of light; these elastic networks store, rather than dissipate, energy from applied strain. To achieve a sol–gel transition in a stress-relaxing network, Harada and coworkers designed a supramolecular hydrogel based on cyclodextrin/azobenzene complexes, which has been leveraged in multiple contexts (Fig. 1a).^9^

Herein, we report a distinct approach to reversibly tune hydrogel mechanics by photocontrolling the stability of crosslinks at the molecular level (Fig. 1b). This strategy benefits from the strength and directionality of dynamic covalent bonds, while taking advantage of an azobenzene photoswitch for external control. Because we use different functional groups for crosslinking and photoexcitation, we can readily modulate the photophysics of the system without compromising reactivity.

Our photoresponsive hydrogels rely on boronic ester crosslinks, which undergo reversible exchange by hydrolysis and esterification. Researchers have extensively employed the boronic ester crosslink in sugar-responsive hydrogels and in 3D cell culture.^10^ Work by Kawashima and coworkers has demonstrated that the *E*/*Z* isomerization of an azo group can reversibly influence the Lewis acidity of catecholboranes.^11^ We envisioned that an azo group could be used to influence the kinetics or equilibria for interconversion between boronic acids and boronic esters, which would then translate to a photoswitchable change in network mechanics.^12^,^13^ For example, Hecht and coworkers reported that self-healing could be photoswitched on and off in polysiloxane networks with photoresponsive spiropyran-imine crosslinks, wherein the configuration of a spiropyran photoswitch controls the rate of imine exchange.^14^ Our design is the first to explore this concept for boronic ester crosslinks and in hydrogel networks, and the resulting materials can be tuned exclusively with visible light.

## Results and discussion

### Small-molecule model system

**Scheme 1:**
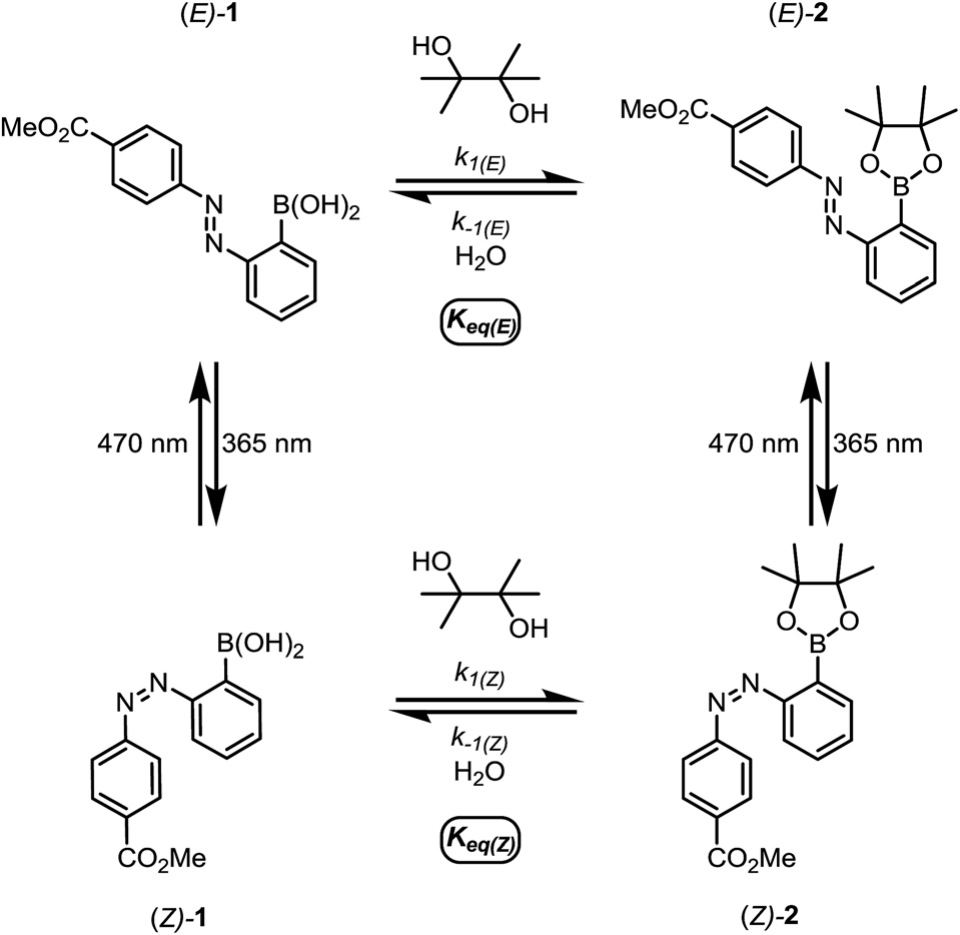
The small-molecule model system for studying the relative rates and equilibrium constants for reversible esterification of an *o*-azobenzene boronic acid.

**Fig. 2:**
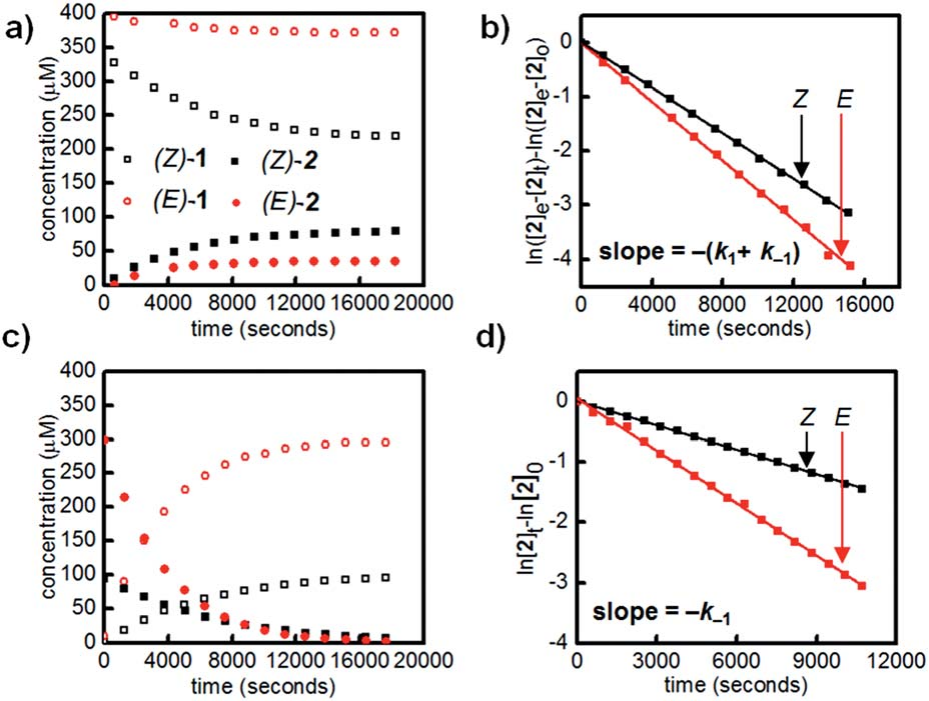
(a) Esterification of (*E*)-**1** (hollow red circles) and (*Z*)-**1** (hollow black squares) with 40 mM of pinacol in 1 : 1 ACN–H_2_O. (b) Linear fit of data from (a) to determine apparent rates of esterification and hydrolysis of *E* and *Z* isomers. (c) Hydrolysis of 400 μM mixture of (*E*)-**2** and (*Z*)-**2** in 1 : 1 ACN : H_2_O. (d) Linear fit of data from (c) to confirm apparent rates of hydrolysis for *E* and *Z* isomers. All experiments were performed at 25 °C.

We first designed a small-molecule model compound, azobenzene **1**, in which the boronic acid is positioned *ortho* to the azo group. To evaluate whether the azobenzene conformation affects the reactivity of the boronic acid, we measured the rates of esterification for *E* and *Z* isomers of **1** to form pinacol esters **2**, as well as the rates of hydrolysis for both isomers of **2** (Scheme 1). The more thermally stable azobenzene isomer, (*E*)-**1** (400 μM), was subjected to excess pinacol (40 mM) in acetonitrile–water (1 : 1 v/v, 25 °C). Consumption of boronic acid (*E*)-**1** and formation of the pinacol ester (*E*)-**2** were followed by high-performance liquid chromatography (HPLC, Fig. 2a, red circles). While other 1,2- and 1,3-diols reacted too quickly to measure rates accurately, the slow rate of pinacol esterification and hydrolysis^15^ allowed us to resolve the *E* and *Z* isomers and to monitor their reaction kinetics by HPLC (see ESI† for details).

Irradiation of a solution of (*E*)-**1** (400 μM) with 365 nm light (10 minutes, 3.6 mW cm^–2^) provided an 88 : 12 mixture of *Z* and *E* isomers. The half-lives of (*Z*)-**1** and (*Z*)-**2** were determined to be at least 21 hours at 25 °C based on an Arrhenius plot (Fig. S10 and S11†). Again, this *E*/*Z* mixture was subjected to excess pinacol in acetonitrile–water, and the consumption of boronic acid (*Z*)-**1** and formation of pinacol ester (*Z*)-**2** were followed by LCMS (Fig. 2a, black squares).

Promisingly, these initial experiments revealed a difference in the reactivity of the *E* and *Z* isomers. After 8 hours, the reactions had reached equilibrium, with 39% conversion of (*Z*)-**1** to (*Z*)-**2** and only 9% conversion of (*E*)-**1** to (*E*)-**2** (Fig. 2a). The reactions were performed with a large excess of both pinacol and water, so a pseudo-first-order approximation can be applied: we assume that the concentrations of pinacol and water are essentially constant throughout the reaction and between isomers. Thus, the data in Table 1 are apparent rate and equilibrium constants (see ESI† for derivations).

**Table 1:** Apparent rate and equilibrium constants for the small-molecule model study. Data are the average of three experiments performed at 25 °C

Configuration	*K*_eq_ ^*a*^	*k*_1_ ^*a*^ (s^–1^)	*k*_–1_ ^*a*^ (s^–1^)	*k*_–1_ ^*b*^ (s^–1^)
*E*	0.090 ± 0.016	2.56 ± 0.28 × 10^–5^	2.76 ± 0.32 × 10^–4^	2.83 ± 0.06 × 10^–4^
*Z*	0.39 ± 0.024	5.39 ± 0.45 × 10^–5^	1.39 ± 0.20 × 10^–4^	1.45 ± 0.10 × 10^–4^
*Z*/*E*	4.3	2.11	0.504	0.512

^*a*^Apparent equilibrium constants and rate constants obtained from the reversible esterification experiment.

^*b*^Apparent rate constants obtained from the irreversible hydrolysis experiments.

Using a reversible pseudo-first-order kinetic model, we determined that the esterification of (*Z*)-**1** (*k*_1(*Z*)_) is 2.1 times faster than the esterification of (*E*)-**1** (*k*_1(*E*)_) (Fig. 2b, Table 1). We could also extract the rates of hydrolysis from this model: (*E*)-**2** undergoes hydrolysis (*k*_–1(*E*)_) 2.0 times faster than (*Z*)-**2** does (*k*_–1(*Z*)_). These apparent rate constants for hydrolysis were verified by hydrolyzing a mixture of (*E*)-**2** and (*Z*)-**2** under irreversible pseudo-first-order conditions (Fig. 2c and d).

Taken together, the apparent equilibrium to form boronic ester from boronic acid and pinacol is 4.3 times more favorable for the *Z* isomer (*K*_eq(*Z*)_) relative to the *E* isomer (*K*_eq(*E*)_). While convenient for small-molecule kinetic studies, the rate of pinacol condensation with boronic acids is too slow to be practical for gelation. Thus, we used a less sterically hindered, previously reported diol for hydrogel studies.

### Hydrogel synthesis and rheological characterization

**Fig. 3:**
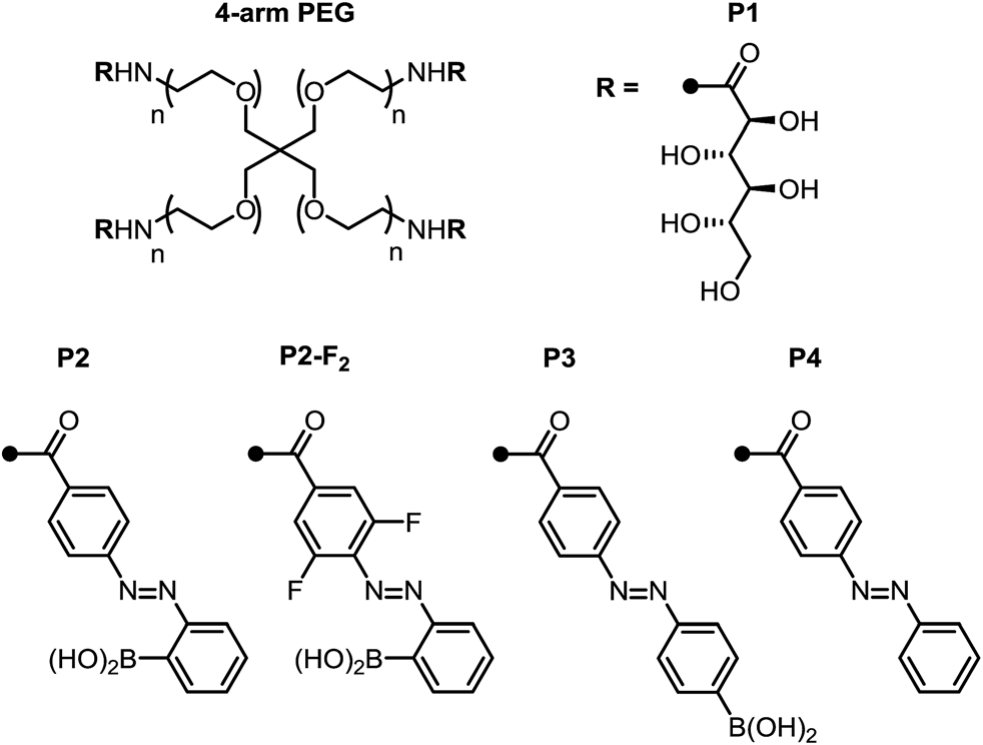
Structure of azobenzene- and diol-terminated poly(ethylene glycol) polymers **P1–P4**.

With these promising small-molecule data in hand, we sought to translate our molecular design to photoswitchable networks. We prepared a pair of branched polymers, **P1** and **P2**, with complementary diol and boronic acid end groups (Fig. 3). The diol-terminated polymer (**P1**) was synthesized by ring opening glucono-δ-lactone with amine-terminated 4-arm poly(ethylene glycol) (PEG, *M*_w_ = 5 kDa) according to a literature procedure.10d,e The boronic acid polymer (**P2**) was synthesized by coupling the same PEG-amine with the carboxylic acid derivative of compound **1** using carbodiimide coupling chemistry (see ESI† for details). Control polymers **P3** and **P4** were synthesized in analogy to **P2** to evaluate the role of the *ortho*-boronic acid.

**Fig. 4:**
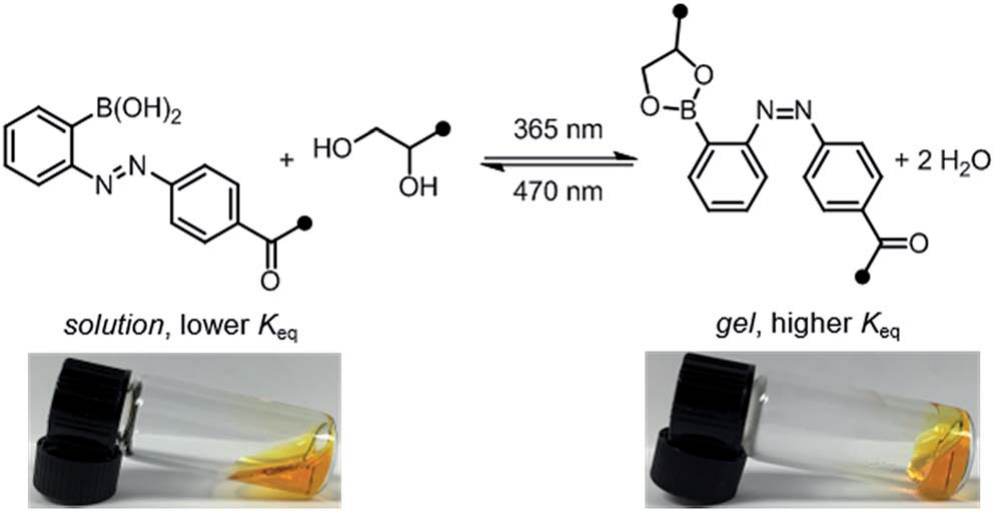
Reversible sol–gel transition of a mixture of **P1** and **P2** (1 : 1, 10 w/v% in PBS, pH 7.5). Gelation was performed by irradiation with 365 nm light (10 min, 3.6 mW cm^–2^); gelation was reversed by irradiation with 470 nm light (30 s, 900 lux).

To qualitatively investigate the effect of irradiation on the boronic ester hydrogel, **P1** and **P2** in 0.1 M phosphate-buffered saline (PBS) at pH 7.5 (10 w/v%) were mixed in a 1 : 1 ratio. Prior to irradiation, the mixture was a sol, according to the flow-inversion method. Irradiation with a 365 nm flashlight (3.6 mW cm^–2^) for 10 minutes induces partial *E* to *Z* isomerization of the azobenzene photoswitch and leads to gelation. Irradiating this gel for 30 seconds with blue LEDs (470 nm, 900 lux) promotes *Z* to *E* isomerization, and returns the mixture to the sol state. The sol–gel cycles could be repeated multiple times by sequential irradiation with 365 and 470 nm light (Fig. 4, ESI Video†).

In contrast, the combination of **P1** and control polymer **P3** (a *para*-boronic acid) form a gel without irradiation, and this gel is not photoresponsive. This observation suggests that proximity to the azo group, rather than an inductive/resonance or rigidity effect, is responsible for the photoresponse.^8^ The combination of **P1** and **P4**, lacking a boronic acid, forms a sol regardless of irradiation, providing evidence that the boronic ester is the crosslink (see ESI† for photographs and rheological characterization of the control gels).

**Fig. 5:**
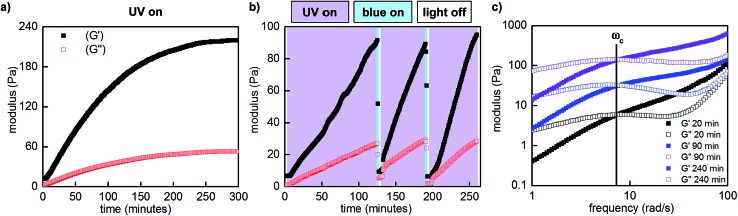
Representative photorheological characterization of the hydrogels obtained at 25–28 °C. (a) UV-induced gelation profile of **P1** and **P2** (1 : 1, 10 w/v% in PBS, pH 7.5, 10% strain, 25 rad s^–1^). (b) Photocontrolled cycling of hydrogel viscoelasticity (10% strain, 25 rad s^–1^). UV light induces gelation, which is reversed with blue light. UV light is required to re-initiate stiffening of the gel. The first gelation cycle is slower than subsequent cycles; for a discussion, see the ESI.† (c) Dynamic frequency sweep measurements as a function of irradiation (10% strain).

To quantitatively assess the photoresponsive bulk mechanical properties of the hydrogel, we performed photo-oscillatory rheology at constant strain and frequency within the linear viscoelastic regime (Fig. S18†). Upon constant irradiation of **P1** and **P2** (1 : 1, 10 w/v% in PBS, pH 7.5) with 365 nm LED light, the storage (*G*′) and loss moduli (*G*′′), which represent the elastic and viscous characteristics of the hydrogel, increased by over an order of magnitude. The maximum storage modulus (220 Pa) was achieved after approximately 5 hours of irradiation (Fig. 5a). Importantly, we could quantitatively demonstrate that this change in mechanical properties is reversible by performing photorheology with alternating 365 and 470 nm light (Fig. 5b). After stiffening the gel with 365 nm light for 2 hours (violet shading), irradiation with 470 nm light for 2 minutes returns the network to its original state (blue shading). Gelation can be repeated by irradiation with 365 nm light. In contrast to strategies based on photocleavage or photoinitiated polymerization, water is the only byproduct and required exogenous reagent.

Unlike literature examples of reversibly controlled hydrogels based on azobenzene photoswitching, this system stiffens in the *Z* conformation and softens in the *E* conformation.^8^,9c We cannot directly correlate our measured rate and equilibrium constants in the small-molecule model system (Table 1) to the viscoelastic behavior of the **P1**/**P2** hydrogel because **P1** bears less sterically hindered diols. Nevertheless, in analogy to the small-molecule model system, we hypothesize that the *Z* azobenzene boronic acid experiences more favorable equilibrium towards the boronic ester compared to the *E* isomer. Since the boronic ester is the elastically effective crosslink, a higher equilibrium constant corresponds to higher crosslink density and thus a stiffer gel.

We next characterized the viscoelastic properties of our hydrogel system as a function of irradiation. Networks formed from static covalent bonds are elastic, and exhibit frequency-independent moduli because the crosslinks are fixed. Dynamically crosslinked networks have time-dependent properties. At higher frequencies, the oscillation occurs faster than the network can rearrange, thus energy is stored elastically and the material behaves as a gel. At lower frequencies, mechanisms to dissipate energy by crosslink rupture or exchange can occur on the time scale of oscillation, and the material behaves as a liquid. The crossover frequency (*ω*_c_) at which *G*′ and *G*′′ are equal corresponds to the oscillation frequency at which the viscoelastic material transitions from more solid-like to more liquid-like.

We performed frequency sweeps on mixtures of **P1** and **P2** (1 : 1, 10 w/v% in PBS, pH 7.5) and observed frequency-dependent viscoelastic behaviour. Consistent with our measurements at constant frequency, when we performed these measurements after various intervals of UV irradiation (20–240 minutes, Fig. 5c), both storage and loss moduli increased. Curiously, the crossover frequency at 8 rad s^–1^ was independent of irradiation time and stiffness. In accordance with these oscillatory data, the gels relax strain-induced stress on the order of seconds, and the rate of stress relaxation is constant as a function of irradiation and stiffening (Fig. S16†).

The crossover frequency of a dynamic frequency sweep measurement is often correlated to the molecular processes underlying crosslink rupture.^13^ In this case, we assign the stress-relaxing process to be hydrolysis of the boronic ester. Our rheological data demonstrate that through photocontrolled dynamic covalent crosslinks, an external stimulus can reversibly alter the spatial structure of a viscoelastic network (crosslink density) without significantly altering the temporal hierarchy (relaxation modes).^16^ For this particular boronic acid/diol combination, we conclude that the change in equilibrium constants for *E versus Z* azobenzene boronic acid, rather than changes in hydrolysis rates, underlies the phototunable change in mechanics. We anticipate that strategic modifications of the boronic acid and diol structures could additionally enable tuning of relaxation modes. The ability to independently tune the spatial and temporal hierarchy of a polymer network represents an important step towards molecularly engineered dynamic materials.

### Visible-light photoswitching

**Fig. 6:**
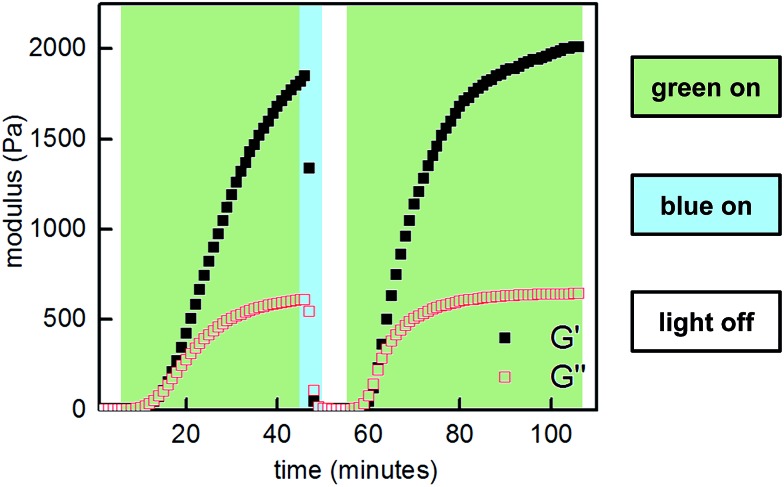
Photocontrolled cycling of hydrogel viscoelasticity (10% strain, 25 rad s^–1^) of **P1** and **P2-F_2_** (1 : 1, 10 w/v% in PBS, pH 7.5). Green light induces gelation, and blue light induces softening.

Next, we sought to optimize our system such that viscoelasticity could be controlled with visible light. In addition to lower energy, which minimizes side reactions (see ESI†), visible light offers enhanced hydrogel penetration. Hecht and coworkers have demonstrated that incorporation of *ortho*-fluorine atoms in azobenzenes leads to visible-light photoswitches with long thermal half-lives for the *Z* isomers.^17^ Inspired by this work, we synthesized polymer **P2-F_2_** (Fig. 3). Hydrogels prepared from mixtures of **P1** and **P2-F_2_** (1 : 1, 10 w/v% in PBS, pH 7.5) demonstrated reversible sol to gel transitions by alternating irradiation with green (525 nm) and blue (470 nm) LEDs. Rheological characterization confirmed that the stiffness of the gels can be reversibly controlled, and the hydrogels are viscoelastic and stress-relaxing (Fig. 6, S21 and S22†). Importantly, gels synthesized from **P2-F_2_** stiffened faster and exhibited maximum moduli that were an order of magnitude larger than those formed from **P2**, which may be due to a higher binding affinity of the electron-deficient difluoroazobenzene boronic acid with diols.

**Fig. 7:**
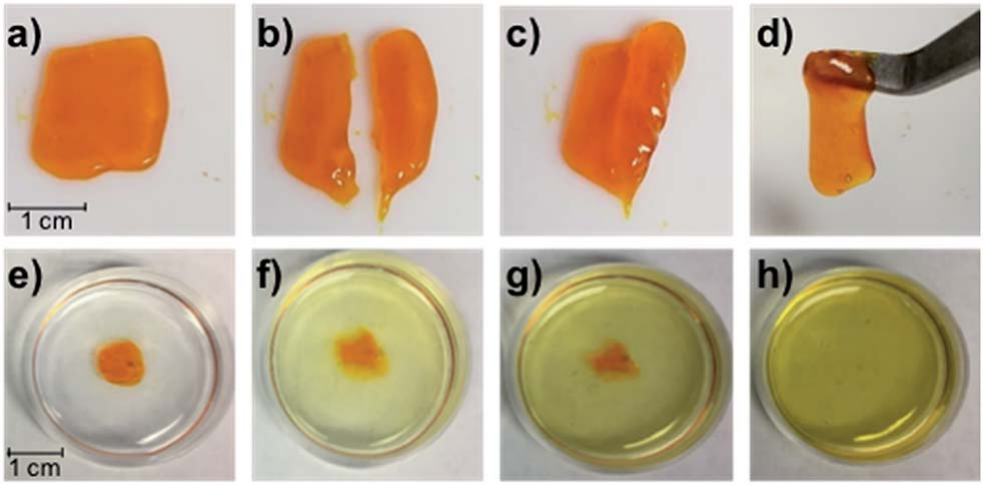
(a–d) Photographs of the self-healing process for the **P1**/**P2-F_2_** gel (1 : 1, 10 w/v% in PBS, pH 7.5). A gel formed after 1 h irradiation with green LEDs (a) can be cut (b) and re-joined ((c) after 5 s; (d) after 60 s). (e–h) Photographs of the swollen gel in PBS at 25 °C after swelling for (e) 0 h, (f) 1 h, (g) 2 h, and (h) 6 h.

Gratifyingly, the **P1**/**P2-F_2_** gel (10 w/v% in PBS, pH 7.5) is sufficiently stiff to form freestanding shapes, so we could evaluate the robustness of the gel. Once cut, these hydrogels are able to heal in minutes at room temperature, which we attribute to the dynamic exchange between boronic acids and diols (Fig. 7a–d). We observed that gels formed after 1 hour of irradiation with green light remain gelled for at least one week when stored in the dark at 25 °C (Fig. S27†), consistent with *Z* isomers with long thermal half-lives.^17^ Attempts to swell the gels in solutions of PBS were consistent with a lightly crosslinked dynamic network: the material was fully dissolved after 6 hours at 25 °C (Fig. 7e–h). While this behavior represents an obstacle to long-term practical applications, Anseth and coworkers have previously demonstrated in dynamic hydrazone networks that replacing 4-arm PEG with 8-arm PEG significantly increases the lifetime of swollen gels.^18^ We expect that we will be able to rationally improve the long-term utility of these hydrogels by increasing branched polymer functionality and tuning the identity of the diol end-groups to increase *K*_eq_.

## Conclusions

In conclusion, we have discovered a platform to reversibly tune the mechanical properties of dynamic hydrogels that uses photoswitches to control the reactivity of dynamic covalent crosslinks. Small-molecule studies suggest that the conformation of the azobenzene boronic acid determines the equilibrium constant for condensation with diol, with an increased *K*_eq_ for the *Z* isomer. The increase in equilibrium constant generates a higher crosslink density in the hydrogel network, resulting in stiffening. Because of the dynamic nature of the boronic ester crosslink, these hydrogels are viscoelastic and stress-relaxing, and the stiffness can be tuned independently of stress relaxation rate. Importantly, we have already demonstrated that this approach can be generalized to an *o*-difluoroazobenzene with superior photophysical properties, enabling mechanical tuning solely with visible light. Future work will be directed at fully elucidating the molecular origins of the observed photocontrol, exploring the range of tunable mechanical properties provided by synthetic modifications of the boronic acid and diol, and adapting this platform for 4D cell culture.

## Conflicts of interest

J. A. K. and J. V. A. have filed a provisional patent application (U.S. Prov. 62/673,312).

## Supplementary Material

Supplementary information

Supplementary movie
