# Supplementary material for: Reversibly tuning hydrogel stiffness through photocontrolled dynamic covalent crosslinks
Source: Chem Sci. 2018 Jun 19;9(27):5987–93. doi: 10.1039/c8sc02093k (PMC6050525; doi:10.1039/c8sc02093k)
Supplement: Supplementary file 1 [file SC-009-C8SC02093K-s001.pdf]

Electronic Supplementary Material (ESI) for Chemical Science.
This journal is © The Royal Society of Chemistry 2018

Supporting Information

Reversibly Tuning Hydrogel Stiffness through Photocontrolled Dynamic Covalent Crosslinks

Joseph Accardo and Julia A. Kalow*

Department of Chemistry, Northwestern University
2145 Sheridan Rd, Evanston, IL 60208

E-mail: jkalow@northwestern.edu

Table of Contents

I. General information....2
II. Synthesis of azobenzene boronic acids....3
III. Small-molecule kinetics....9
IV. Functionalization of 4-arm PEG....23
V. Rheology....25
VII. NMR & IR Spectra....32
VIII. Photos....62
IX. References....64

I. General information

General procedures. Unless otherwise noted, reactions were performed under Ar atmosphere in oven-dried (120 ºC) glassware. Reaction progress was monitored by thin layer chromatography (Merk silica gel 60 F_{254} plates), visualizing with visualizing with fluorescence quenching, $KMnO_4$, or ninhydrin stains. Automated column chromatography was performed using SiliCycle SiliaFlash F60 (40-63 μm, 60 Å) in SNAP cartridges on a Biotage Isolera One. Organic solvents were removed *in vacuo* using a rotary evaporator (Büchi Rotovapor R-100, ~20–200 torr) and residual solvent was removed under high vacuum (<100 mtorr). Water-soluble polymers were purified by dialysis using SnakeSkin Dialysis Tubing (3.5 kDa cutoff, 16 mm diameter) purchased from Fisher.

Materials. Commercial reagents were purchased from Sigma-Aldrich, Acros, Alfa Aesar, TCI, or Oakwood and used as received. 4-Arm PEG-NH_2HCl salt (M_w 5 kDa) was purchased from JenKem, and was azeotroped with toluene (3x) and melted under high vacuum (<100 mtorr) prior to functionalization.

Instrumentation. Proton nuclear magnetic resonance (^{1}H NMR) spectra and carbon nuclear magnetic resonance (^{13}C NMR) spectra were recorded on Bruker AVANCE-500 spectrometers at 500 MHz and 125 MHz, and referenced to the solvent residual peaks. Boron nuclear magnetic resonance (^{11}B NMR) spectra were recorded on Bruker AVANCE-400 spectrometers at 128 MHz. BF_3•OEt_2 in $CDCl_3$ in a capillary was used as a reference for ^{11}B NMR (0 ppm) in Wilmad Precision NMR tubes (CFQ, 500 MHz, OD: 5 mm, wall thickness: 0.38 mm). NMR data are represented as follows: chemical shift (δ ppm), multiplicity (s = singlet, d = doublet, t = triplet, q = quartet, m = multiplet), coupling constant in Hertz (Hz), integration. UV-vis spectra were collected on a Cary 5000 UV-vis-NIR spectrophotometer with an Hg lamp; cuvettes were 10-mm path length quartz cells (Starna 23-Q-10). LCMS kinetics experiments was performed on a liquid chromatography-mass spectrometry system (Agilent 6120 Quadrupole LC/MS) equipped with a variable-wavelength detector. Infrared spectroscopy was performed on a Thermo Nicolet iS10 with a ZnSe crystal ATR attachment. Size exclusion chromatography (SEC) measurements were performed in stabilized, HPLC-grade tetrahydrofuran using an Agilent 1260 Infinity II system with variable-wavelength diode array (254, 450, and 530 nm) and refractive index detectors, guard column (Agilent PLgel; 5μm; 50 x 7.5 mm), and three analytical columns (Agilent PLgel; 5μm; 300 x 7.5 mm; 10^5, 10^4, and 10^3 Å pore sizes). The instrument was calibrated with narrow-dispersity polystyrene standards between 640 Da and 2300 kDa (Polymer Standards Service GmbH). All runs were performed at 1.0 mL/min flow rate and 40 ºC. Molecular weight values are calculated based on the refractive index signal. Blue LED strip lights (wavelength = 470 nm, power = 6.6 W) and green LED strip lights (wavelength = 525 nm, power = 5.7 W) were purchased from superbrightleds.com. The UV flashlight (wavelength = 365nm, power = 1.5 W) was purchased from American's Preferred. Light intensity was measured using an EXTECH LT45 LED light meter, and the light intensities for the blue and green LEDs were 900 and 600 lux respectively. The light intensity for the UV flashlight was calculated to be 3.6 mW/cm^2.

II. Synthesis of azobenzene boronic acids

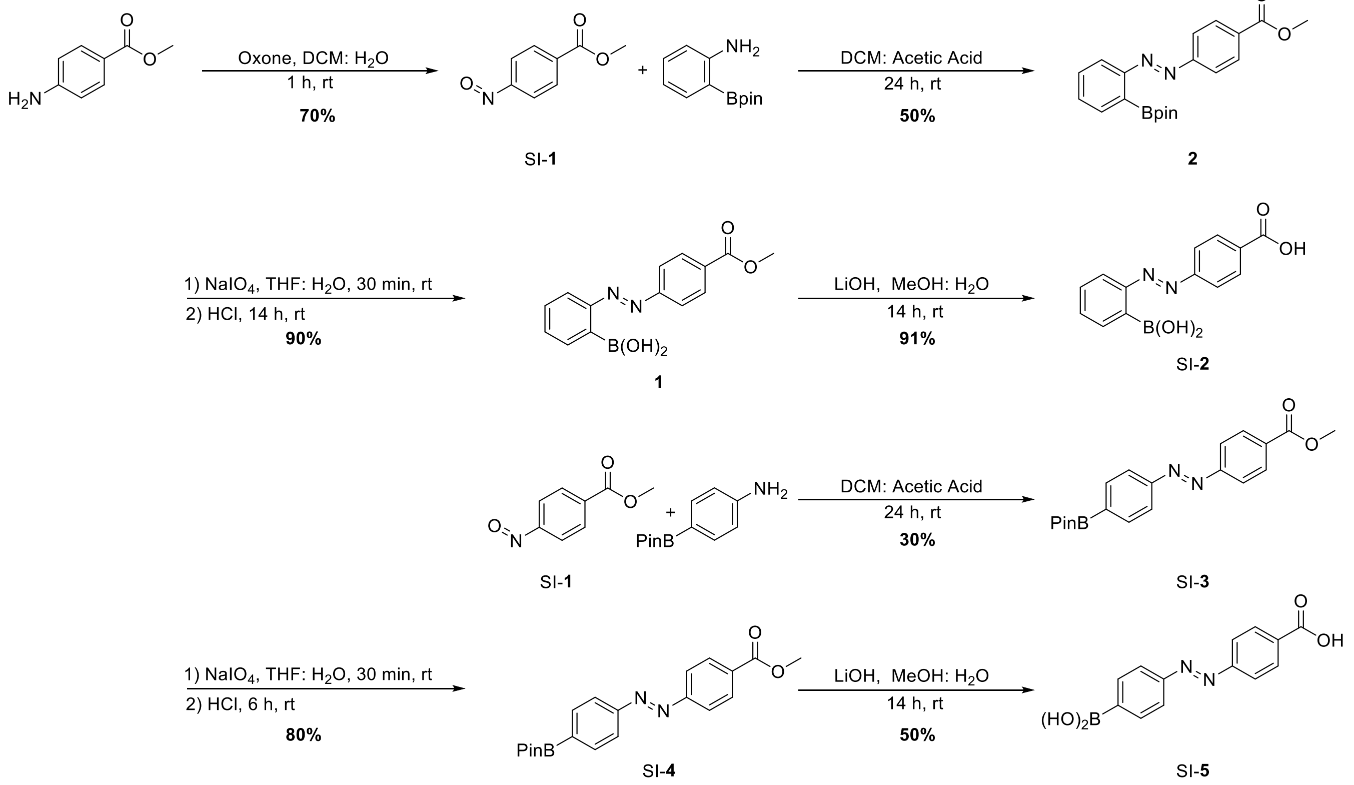

Scheme S1. Synthetic route of small molecules **1** and **2** for kinetic studies and compound SI-**2** and SI-**5** for synthesis of control polymers **P2** and **P3**.

methyl 4-nitrosobenzoate (SI-**1**)

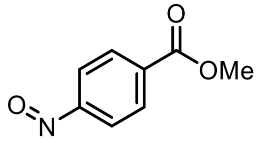

Based on a modified from literature procedure, methyl 4-aminobenzoate (3 g, 20 mmol) was dissolved in 66 mL of DCM into a 500-mL RBF equipped with stir bar.[1] Oxone (20 g, 40 mmol) was dissolved into 250 mL of deionized water and then added to the reaction mixture (capped with rubber septum which was pierced with a needle, exposed to air), and the reaction was aged for 1 hour with high stirring. Over the course of the reaction, the biphasic solution develops into an intense neon green color. The product was extracted against water (1x), 1M HCl (2x), and 1M NaOH (3x). The organic layer was dried over sodium sulfate and concentrated *in vacuo* to give methyl 4-nitrosobenzoate (2.26 g, 70%). This product was used in the subsequent step without further purification. ^{1}**H NMR** (500 MHz, Chloroform-*d*) δ 8.30 (d, J = 8.5 Hz, 2H), 7.94 (d, J = 8.5 Hz, 2H), 3.98 (s, 3H).

methyl (E)-4-((2-(4,4,5,5-tetramethyl-1,3,2,-dioxaborolan-2-yl)phenyl)diazinyl)benzoate (2)

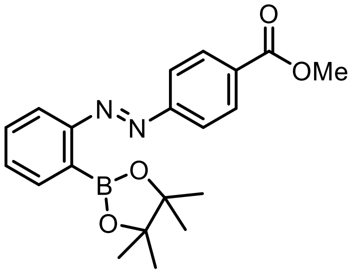

SI-**1** (4.4 g, 27 mmol) and 2-(4,4,5,5,-tetramethyl-1,3,2-dioxaborolan-2-yl)aniline (6.1 g, 28 mmol) were dissolved in 100 mL of DCM into a 250 mL RBF, to which 20 mL of acetic acid was added. The reaction was stirred for 24 hours, open to air. The solution turned black after several hours. Upon consumption of the starting material, the organic layer wash washed with 1M HCl (3x), and the organic layer was collected, dried with sodium sulfate, and concentrated *in vacuo*. Residual acetic acid was removed under high vacuum. The collected solids were diluted in hexane (300 mL), and the precipitate which formed (byproduct) was removed with a glass frit. The red filtrate was concentrated *in vacuo* to approximately 100 mL and then filtered again. The collected filtrate was concentrated *in vacuo* to yield an orange solid (~ 6 g) which was recrystallized from hexane (30 mL) twice to provide the product as a red crystalline solid (5 g, 50%). ^{1}**H NMR** (500 MHz, $CDCl_3$) δ 8.19 (d, *J* = 8.7 Hz, 2H), 7.94 (d, *J* = 8.6 Hz, 2H), 7.81 (d, *J* = 8.0, 1.1 Hz, 1H), 7.75 (d, *J* = 1.5 Hz, 1H), 7.56 – 7.52 (m, 1H), 7.50 – 7.46 (m, 1H), 3.96 (s, 3H), 1.36 (s, 12H). ^{13}**C NMR** (125 MHz, $CDCl_3$) δ 166.6, 156.9, 155.4, 134.5, 131.5, 130.8, 130.6, 130.5, 122.8, 119.7, 84.0, 52.3, 25.0. ^{11}**B NMR** (128 MHz, $CDCl_3$) 31.3. **IR** (cm^{-1}) 2977, 2953, 2925, 2851, 1721, 1597. **HRMS** m/z expected for $C_{20}H_{24}BN_2O_4$ $[M+H]^+$ 367.18, measured 367.18.

(E)-(2-((4-(methoxycarbonyl)phenyl)diazinyl)phenyl)boronic acid (1)

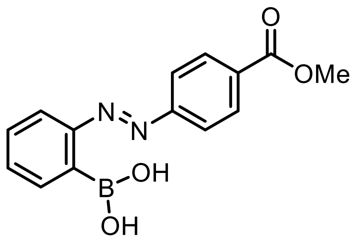

2 (5 g, 10 mmol) was dissolved into a 500 mL RBF equipped with a stir bar (capped with a rubber septum which was pierced with a needle, exposed to air). The starting material was dissolved in THF (250 mL), providing a deep clear red solution. Sodium periodate (9 g, 40 mmol) was dissolved in DI water (62 mL) and transferred to the solution. After stirring for 30 minutes, 1M HCl (8 mL) was added. The reaction was stirred overnight at room temperature. In the morning the solution was red and cloudy with precipitate. The THF was removed *in vacuo* to concentrate the product in water. The resulting suspension was diluted with additional water and filtered to provide an orange/ peach colored solid. The solids were washed with acetonitrile (100 mL) and collected and dried on a glass filter to give the **1** (3.5 g, 90%) as a peach-colored solid. This product was used in the subsequent step without further purification. ^{1}**H NMR** (500 MHz, DMSO-d_6) δ 8.19 (d, *J* = 8.4 Hz, 2H), 7.96 (d, *J* = 8.4 Hz, 2H), 7.92 (d, *J* 6.3 Hz, 1H), 7.83 (s, 2H), 7.59 – 7.53 (m, 3H), 3.91 (s, 3H). ^{13}**C NMR** (126 MHz, DMSO-d_6) δ 166.1, 155.0, 154.1, 133.1, 131.9, 131.8, 131.0, 129.6, 123.6, 123.0, 52.9. ^{11}**B NMR** (128 MHz, DMSO-d_6) 28.4. **IR** (cm^{-1}) 3347 (br), 3198 (br), 3033, 2962, 1723, 1597. **LRMS** m/z expected for $C_{14}H_{14}BN_2O_4$ $[M+H]^+$ 285.10, measured 285.1.

(E)-4-((2-boronophenyl)diazenyl)benzoic acid (SI-**2**)

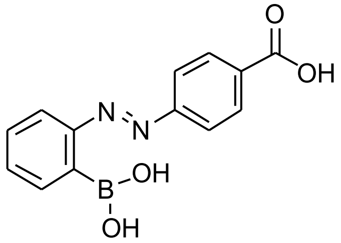

1 (3 g, 10 mmol) was dissolved in MeOH (282 mL) in a 1-L RBF equipped with a stir bar, providing a bright red solution. Lithium hydroxide (1 g, 40 mmol) was dissolved into water (58 mL) and transferred to the reaction mixture, which was stirred open to air overnight. The methanol was removed *in vacuo*, providing a red alkaline aqueous solution. This solution was neutralized with 1M HCl to precipitate the desired product, which was collected on a glass filter and dried in a vacuum oven (100 °C) for 1 hr to provide the title product (2.45 g, 91%) as a peach-colored solid. This product was used in the subsequent step without further purification. **^{1}H NMR** (500 MHz, DMSO-*d*$_6$) δ 13.25 (s, 1H), 8.17 (m, 2H), 7.98 – 7.90 (m, 3H), 7.82 (s, 2H), 7.62 – 7.52 (m, 3H). **^{13}C NMR** (125 MHz, DMSO-*d*$_6$) δ 167.2, 155.1, 154.0, 133.2, 133.1, 131.7, 131.1, 129.5, 123.5, 122.9. **^{11}B NMR** (128 MHz, DMSO-*d*$_6$) 28.6. **IR(cm^{-1})** 3395 (br), 3106, 3064, 2359, 2339, 2161, 1682, 1601. **LRMS** m/z expected for $C_{13}H_{10}BN_2O_4$ [M-H]$^-$ 269.07, measured 269.1.

(E)-4-((4-(4,4,5,5-tetramethyl-1,3,2-dioxaborolan-2-yl)phenyl)diazenyl)benzoate (SI-**3**)

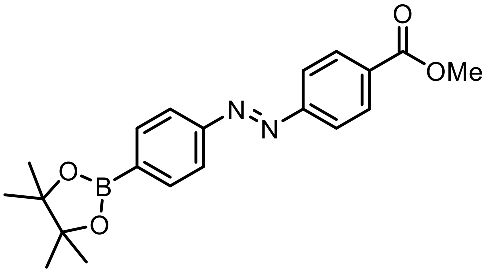

SI-**1** (1.1 g, 6.60 mmol) and 4-(4,4,5,5-tetramethyl-1,3,2-dioxaborolan-2-yl)aniline (1.6 g, 7.3 mmol) were dissolved in DCM (100 mL) in a 200 mL RBF. Acetic acid (5 mL) was added dropwise. Initially the solution was neon green, but over the course of 24 hours the reaction turned dark red. Upon completion, the reaction was extracted against water (1x), 1M HCl (1x), and brine (1x). The combined organics were concentrated *in vacuo* and dissolved in DCM, which was passed through a silica plug and concentrated *in vacuo*. The resulting product was recrystallized from 9:1 hexane:EtOAc to provide the title product (712 mg, 30%). **^{1}H NMR** (500 MHz, $CDCl_3$) δ 8.20 (d, J = 8.6 Hz, 2H), 8.01 – 7.95 (m, 4H), 7.93 (d, J = 8.3 Hz, 2H), 3.96 (s, 3H), 1.38 (s, 12H). **^{13}C NMR** (125 MHz, $CDCl_3$) δ 166.5, 155.2, 154.2, 135.7, 131.9, 130.6, 122.7, 122.2, 84.2, 52.4, 24.9. **^{11}B NMR** (128 MHz, $CDCl_3$) 30.1. **IR**(cm^{-1}) 2978, 2956, 2928, 2361, 2343, 1716, 1601. **HRMS**: m/z expected for $C_{20}H_{24}BN_2O_4$ [M+H]$^+$ 367.18, measured 367.18.

(E)-(4-((4-(methoxycarbonyl)phenyl)diazenyl)phenyl)boronic acid (SI-**4**)

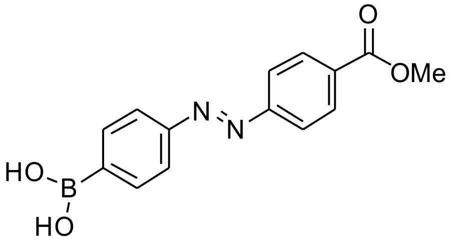

SI-**3** (510 mg, 1.39 mmol) was dissolved in 25 mL of THF into a 100 mL RBF equipped with a stir bar to give a red colored solution. Sodium periodate (298 mg, 1.39 mmol) was dissolved in water (6.2 mL) and added to the reaction. After 30 minutes, 1 mL of 1 M HCl was added to the solution, which was stirred for a further 6 hours. Upon full conversion, the THF was removed *in vacuo* leading to the precipitation of product, which was collected on a glass frit. The solids were washed with water (3 x 10 mL) and acetonitrile (10 mL) and dried to provide the title product (320 mg, 81%) as a peach-colored solid. **^{1}H NMR** (500 MHz, DMSO-*d$_6$*) δ 8.30 (s, 2H), 8.18 (d, J = 8.2 Hz, 2H), 8.05 – 7.99 (m, 4H), 7.90 (d, J = 7.9 Hz, 2H), 3.92 (s, 3H). **^{13}C NMR** (125 MHz, DMSO-*d$_6$*) δ 166.1, 154.5.0, 153.4, 135.7, 132.1, 131.0, 123.2, 122.2, 52.9. **^{11} B NMR** (128 MHz, DMSO-*d$_6$*) 26.8. **IR** (cm^{-1}) 3346 (br), 2965, 2358, 1724, 1604. **HRMS** m/z expected for $C_{14}H_{14}BN_2O_4$ [M+H]$^+$ 285.10, measured 285.10.

(E)-4-((4-boronophenyl)diazenyl)benzoic acid (SI-**5**)

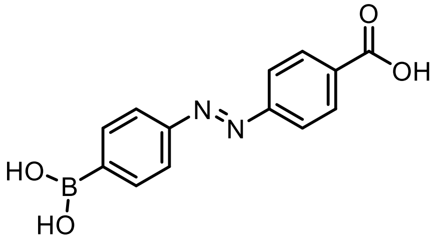

SI-**4** (1.19 g, 4.19 mmol) was dissolved in methanol (112 mL) in a 250-mL RBF equipped with a stir bar. Lithium hydroxide (0.401 g, 16.8 mmol) was dissolved in water (23 mL) and added to the solution, which was stirred overnight. After 14 hours, the reaction was as an orange solution. The methanol was removed *in vacuo* and then 1M NaOH was added to the crude mixture to dissolve the solids. The aqueous solution was washed with EtOAc and then neutralized with 1M HCl, leading to the precipitation of the product. The solids were filtered, washed with water, and dried to provide the title product (160 mg, 50%) as a peach-colored solid. **^{1}H NMR** (500 MHz, DMSO-*d$_6$*) δ 8.10 (d, J = 8.2 Hz, 2H), 7.99 (d, J = 7.9 Hz, 2H), 7.89 (d, J = 8.1 Hz, 2H), 7.86 (d, J = 8.1 Hz, 2H).**^{13}C NMR** (125 MHz, DMSO-*d$_6$*) δ 168.7, 1538, 153.5, 138.3, 135.6, 130.8, 122.6, 122.0. **^{11} B NMR** (128 MHz, DMSO-*d$_6$*) 26.5. **IR** (cm^{-1}) 3406 (br), 2359, 1692, 1601. **HRMS** m/z expected for $C_{13}H_{10}BN_2O_4$ [M-H]$^-$ 269.07, measured 269.07.

4-amino-3,5-difluorobenzoic acid

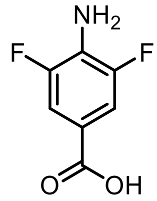

4-Amino-3,5-difluorobenzoic acid was synthesized by following a previously reported procedure. Characterization data were consistent with literature reports.[2]

methyl 4-amino-3,5-difluorobenzoate (SI-**6**)

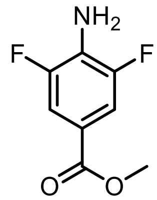

4-Amino-3,5-difluorobenzoic acid (500 mg, 2.89 mmol) was dissolved in 20 mL of MeOH to which 1.75 mL of concentrated sulfuric acid was added to. The reaction was heated to reflux for 14 hours. After the reaction the solvent was removed *in vacuo* and 20 mL of water was added, leading the precipitation of the product, which was collected and dried on a funnel to provide the title product (417 mg, 77%) as a white solid, which was used in the next reaction without further purification. **^{1}H NMR** (500 MHz, $CDCl_3$) δ 7.58 – 7.46 (m, 2H), 4.14 (s, 2H), 3.87 (s, 3H).

methyl 3,5-difluoro-4-nitrosobenzoate (SI-**7**)

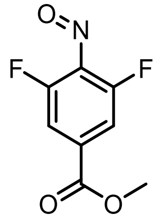

Methyl 4-amino-3,5-difluorobenzoate (2.52 g, 13.5 mmol) was dissolved in DCM (60 mL) into a 500 mL RBF equipped with a stir bar and oxone (30.2 g, 49.1 mmol) in water (240 mL) was added to the solution which was left to react for 24 hours. The organic layer was washed with 1M HCl (1x), 1M NaOH (1x), and DI water (1x). his was concentrated to give the title product (2.05 g, 76%) as a yellow solid, which was used in the next reaction without further purification.

(*E*)-(2-((2,6-difluoro-4-(methoxycarbonyl)phenyl)diazenyl)phenyl)boronic acid (SI-**8**)

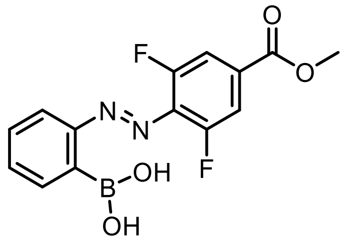

SI-**7** and 2-(4,4,5,5-tetramethyl-1,3,2-dioxaborolan-2-yl)aniline (2.23 g, 10.2 mmol) were weighed into a 100 mL RBF equipped with a stir rod under inert atmosphere. DCM (70 mL) was added, and the dissolution of both starting materials led to the development of a dark-colored solution. Acetic acid (12 mL) was added and the solution was left to react for 24 hours. After 24 hours, the solvent was removed in vacuo and with high vacuum (to remove excess acetic acid), and the crude residue was diluted in 100 mL of hexane and filtered to provide the protected crude (2.17 g) as a red solid. This material was dissolved in 130 mL of THF into a 250 mL RBF equipped with a stir rod. Sodium periodate (3.46 g, 16.2 mmol) was added in 30 mL of water and the solution was left to react for 14 hours. After the reaction, the THF was removed in vacuo, and the remaining crude was diluted with 100 mL of DCM. The product precipitated from solution and was collected on a Buchner funnel, which was washed with an additional 100 mL of DCM to yield the title product (1.12 g, 34%) as a dark orange solid. **^{1}H NMR** (500 MHz, DMSO-d6) δ 7.99 (s, 2H), 7.87 – 7.80 (m, 3H), 7.71 – 7.67 (m, 1H), 7.63 – 7.57 (m, 2H), 3.92 (s, 3H). **^{13}C NMR** (125 MHz, DMSO-*d$_6$*) δ 164.2, 156.1, 156.0, 154.1, 134.2, 132.9, 130.2, 120.5, 114.4, 114.2, 53.5 **^{19}F NMR** (# MHz, DMSO-*d$_6$*) δ -116.6 (*E*) , -119.8 (*Z*) **IR** (cm^{-1}) 3266 (br), 2361, 1724, 1592. **LRMS** m/z expected for $C_{14}H_{11}BF_2N_2O_4$ [M+H]$^+$ 321.08, measured 321.1.

(*E*)-4-((2-boronophenyl)diazenyl)-3,5-difluorobenzoic acid (SI-**9**)

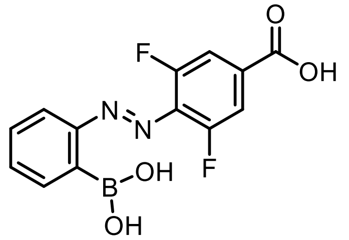

SI-**8** (70 mg, 0.22 mmol) was dissolved in 7 mL of MeOH into a 25 mL RBF equipped with a stir bar to which LiOH (16 mg, 0.66 mmol) in 3 mL of water was added. The reaction was left to react for 14 hours. After the reaction, the methanol was removed in vacuo and the water was neutralized with 1M HCl, leading to the precipitation of the product. The product was dissolved in EtOAc and washed with water. The organic layer was dried with sodium sulfate and then concentrated in vacuo to give the title product (64.4 mg, 96%) as an orange solid. **^{1}H NMR** (500 MHz, DMSO-d6) δ 7.99 (s, 2H), 7.83 (d, J = 7.4 Hz, 1H), 7.79 (d, J = 9.5 Hz, 2H), 7.69 (s, 1H), 7.60 (tt, J = 7.3, 5.6 Hz, 2H) **^{13}C NMR** (125 MHz, DMSO-d6) δ 165.2, 156.1, 156.0, 154.1, 134.2, 132.8, 130.2, 120.2, 114.3, 114.1 **^{19}F NMR** (# MHz, DMSO-*d$_6$*) δ -117.0 (*E*) , -120.1 (*Z*) **IR** (cm^{-1}) 3361 (br), 3053, 2358, 1720, 1595. **LRMS** m/z expected for $C_{13}H_9BF_2N_2O_4$ [M+H]$^+$ 306.06, measured 307.0.

III. Small-molecule kinetics

Procedure for calibration curves

Stock solutions of *(E)*-**1** or *(E)*-**2** were prepared in acetonitrile at 800 μM. Serial dilutions were performed to obtain a final concentration of 12.5 μM. The solutions were directly injected onto the LCMS (Poroshell 120 column, EC-C18, 2.7 μm, 2.1 x 50 mm). Runs were ten minutes long with solvent gradient from 50:50 to 100:0 ACN:H_2O in the first three minutes. Detection was performed at 254 nm with a variable-wavelength detector. The peak area for each concentration was recorded. A calibration curve was created. The *Z*-isomer calibration curves were prepared by irradiating the series above for 10 minutes with 365 nm UV light and directly injecting the solution onto the LCMS. The concentration of the *Z*-isomer can be calculated from the known concentration of the *E*-isomer (based on the previously-generated calibration curve).

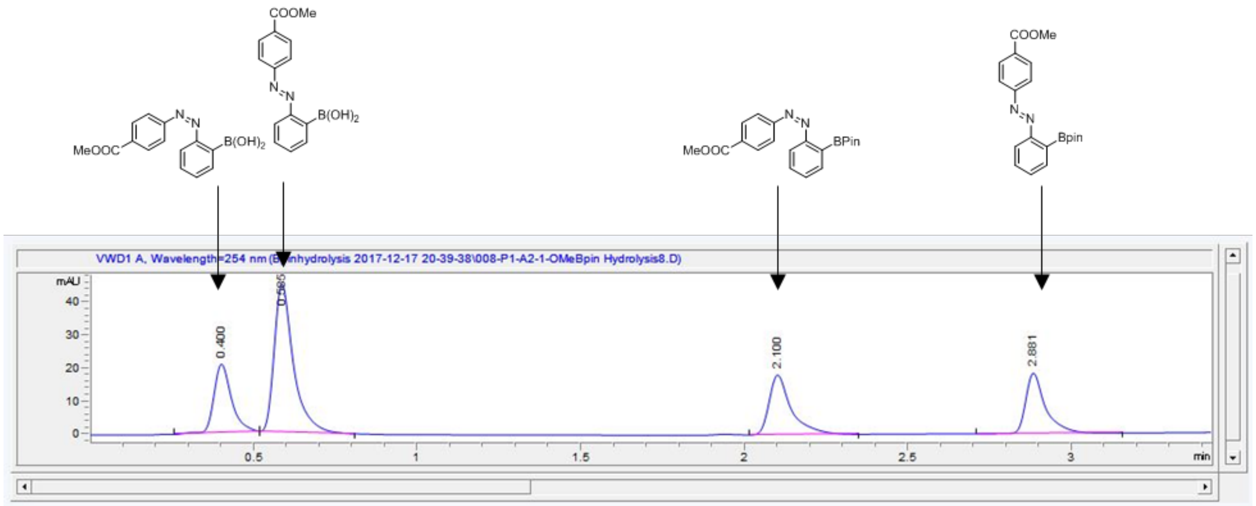

File Information	
LC-File	008-P1-A2-1-OMeBpin Hydrolysis8.D
File Path	C:\Chem32\1\Data\Bpinhydrolysis 2017-12-17 20-39-38
Date	17-Dec-17, 21:54:02
Sample	1-OMeBpin Hydrolysis8
Sample Info	
Barcode	

#	Time	Type	Area	Height	Width	Area%	Symmetry
1	0.4	BB	78.7	20.9	0.0558	18.463	0.738
2	0.585	BB	184	45.2	0.0605	43.198	0.613
3	2.1	BB	86.2	18.3	0.0701	20.237	0.558
4	2.881	BB	77.1	18.4	0.0608	18.102	0.574

ed Joseph Accardo (localhost) LCMS 0 Ready

Figure S1: A mixture of *(Z)*-**1** (retention time 0.4 min), *(E)*-**1** (retention time 0.585 min), *(Z)*-**2** (retention time 2.1 min) and *(E)*-**2** (retention time 2.881 min).

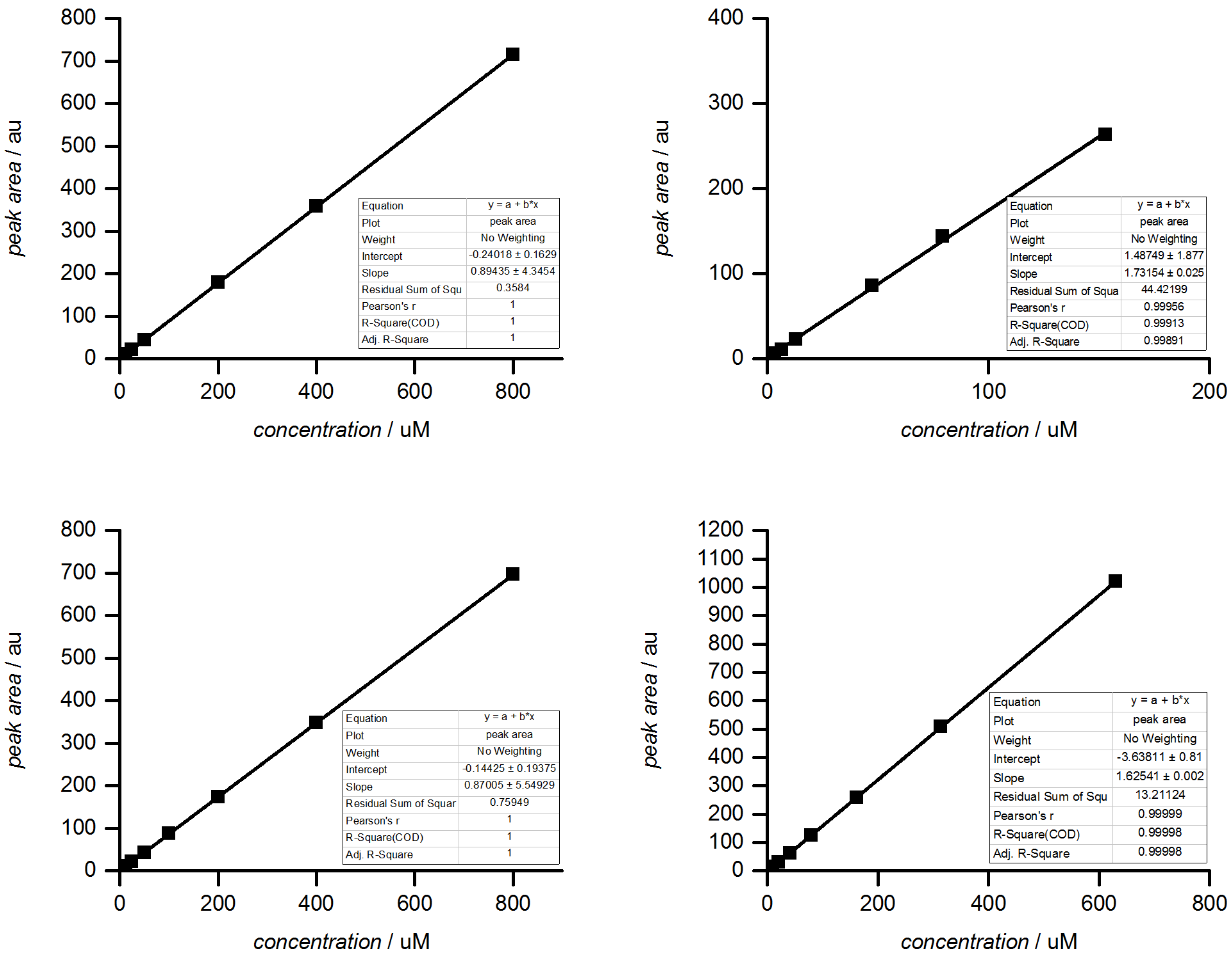

Figure S2. Calibration curves of *(E)*- **2** (top left), of *(Z)*- **2** (top right), *(E)*- **1** (bottom left), and *(Z)*- **1** (bottom right) prepared in acetonitrile.

Pseudo-first order reversible esterification of *(E)*-1

500 µL of a stock solution of 800 µM *(E)*-**1** was diluted with 500 µL of 80 mM pinacol in deionized H_2O. The concentration of *(E)*-**1** and *(E)*-**2** were monitored as a function of time using the above LCMS method. Because both water and pinacol were present in excess, we used pseudo-first-order approximations. Apparent rate and equilibrium constants were determined using the derivation shown below.

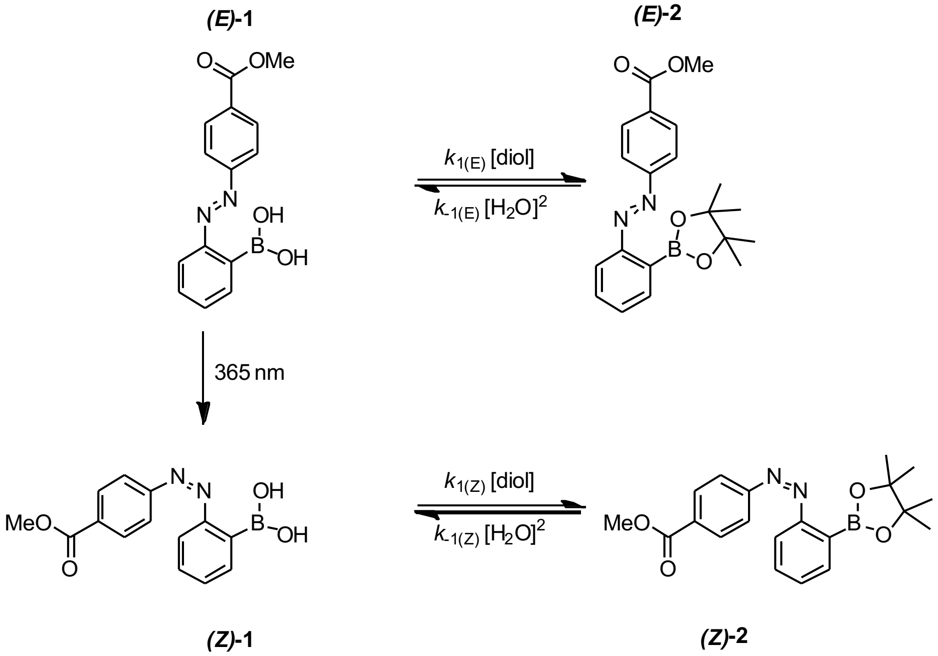

Scheme S2. Chemical structures for the pseudo-first order reversible esterification of *(E)*-**1** and/or *(Z)*-**1**. The photochemical E/Z isomerization is drawn as irreversible due to the slow thermal relaxation of *(Z)*-***1*** and *(Z)*-**2**. This also allows for the simplification of the derivation of observed rate constants, shown below.

The data from the concentration vs. time experiments were plotted according to equation (1) for a reversible first order reaction[(2)] and the slope of the best-fit line was determined in Origin (Scheme S4). The apparent equilibrium constant (K'_{eq}) for the reaction is the ratio of *(E)*-**2** to *(E)*-**1** at equilibrium, which can also be represented as the ratio of apparent rate constants (k') for the forward (esterification, k'_1) and reverse (hydrolysis, k'_{-1}) reactions, as shown in equation (2). Equation (2) can be rewritten as equation (3). With the apparent equilibrium constant and slope known, the apparent rate constant for hydrolysis (k'_{-1}) can be determined with equation (5), and subsequently the apparent rate constant for esterification (k'_1) can be determined by plugging these values into equation 4.

$$\ln([(E)\text{-}\mathbf{2}]_e - \ln[(E)\text{-}\mathbf{2}]_t) - \ln([(E)\text{-}\mathbf{2}]_e - \ln[(E)\text{-}\mathbf{2}]_0) = (k'_{1(E)} - k'_{-1(E)})t \quad (1)$$

$$\text{where } \; k'_{-1(E)} = k_{-1(E)}[\mathrm{H_2O}]^2 \; \text{ and } \; k'_{1(E)} = k_{1(E)}[\text{diol}]$$

$$K'_{eq(E)} = \frac{k'_{1(E)}}{k'_{-1(E)}} \quad (2)$$

$$k'_{-1(E)}K'_{eq(E)} = k'_{1(E)} \quad (3)$$

$$k'_{-1(E)} + k'_{1(E)} = \text{slope} \quad (4)$$

$$k'_{-1(E)} = \frac{\text{slope}}{1+K'_{eq(E)}} \quad (5)$$

Definition of equilibrium and apparent equilibrium constants:

$$K_{eq} = \frac{[\mathbf{2}][\mathrm{H_2O}]^2}{[\mathbf{1}][\mathrm{diol}]} = \frac{k_1}{k_{-1}} \quad (6)$$

$$\text{rate}_1 = k_1[\mathbf{1}][\mathrm{diol}] = k'_1[\mathbf{1}] \quad \text{so} \quad k_1 = \frac{k'_1}{[\mathrm{diol}]} \quad (7)$$

$$\text{rate}_{-1} = k_{-1}[\mathbf{2}][\mathrm{H_2O}]^2 = k'_{-1}[\mathbf{2}] \quad \text{so} \quad k_{-1} = \frac{k'_{-1}}{[\mathrm{H_2O}]^2} \quad (8)$$

$$\text{thus} \quad K_{eq} = \frac{k'_1/[\mathrm{diol}]}{k'_{-1}/[\mathrm{H_2O}]^2} = \frac{k'_1[\mathrm{H_2O}]^2}{k'_{-1}[\mathrm{diol}]} = K'_{eq}\frac{[\mathrm{H_2O}]^2}{[\mathrm{diol}]} \quad (9)$$

In Table 1, the *k* and *K* values are apparent rate and equilibrium constants (shown as *k'* and *K'* in the derivation above).

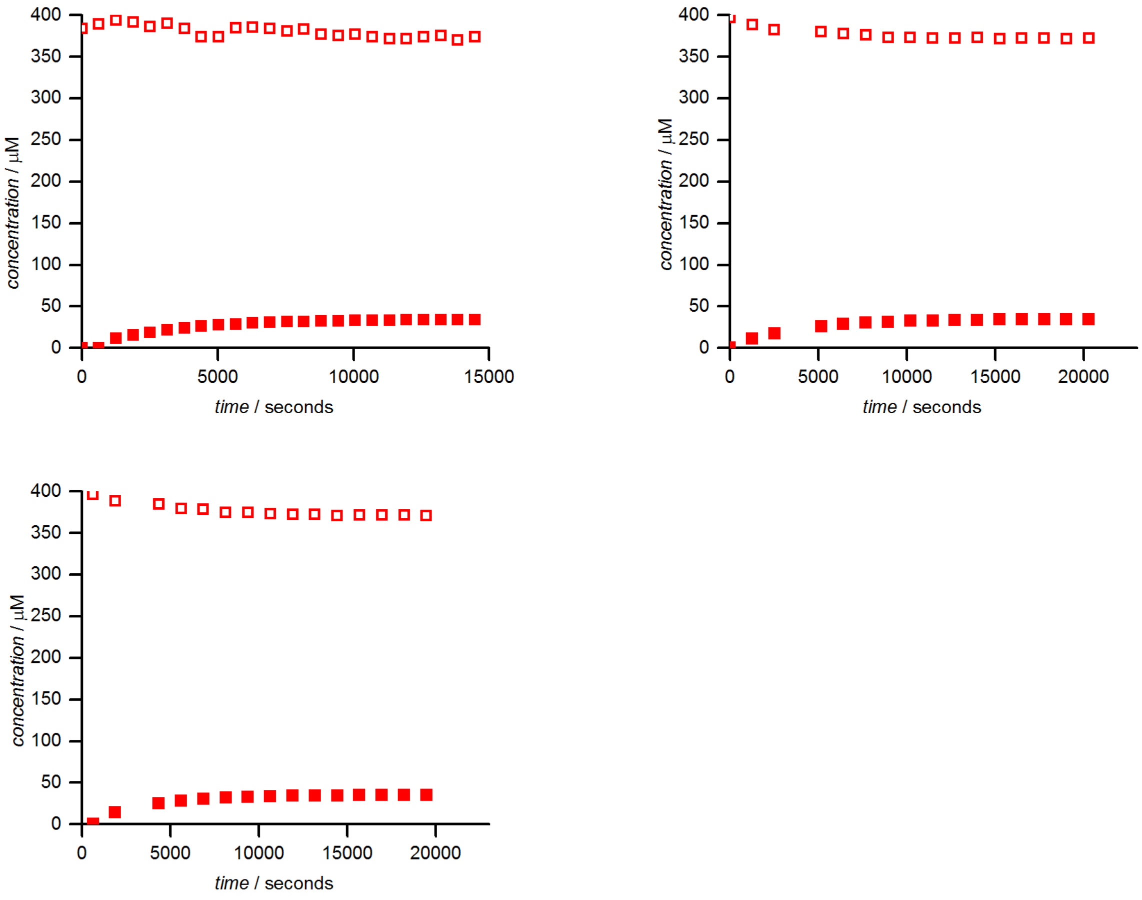

Figure S3. Concentration vs. time graphs for the reversible pseudo-first order esterification of 400 µM *(E)*-**1** and with 40 mM of pinacol in 1:1 acetonitrile:water. Solid red squares represent *(E)*-**2** and hollow red squares represent *(E)*-**1**.

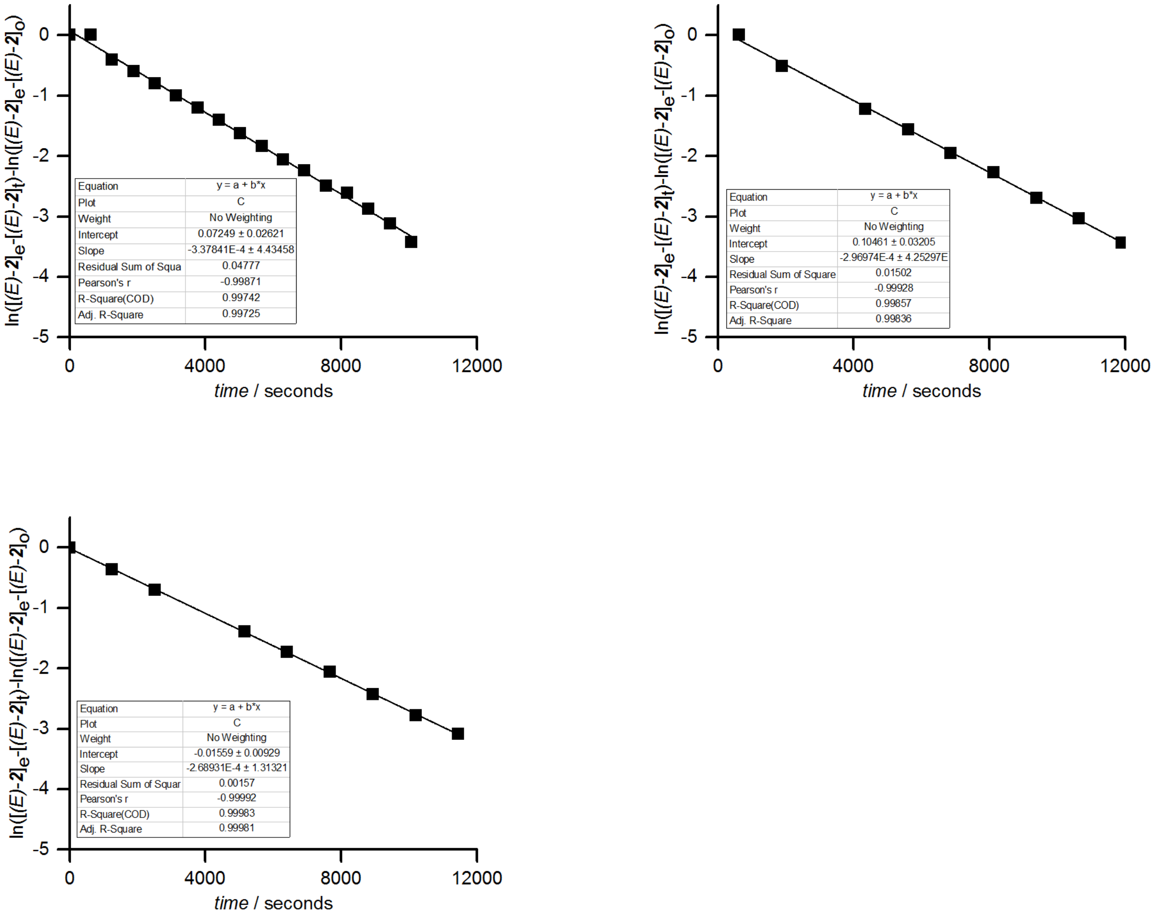

Figure S4. Linear fits for the reversible pseudo-first order esterification of 400 μM *(E)*-**1** and with 40 mM of pinacol in 1:1 acetonitrile: water, where the slope is equal to $-(k_{1(E)} + k_{-1(E)})$.

Pseudo-first order reversible esterification of *(Z)*-**1** and *(E)*-**1**

500 µL of a stock solution of 800 µM of *(E)*-**1** was irradiated with a 365 nm flashlight for 10 minutes to produce a mixture of *(E)*-**1** and *(Z)*-**1**. The mixture was then diluted with 500 µL of a 80 mM pinacol solution in deionized H_2O, and the concentration of *(E)*-**2** and *(Z)*-**2** were monitored as a function of time using the above LCMS method. The observed rate constants of esterification and hydrolysis $k_{1(Z)}$ and $k_{-1(Z)}$ were solved for using the same derivation as shown above for the *E* isomer. A small increase in the total concentration of (*E*)-**1** and (*E*)-**2** over the course of this experiment is ascribed to thermal relaxation of the (*Z*) isomers.

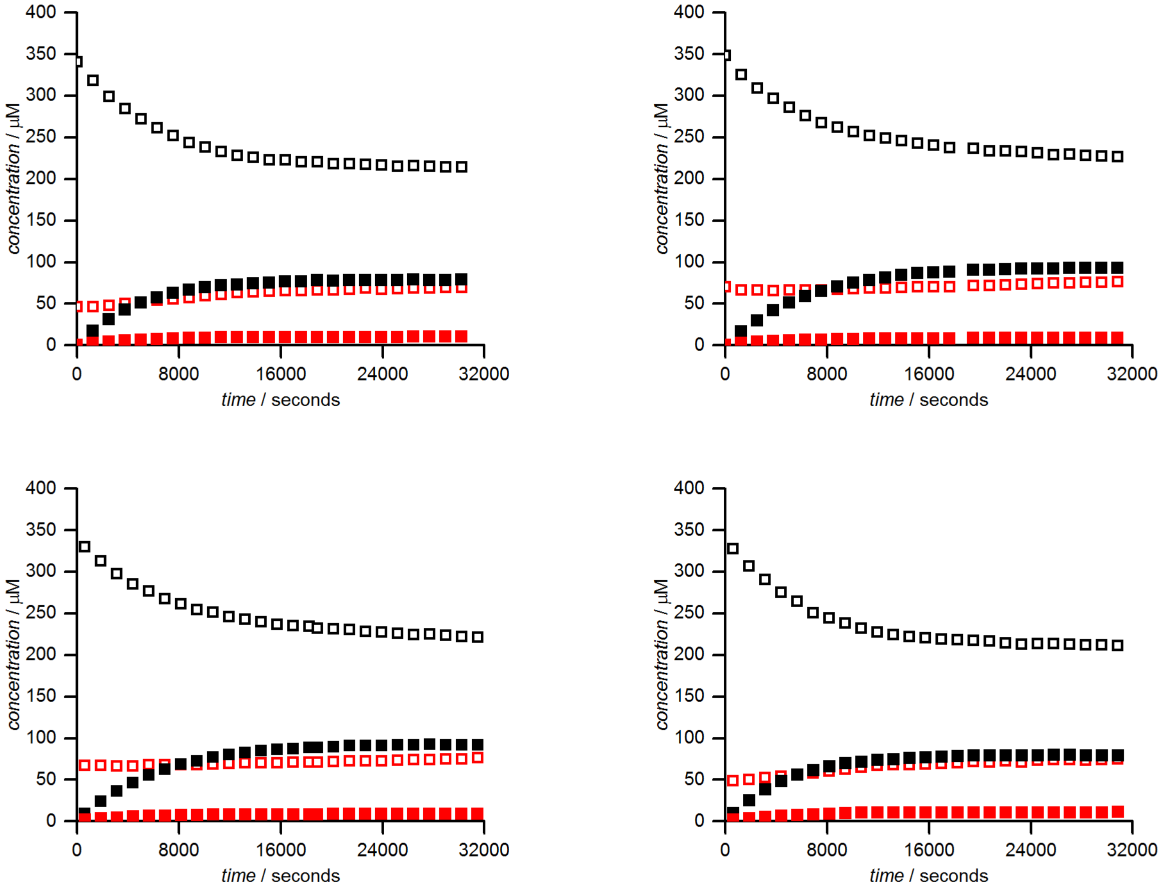

Figure S5. Concentration vs. time graphs for the reversible pseudo-first order esterification of 400 µM *(E)*-**1** and *(Z)*-**1** with 40 mM of pinacol in 1:1 acetonitrile: water. Solid red squares represent *(E)*-**2** and hollow red squares represent *(E)*-**1**. Solid black squares represent *(Z)*-**2** and hollow black squares represent *(Z)*-**1**.

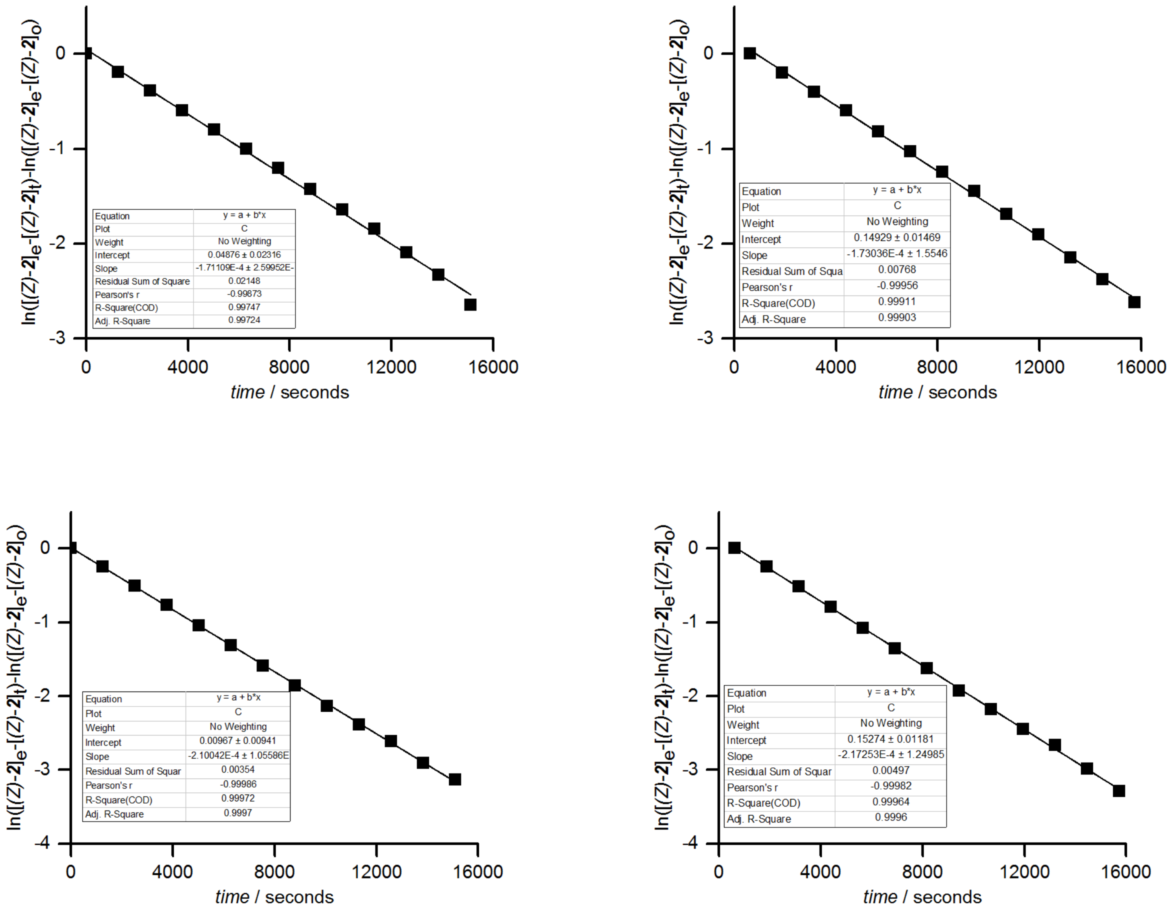

Figure S6. Linear fits for the reversible pseudo-first order esterification of 400 µM *(E)*-**1** and *(Z)*-**1** with 40 mM of pinacol in 1:1 acetonitrile: water, where the slope is equal to $-(k_{1(Z)} + k_{-1(Z)})$.

Pseudo-first order irreversible hydrolysis of *(Z)*-**2** and *(E)*-**2**

500 µL of a stock solution of 800 µM of *(E)*-**2** was irradiated with a 365 nm flashlight for 10 minutes to produce a mixture of *(E)*-**2** and *(Z)*-**2**. The mixture was then diluted with 500 µL of DI H_2O and the concentration of *(E)*-**2** and *(Z)*-**2** was monitored as a function of time. The observed rate constants of hydrolysis $k_{-1(E)}$ and $k_{-1(Z)}$ were determined using the derivation shown below.

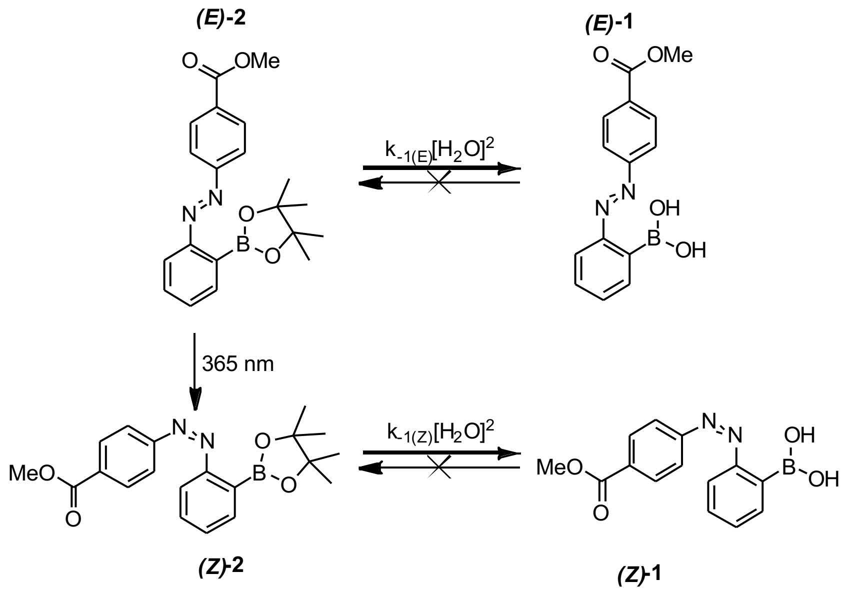

Scheme S3. Chemical structures for the pseudo-first order irreversible hydrolysis of *(E)*-**2** and/or *(Z)*-**2**. The photochemical E/Z isomerization is drawn as irreversible due to the slow thermal relaxation of (Z)-**1** and (Z)-**2**. This also allows for the simplification of the derivation of observed rate constants, shown below.

The concentrations vs. time can be fit to the linear expression (6) for an irreversible first order reaction where the slope is the apparent rate constant for the respective isomer.[3]

$$\ln[(E)\text{-}\mathbf{2}]_t - \ln[(E)\text{-}\mathbf{2}]_0 = -k'_{-1(E)}t \quad \text{where} \quad k'_{-1(E)} = k_{-1(E)}[\mathrm{H_2O}]^2 \quad (10)$$

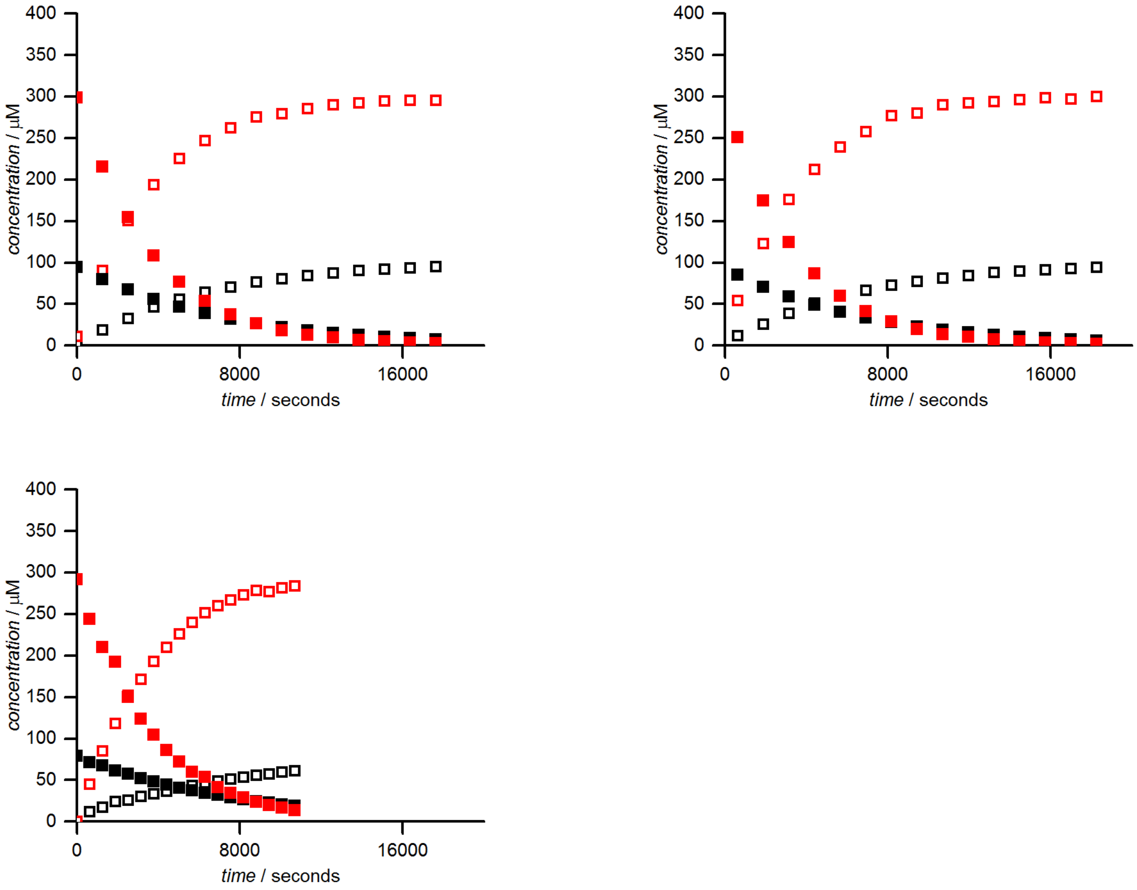

Figure S7. Concentration vs. time for the irreversible pseudo-first order hydrolysis of 400 µM *(E)*-**2** and *(Z)*-**2** with in 1:1 acetonitrile: water. Solid red squares represent *(E)*-**2** and hollow red squares represent *(E)*-**1**. Solid black squares represent *(Z)*-**2** and hollow black squares represent *(Z)*-**1.**

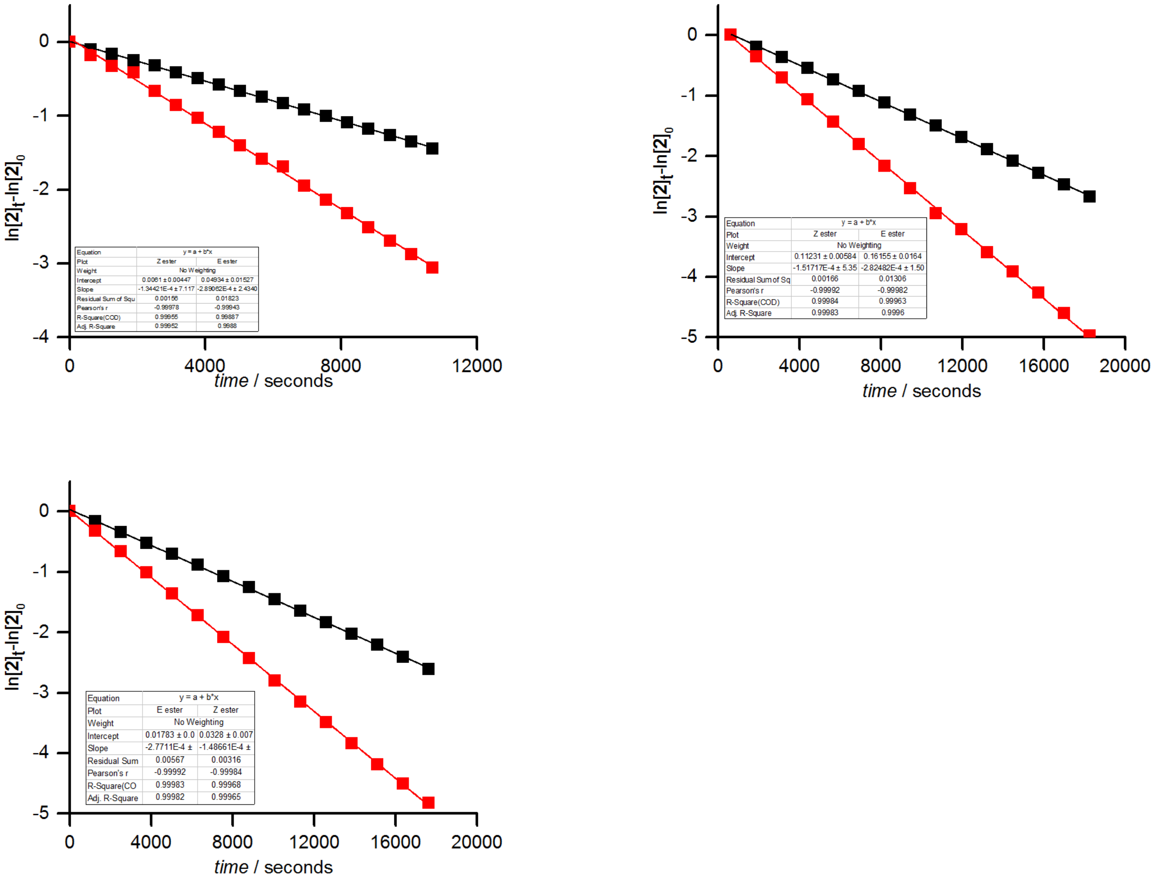

Figure S8. Linear fits for the irreversible pseudo-first order hydrolysis of 400 μM *(E)*-**2** and *(Z)*-**Z** in 1:1 acetonitrile: water, where the slope is equal to $-k_{-1(E)}$ or $-k_{-1(Z)}$. Red squares represent *(E)*-**2** while black squares represent *(Z)*-**2.**

Determination of the thermal half-life of (*Z*)-1

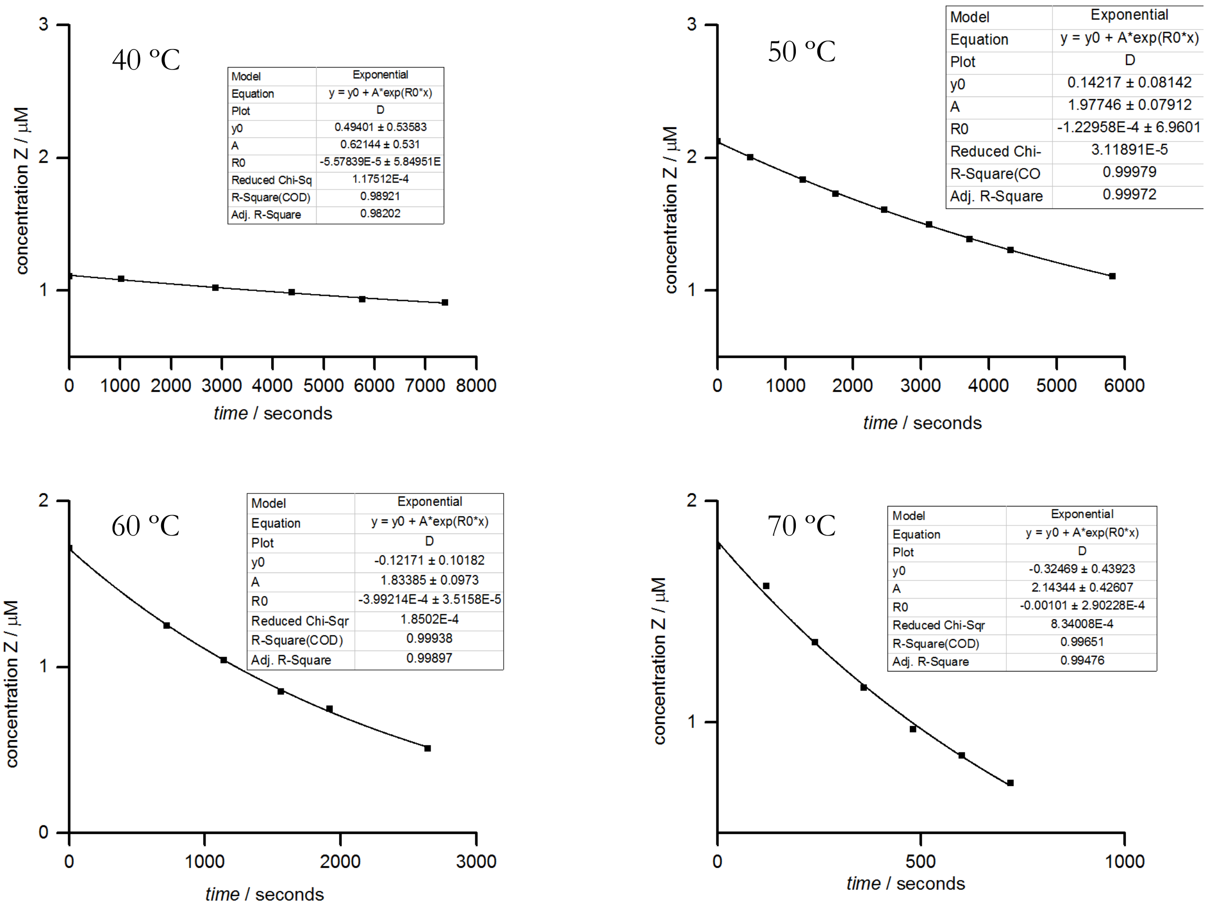

Figure S9. Concentration vs. time for the thermal relaxation of *(Z)*-**1** to *(E)*-**1**, as determined by ^{1}H NMR, at 40 °C (top left), 50 °C (top right), 60 °C (bottom left) and 70 °C (bottom right).

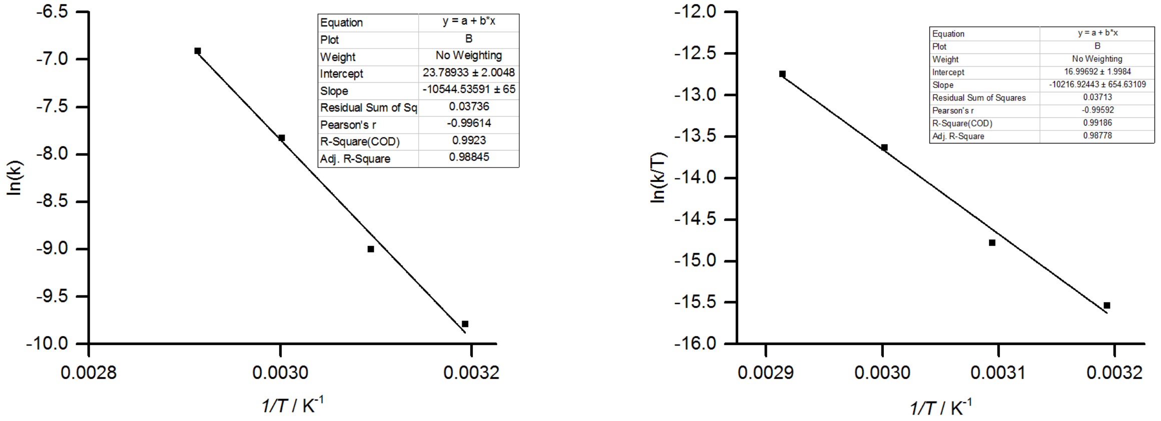

Figure S10. Left: Arrhenius plot for the thermal relaxation of *(Z)*-**1** to *(E)*-**1** . Right: Eyring plot for the thermal relaxation of *(Z)*-**1** to *(E)*-**1**. From the Arrhenius Plot, the half-life of the thermal relaxation of *(Z)*-**1** to *(E)*-**1** (determined to be a first order process by plotting ln(*k*) vs time), was determined to be 21 hours at 25 °C.

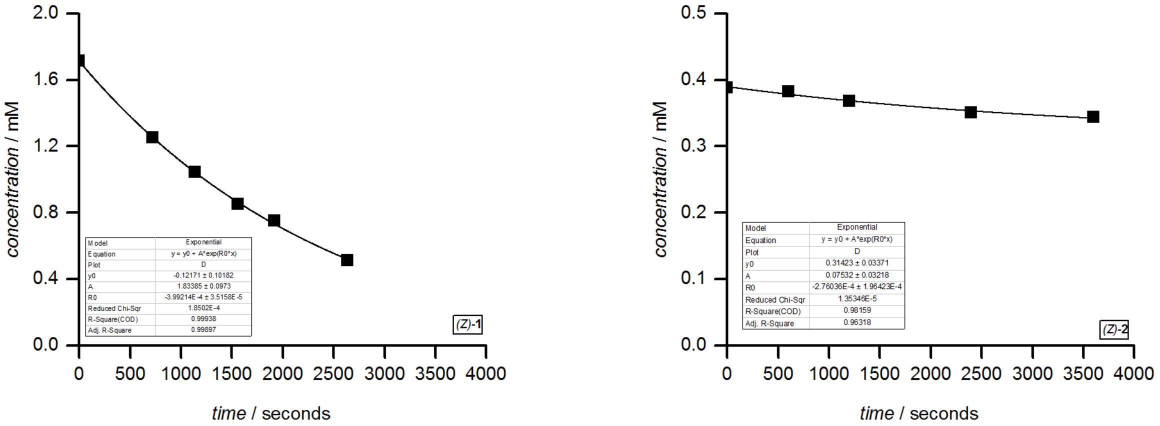

Figure S11. Concentration vs. time graph for the thermal relaxation of *(Z)*-**1** to *(E)*-**1** at 60 °C (left) and *(Z)*-**2** to *(E)*-**2** (right) at 60 °C, as measured by ^{1}H VT-NMR. The rate constant (at 60 °C) for relaxation of *(Z)*-**1** is faster than for that of *(Z)*-**2**.

Side reactions observed during small-molecule studies

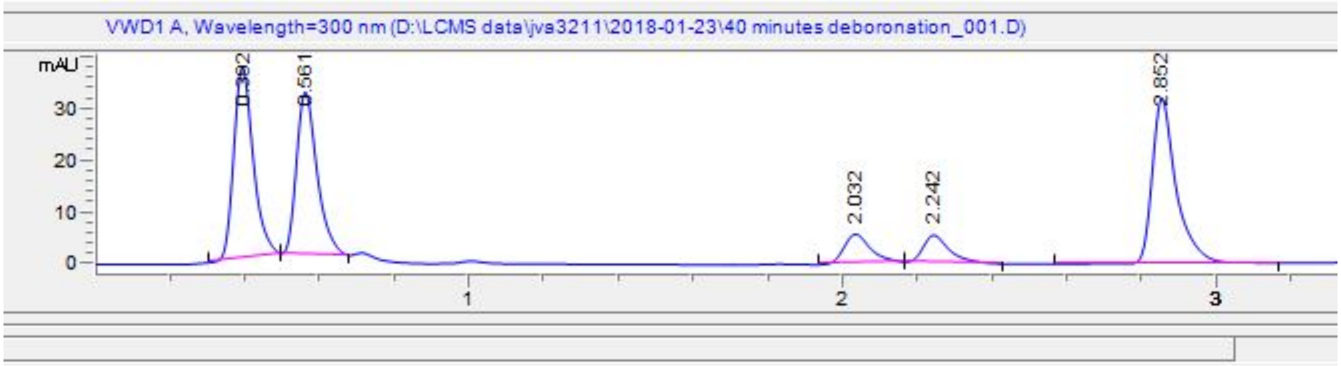

Figure S12. We began to observed protodeboronation of both **1** and **2** after UV irradiation (40 minutes, 400 μM in acetonitrile-H2O). Shown is the LCMS UV-trace after 40 minutes of irradiation of a mixture *E*/*Z* isomers of both **1** and **2** (400 μM total, 1:1 ACN: H2O) which led to the formation of byproduct at time 2.242. The m/z of 241 suggests this peak corresponds to the protodeboronated product of **1** or **2**. Other time point figures are referenced in **Figure S1**. For this reason, we irradiated the samples in ACN prior to mixing with water and observed no deboronation. Given the reversibility of the mechanical properties, we do not believe that the production of boric acid significantly affects the behavior of the gel.

IV. Functionalization of 4-arm PEG

P1 was prepared following a literature procedure.[4]

P2 was synthesized based on an adapted literature procedure.[5] 500 mg of 4-arm PEG amine HCl salt (M_w 5000) was added to a Schlenk flask (50 mL) and melted under high vaccum (3x) to remove excess water. HOBt (0.2 g, 1 mmol), benzotriazol-1-yloxytris(dimethylamino)-phosphonium hexafluorophosphate (0.4 g, 1 mmol), and SI-**2** (0.3 g, 1 mmol) were then added as solids along with a stir bar. The vessel was sealed and backfilled with nitrogen three times. 5 mL of DCM and 5 mL of DMF were then added to solubilize the reagents, leading to a clear red solution. Triethylamine (91 mg, 125 µL, 0.90 mmol) was added to the solution, which was stirred at room temperature for 24 hours. The solvent was removed *in vacuo* and the solution was diluted with deionized water to form an orange precipitate. The solids removed by filtration using a fritted funnel, and the aqueous orange filtrate was subject to dialysis (MWCO = 3.5 kDa) against DI water for 24 hours, changing the dialysate at least 3 times. After dialysis, the sample was lyophilized for 48 hours, yielding 320 mg of an orange powder, **P2**. Polymers should be stored dry in fridge without exposure to light to preserve integrity. Leaving solutions of **P2** in DI H_2O in ambient light for 24 hours leads to protodeboronation, as evinced by proton NMR.

P2-F$_2$ was synthesized using the same procedure as **P2** using SI-**10.** After dialysis this solution was concentration and purified using a spin filter (MWCO = 5KDa) After the reaction this was lyophilized for 48 hours (91 mg yield).

P3 was synthesized by the same procedure as **P2** using SI-**5** (377 mg yield).

P4 was synthesized by same procedure as **P2** using (E)-4-(phenyldiazenyl)benzoic acid (217 mg yield).

Table S1. Characterization of polymers P1-P4 as well as starting material.

**Polymer**	**M_n (SEC), Da**	**M_w (SEC), Da**	**Đ**	**Retention time (min)**	**Amine functionality (%) (^{1}H NMR)[a]**
**4-arm PEG-NH$_2$**	4511	4581	1.02	25.1	100[b]
**P1**	4811	5362	1.11	24.5	99
**P2**	5958	6798	1.14	24.2	100
**P3**	4474	5470	1.22	24.4	63
**P4**	6543	6709	1.02	24.3	100
**P2-F$_2$**	nd	nd	nd	nd	100

[a]4-arm PEG-NH$_2$ was saturated with acetic anhydride in pyridine to functionalize all end groups with acetyl groups. Functionalization of the polymers was compared to that of acetylated 4-arm PEG-NH$_2$ (SI NMRs). [b]Assumed. nd = not determined.

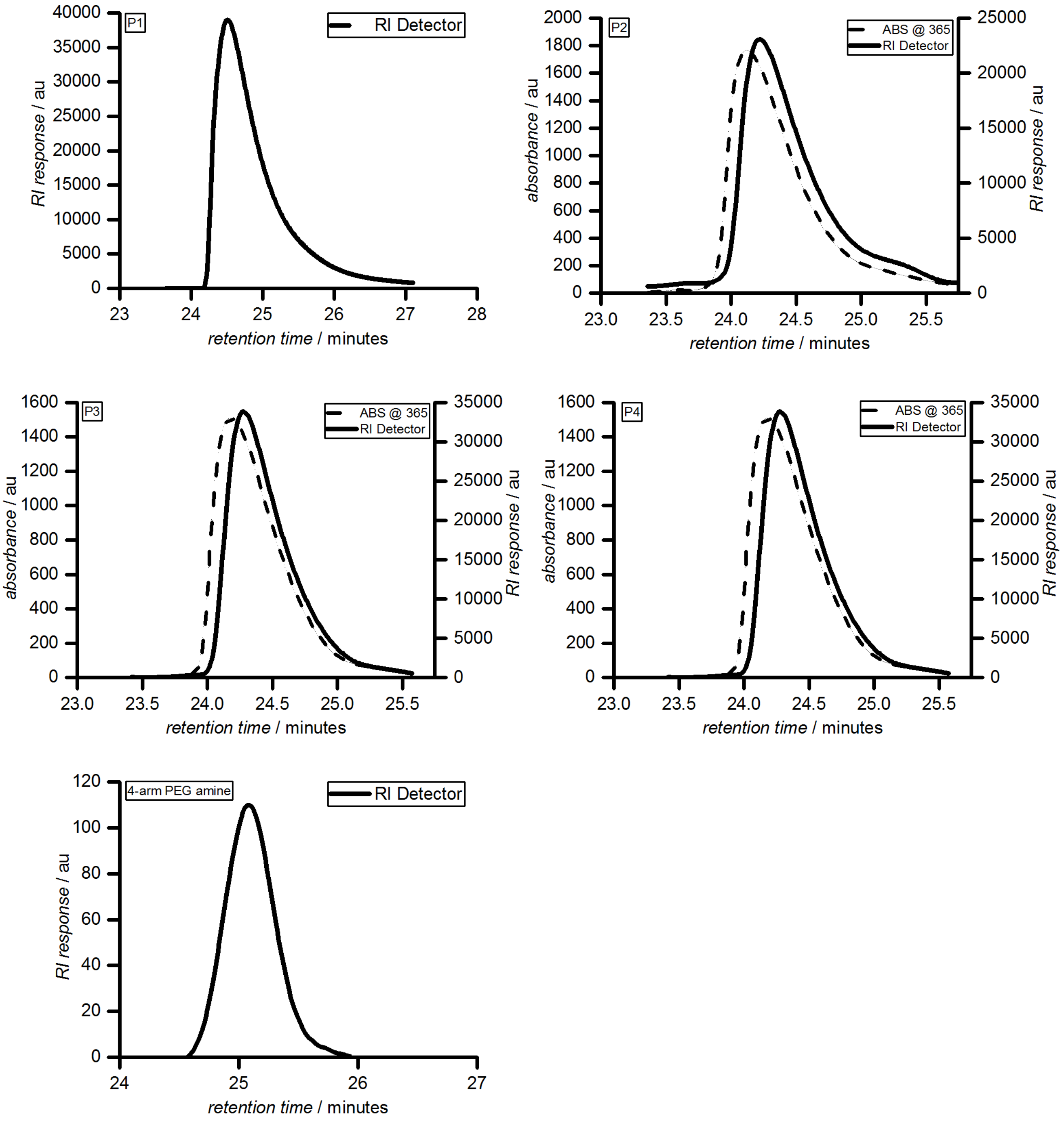

Figure S13. GPC traces of **P1-P4** and starting material 4-arm PEG amine (MW= 5KDa). RI detection (solid line) was overlaid with 365 nm UV-Vis absorbance to qualitatively confirm that azobenzenes were coupled to PEG to yield **P2**-**P4** respectively.

V. Rheology

Mechanical characterization of the prepared hydrogels was performed using an Anton Paar MCR 302 Rheometer with a 25 mm, 5° cone-plate attachment. 10% strain was established to be within the linear viscoelastic regime for all time points tested (Figure S18). Unless noted otherwise, oscillatory strain amplitude sweeps were conducted using a frequency of 25 rad/s and oscillatory frequencies were conducted using 10% strain. Gelation profiles were conducted with 10% strain and a frequency of 25 rad/s. Frequency sweeps were performed at 10% strain, with a frequency range of 300 to 1 rad/s. Data were collected at 25 °C. Gels were prepared by mixing 200 µL of **P1** (10 w/v% in 0.1M PBS, pH 7.5) with **P2**, **P3**, or **P4** (10 w/v% in 0.1M PBS, pH 7.5). The setup for photorheology is shown in the photos section.

An consistent observation from our reversible gelation profiles is that the first stiffening event occurs approximately twice as slowly compared to subsequent cycles. We hypothesize this may be due to (a) a slightly elevated temperature of the glass plate (see photos section) of our photorheology setup after continuous irradiation, (b) to diffusion processes in the macroscopic gel, or (c) the photostationary state after blue irradiation is <100% *E* isomer. While we were unable to control this temperature apart from implementation of a fan, we observed no more than a 3 °C increase in temperature at the sample as measured by as measure by a fluke 62 max IR thermometer.

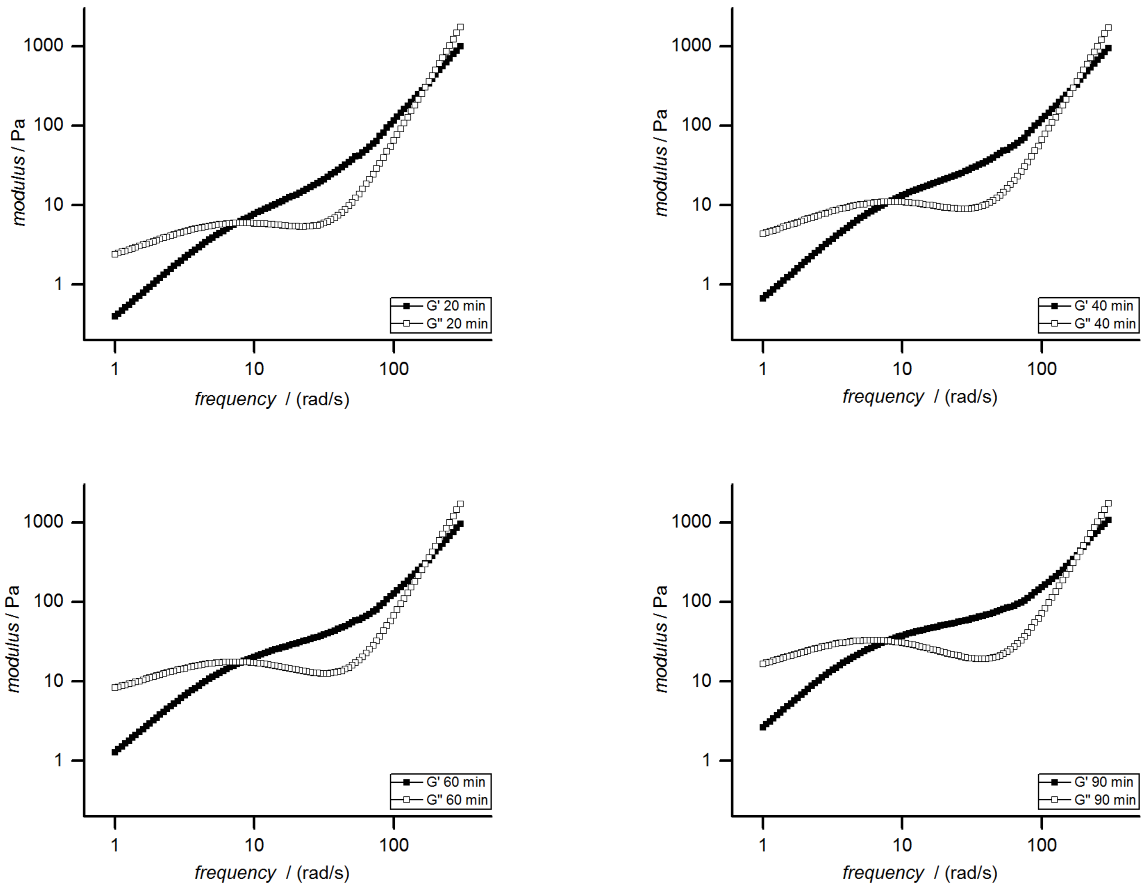

Figure S14. Frequency-dependent oscillatory rheology of hydrogel prepared from **P1** and **P2** (1:1, 10 w/v% in 0.1M PBS, pH 7.5, 10% strain) at irradiation intervals of 20, 40, 60, and 90 minutes.

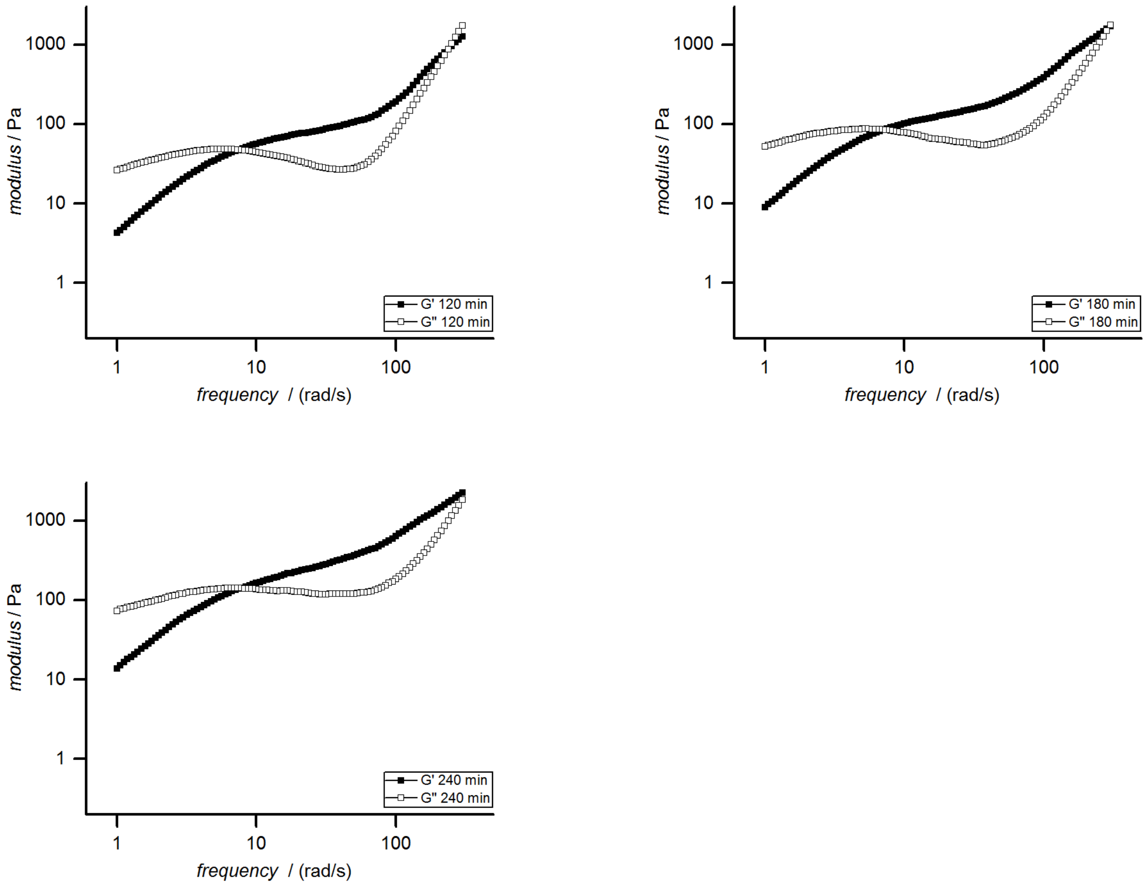

Figure S15. Frequency-dependent oscillatory rheology of hydrogel prepared from **P1** and **P2** (1:1, 10 w/v% in 0.1M PBS, pH 7.5, 10% strain) at irradiation intervals of 120, 180, and 240 minutes.

Table S2. Tabulated data of low frequency crossover points and respective modulus (Pa) of gels prepared from **P1** and **P2** (1:1, 10 w/v% in 0.1M PBS, pH 7.5, 10% strain) at different irradiation intervals.

**time irradiated at 365 nm**	ω_c	**G at** ω_c
**20**	7.49	5.94
**40**	7.98	11
**60**	8.1	17.5
**90**	7.78	32.2
**120**	7.45	47.5
**180**	7.11	84.3
**240**	7.75	141

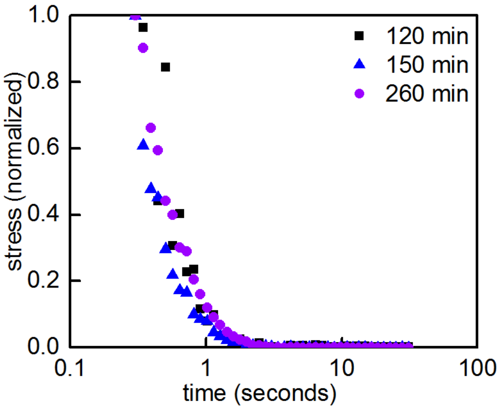

Figure S16. Stress relaxation profile of gel prepared from **P1** and **P2** (1:1, 10 w/v% in 0.1M PBS, pH 7.5, 10% strain) after 120, 150, and 260 minutes of UV irradiation.

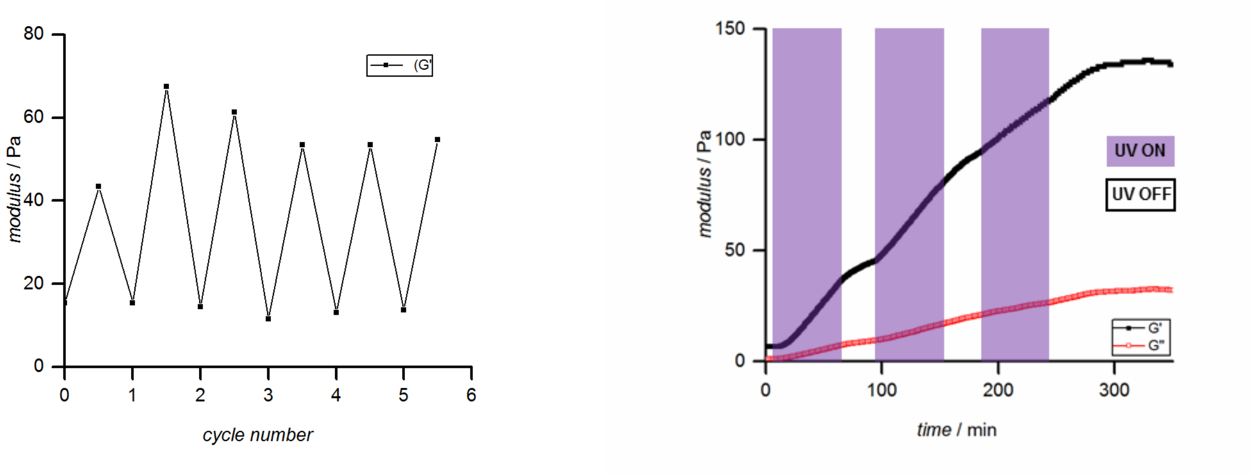

Figure S17. Left: Short-interval cycling data with gels fabricated from **P1** and **P2** (1:1, 10 w/v% in 0.1M PBS, pH 7.5, 10% strain, 25 rad/s). Each cycle represents 30 minutes of gelation with 365 nm light followed by 2 minutes of 470 nm blue light. Right: Attempted temporal control experiment of gel prepared from **P1** and **P2** (1:1, 10 w/v% in 0.1M PBS, pH 7.5, 10% strain, 25 rad/s).

There is an induction period prior to the beginning of gelation when the UV light is turned on, and when UV light is removed, the gel continues to stiffen at a slower rate. The induction period may be due to the optical density of the macroscopic gel: some time is required for enough light to pass through the gel to photoswitch enough end groups, and cause a macroscopic response. We hypothesize that the gel continues to stiffen after the light is turned off because of hindered diffusion of the polymer chains through the macroscopic sample, and/or because isomerization and crosslinking are separate steps. The sample is not homogeneous because of its optical density, and more *Z* azobenzenes exist closer to the bottom of the sample (where light enters) than at the top. When the light is turned off, polymer chains continue to diffuse and the sample becomes more homogeneous, which appears as stiffening. Alternatively, the thermal esterification of the boronic acids to stiffen the gel occurs more slowly than the isomerization photoreaction, so when the light is turned off, the isomerization stops but the crosslinking continues some time. We will test these hypotheses in future work.

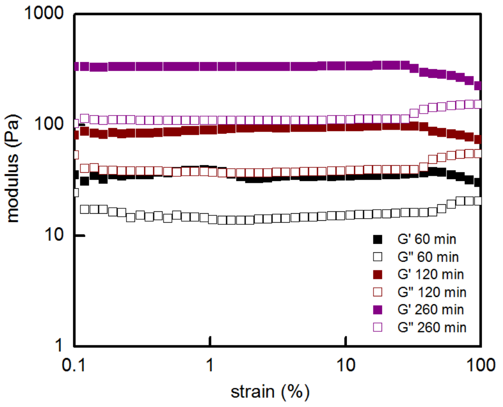

Figure S18. Amplitude sweeps of a mixture of **P1** and **P2** (1:1, 10 w/v% in 0.1M PBS, pH 7.5, 25 rad/s) after different intervals of irradiation with 365 nm UV light. 10% strain is within the linear viscoelastic regime.

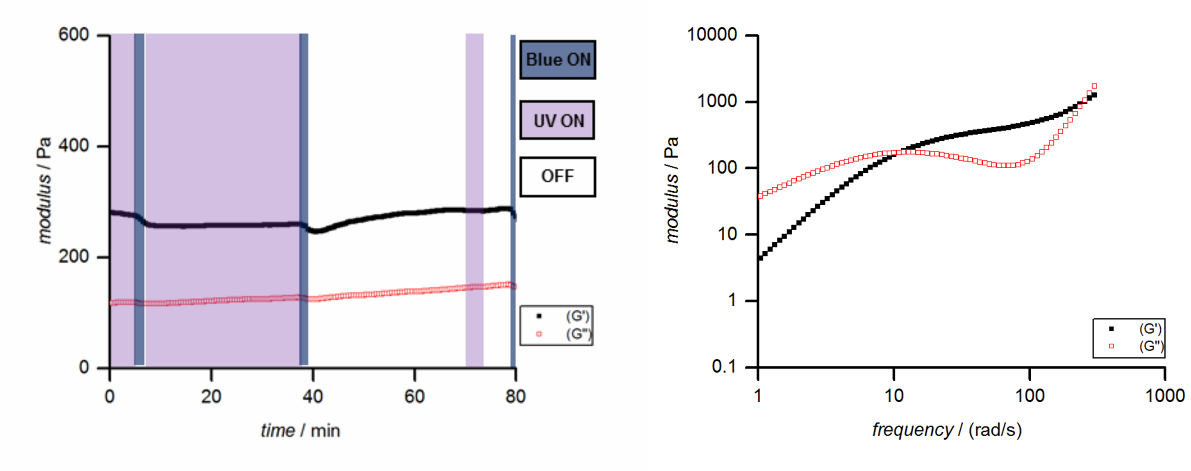

Figure S19. Left: UV and blue cycle of a mixture of **P1** and **P3** (1:1, 10 w/v% in 0.1M PBS, pH 7.5, 10% strain, 25 rad/s). From this data, we believe that both UV and blue light are decreasing storage modulus by secondary thermal affect, where the change in G' is no more than 16 Pa using the same irradiation method as for the photocontrolled system. A hand-held IR thermometer measured a maximum 3 °C temperature increase when the blue light was turned on. Right: Frequency Sweep of sample after irradiation cycle (10% strain, 300 to 0.1 rad/s).

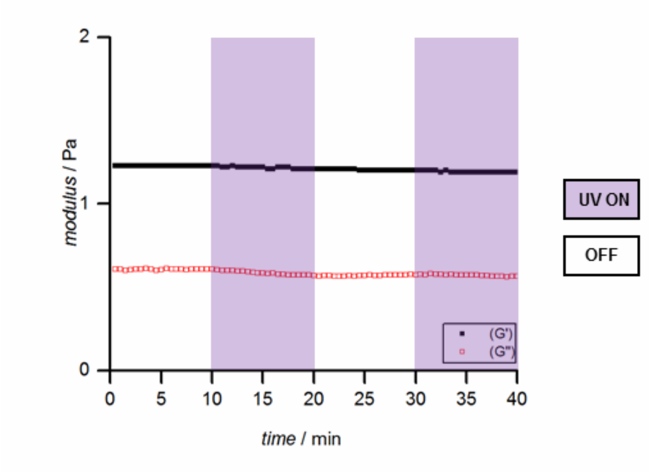

Figure S20. UV on- UV off cycles of a mixture of **P1** and **P4** (1:1, 10 w/v% in 0.1M PBS, pH 9, 10% strain, 10 rad/s)

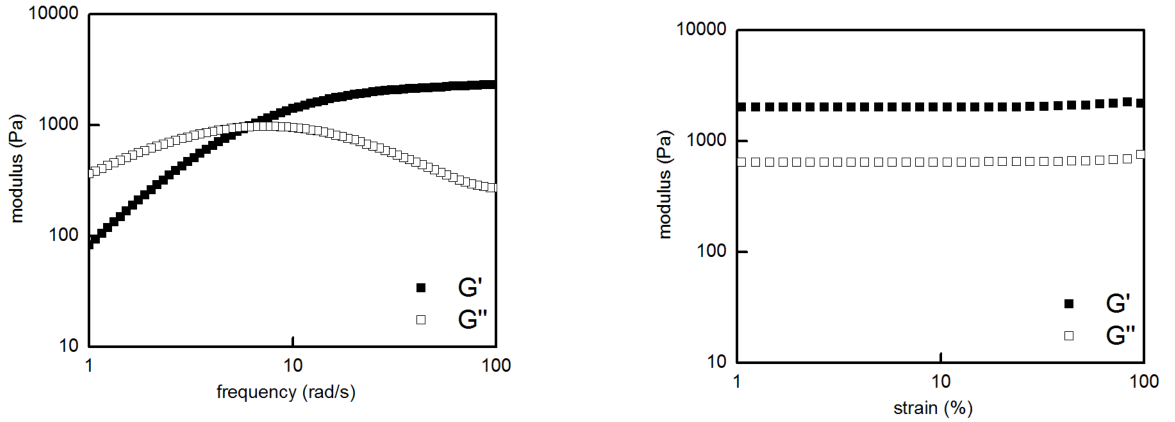

Figure S21. Left: frequency sweep of **P1** and **P2-F$_2$** after 45 minutes of gelation with green LEDs (1:1, 10 w/v% in 0.1M PBS, pH 7.5, 10% strain, 25 rad/s). Right: amplitude sweep of the same sample (25 rad/s).

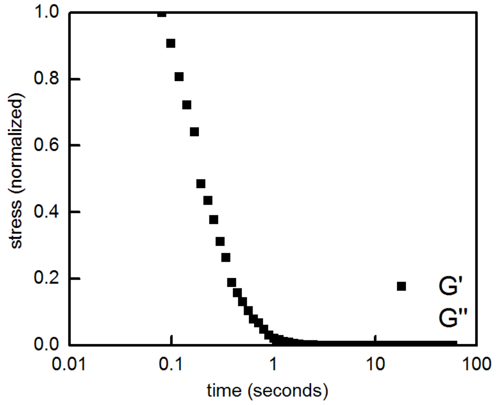

Figure S22. stress relaxation experiment of **P1** and **P2-F$_2$** after 45 minutes of gelation with green LEDs (1:1, 10 w/v% in 0.1M PBS, pH 7.5, 10% strain).

VI. Absorption Spectra

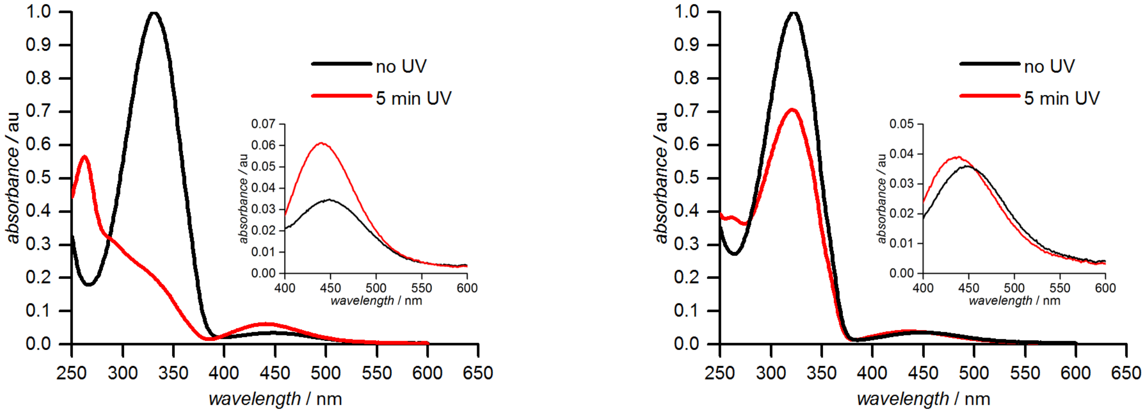

Figure S23. Left: Normalized UV-Vis absorbance spectra of *(E)*-**1** (8 µM in acetonitrile) before (black) and after (red) five minutes of irradiation. Right: Normalized UV-Vis of *(E)*-**2** (8 µM in acetonitrile) before (black) and after (red) five minutes of irradiation. The resolution of spectra before and after irradiation suggest why the ratio of *Z*/*E* for compound **1** is higher than that of **2** in the kinetic experiments.

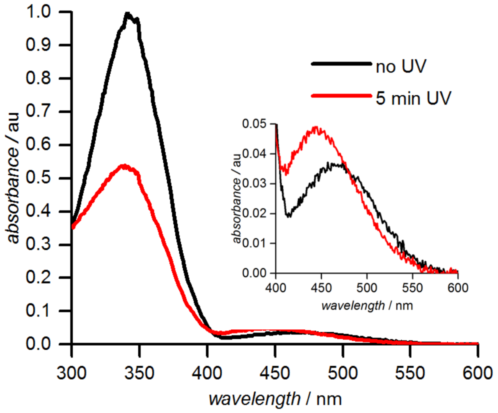

Figure S24. Normalized UV-Vis of *(E)*-SI-**8** (70 µM in acetonitrile) before (black) and after (red) fifteen minutes of green LED irradiation.

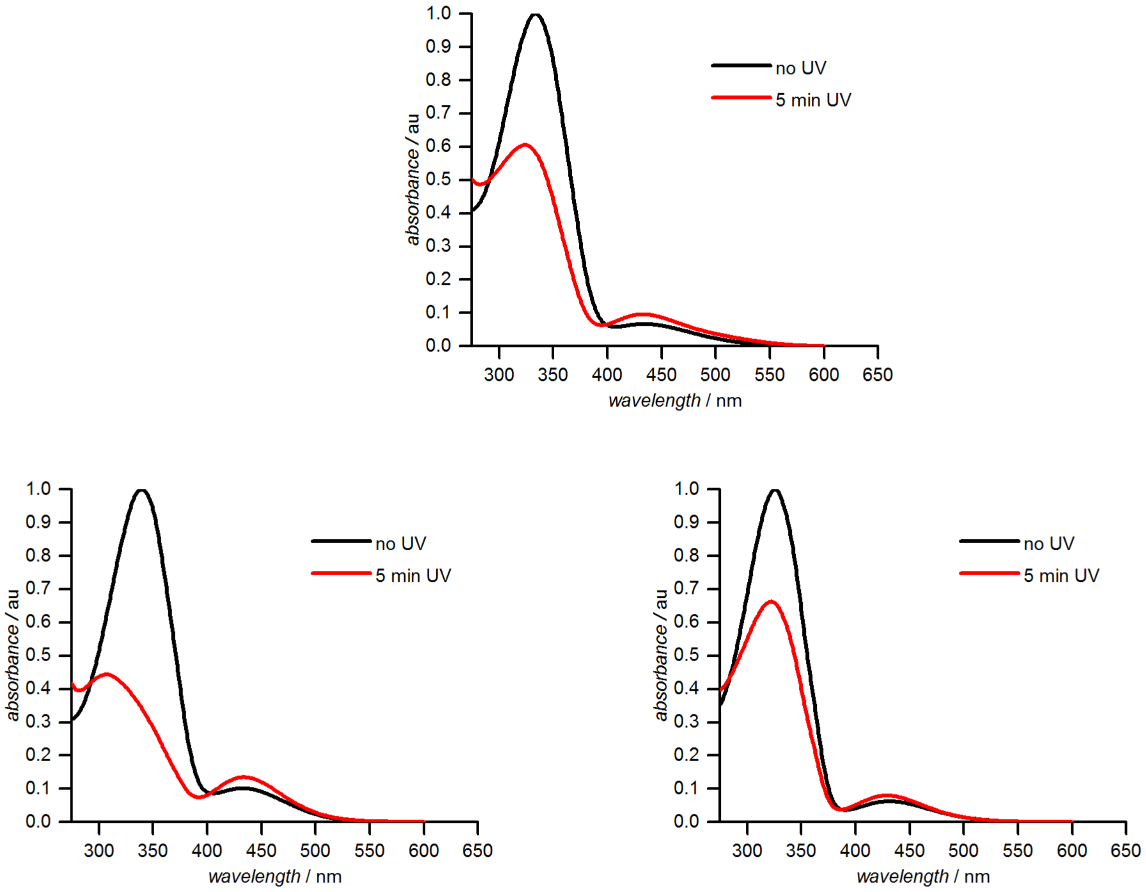

Figure S25. Normalized UV-Vis absorbance spectra of mixture of polymers in phosphate buffered saline (1:1, 0.1 w/v% pH 10) before (solid) and after (dash) 10 minutes of 365 nm irradiation. Top: **P1** & **P2**, bottom left, **P1** & **P3**, bottom right, **P1** & **P4**. Samples were diluted so that UV-Vis detector saturation did not occur. In all cases, the UV-Vis spectra indicate that *E*-to-*Z* isomerization is occurring in the polymers.

VII. NMR & IR Spectra

It should be noted that carbons bound to boron do not appear in the ^{13}C NMR spectrum. Small-molecule spectra show ^{1}H, ^{13}C, ^{11}B, and IR spectra respectively.

2

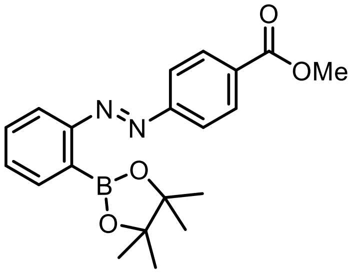

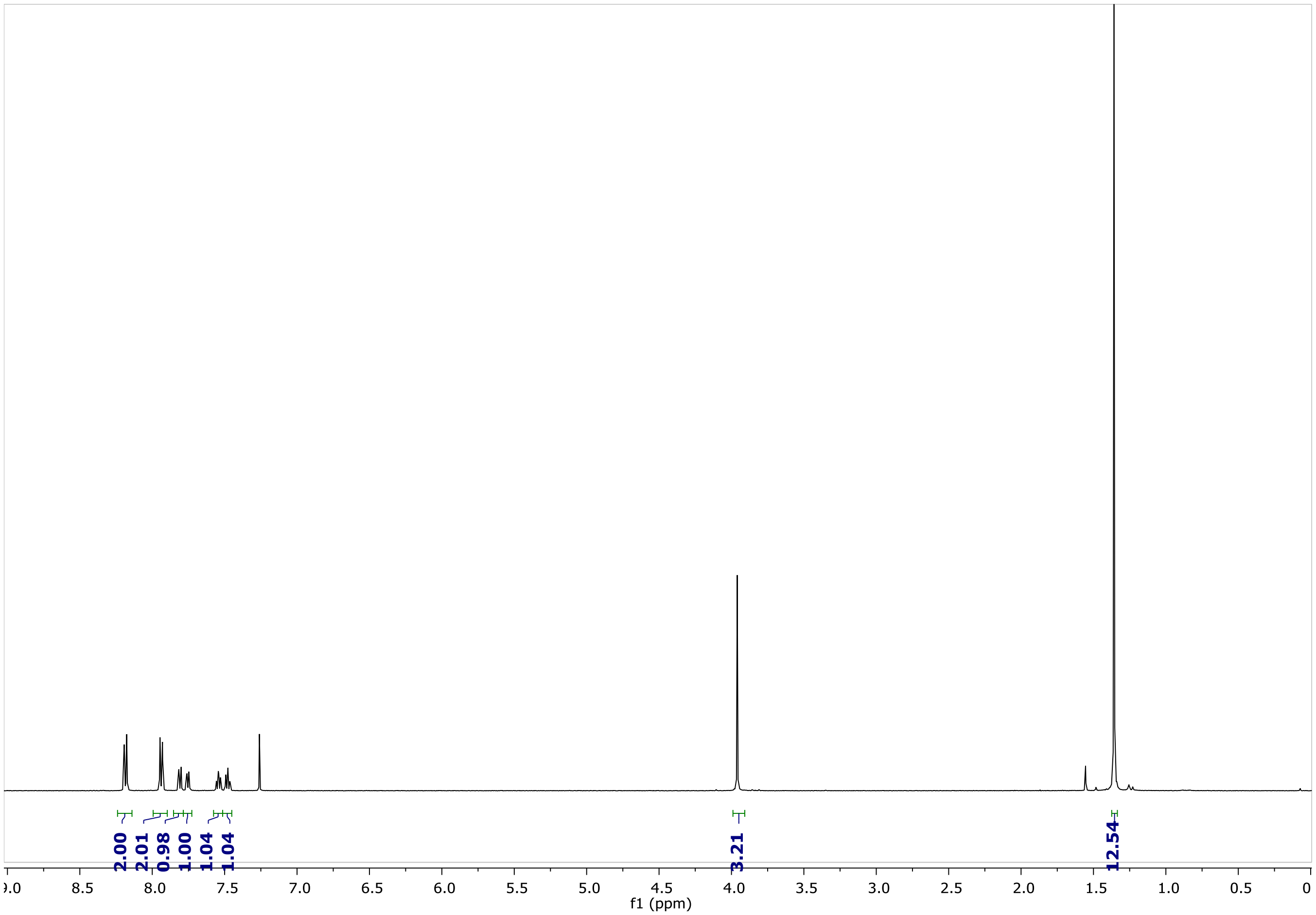

^{1}H NMR, $CDCl_3$

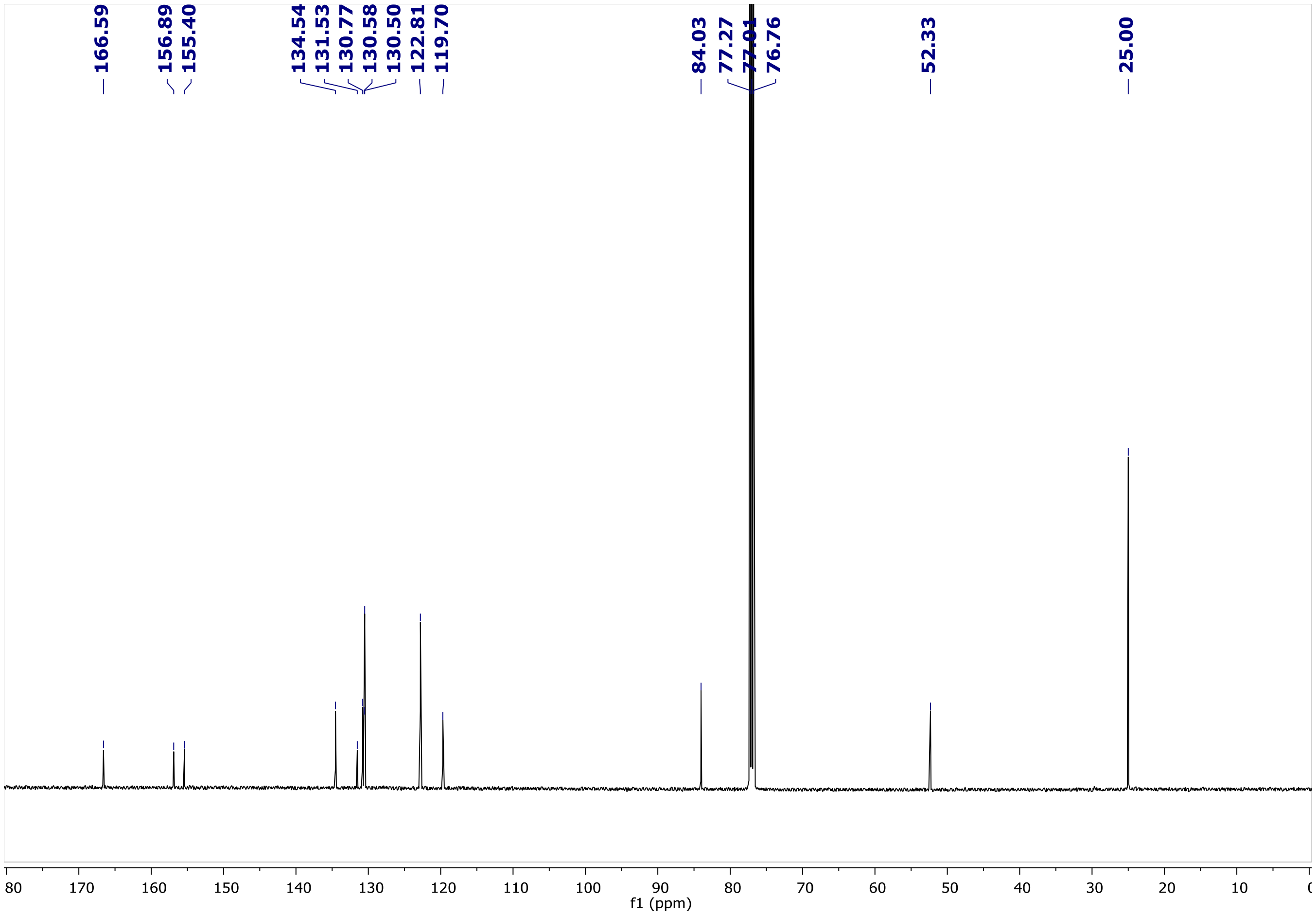

^{13}C NMR, $CDCl_3$

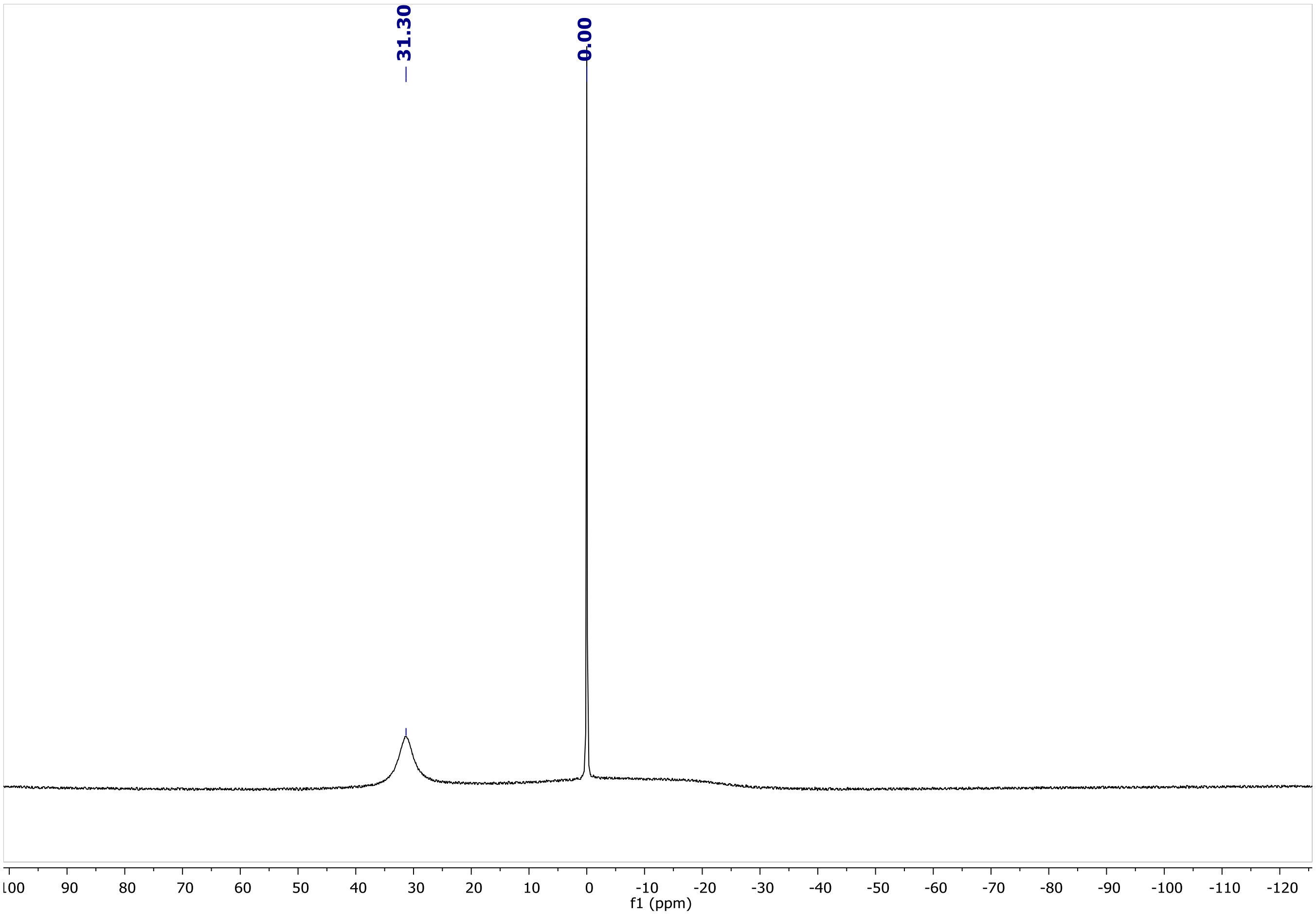

^{11}B NMR, $CDCl_3$

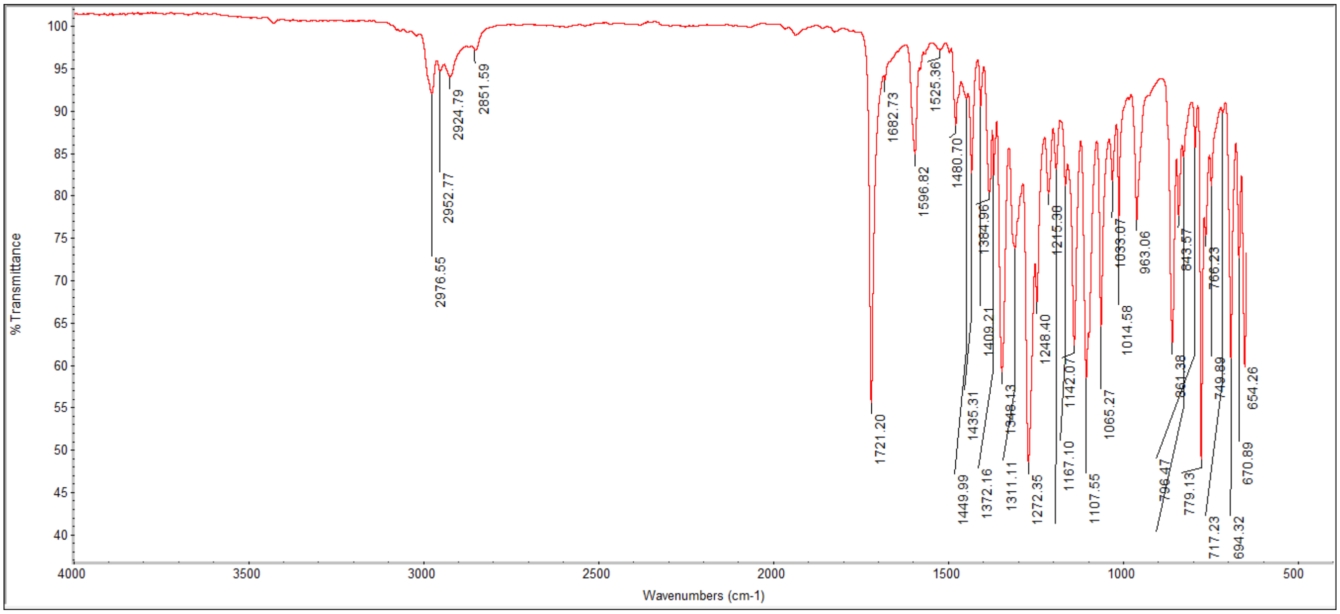

FTIR

1

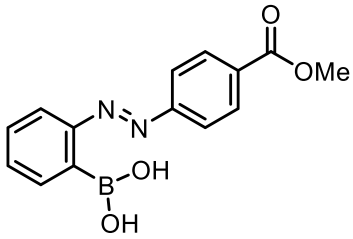

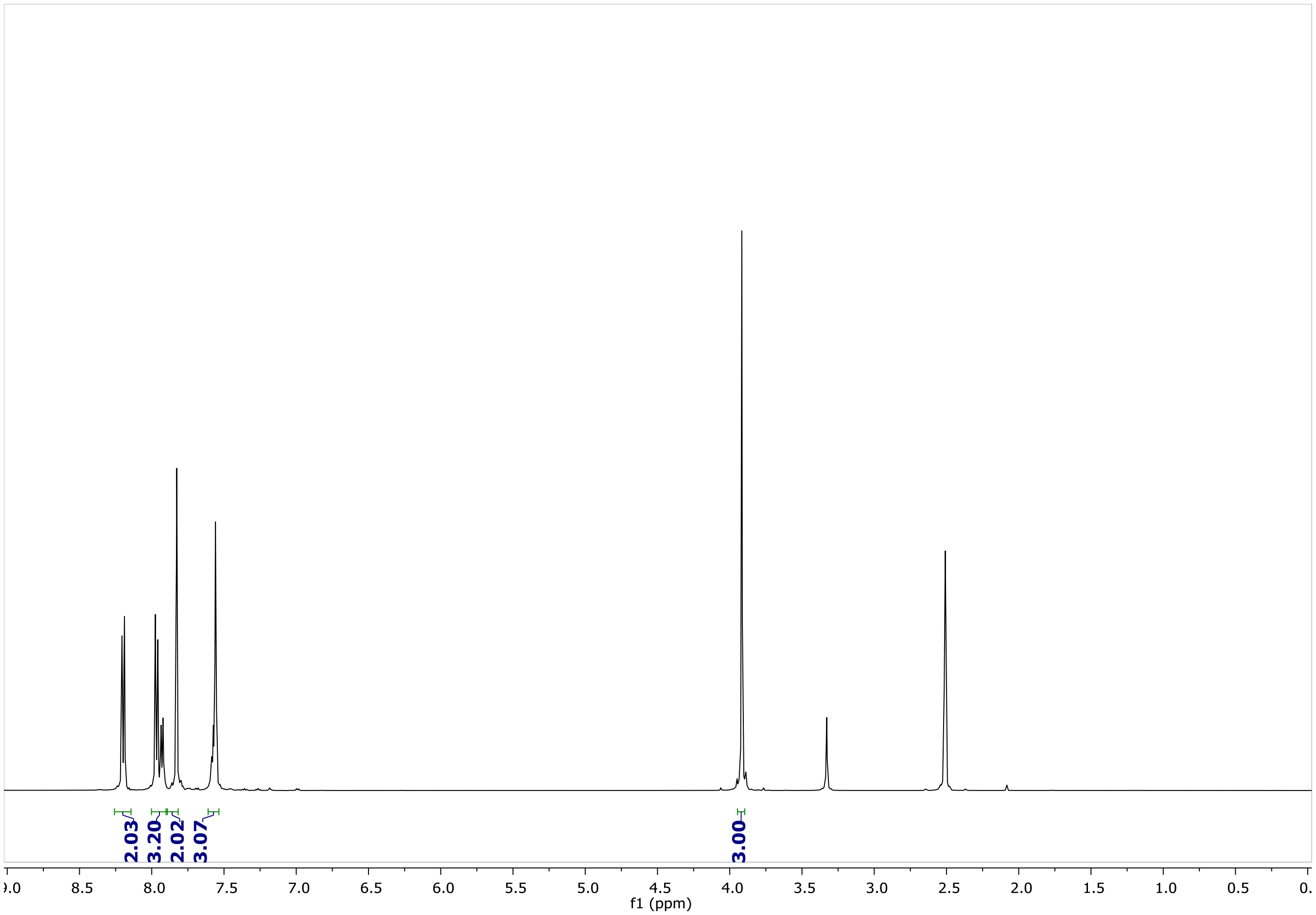

^{1}H NMR, DMSO-d_6

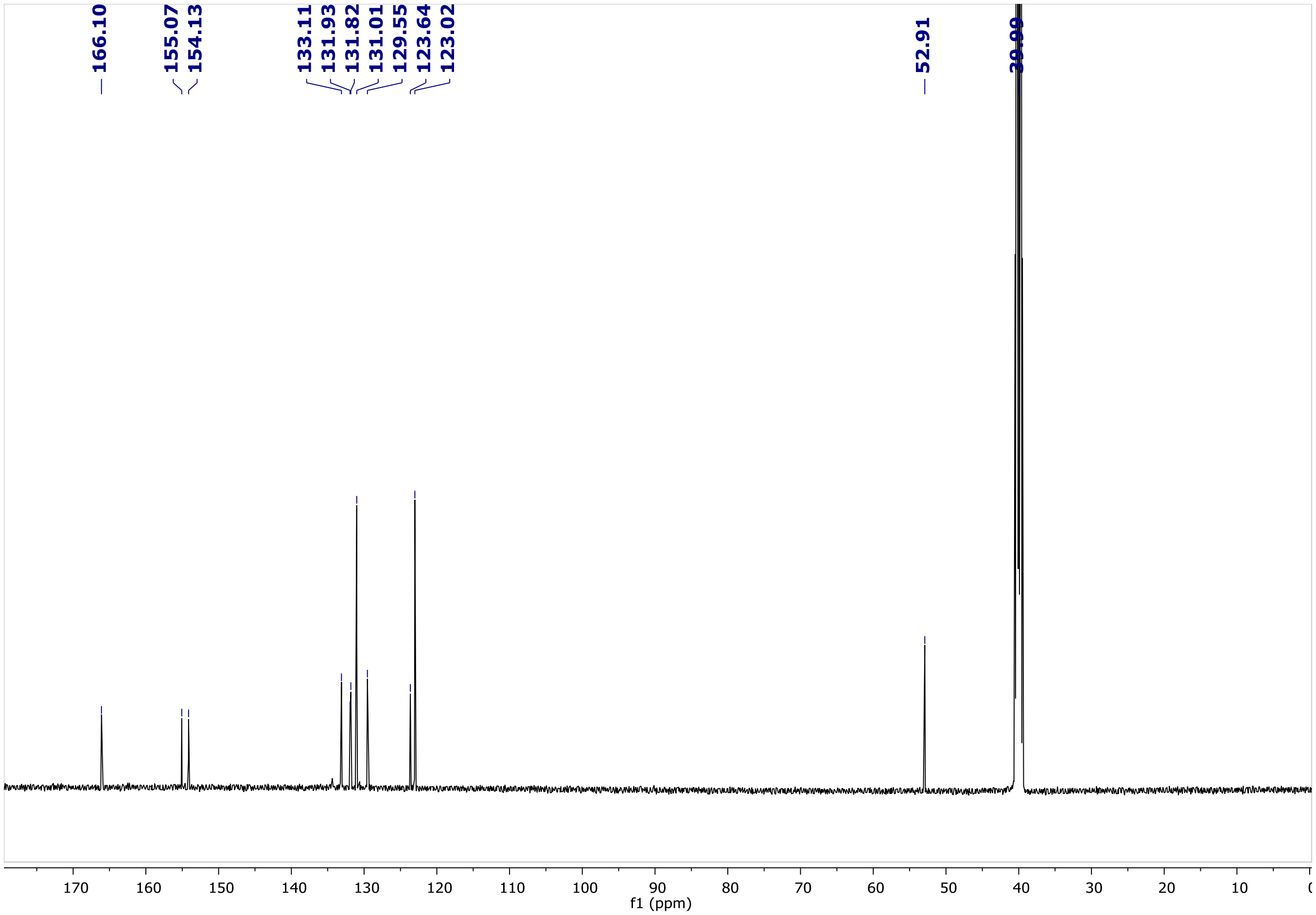

^{13}C NMR, DMSO-d_6

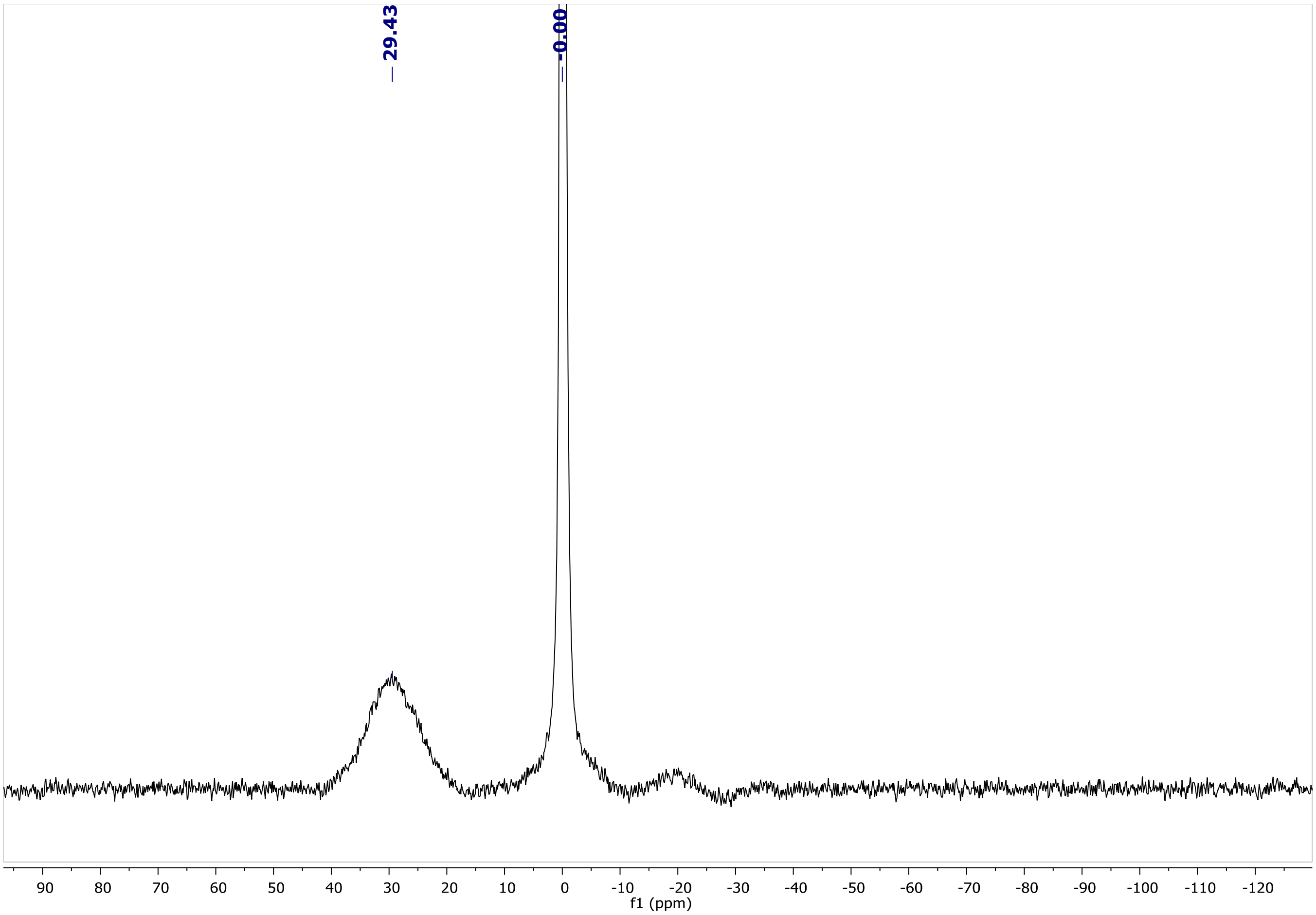

^{11}B NMR, DMSO-d_6

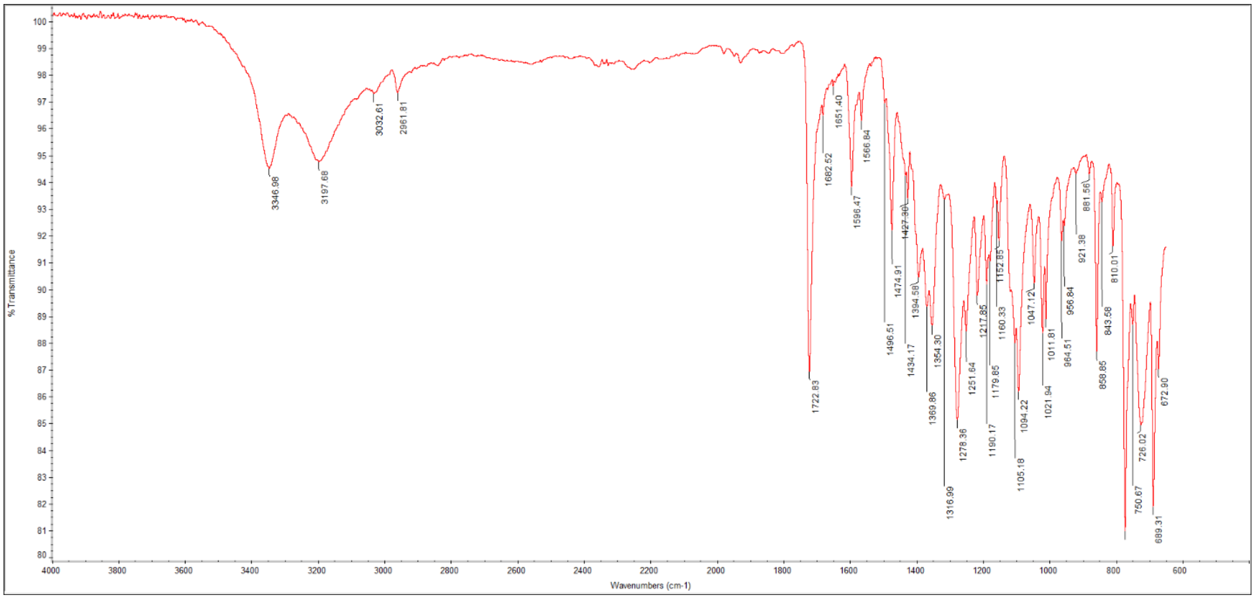

FTIR

SI-**2**

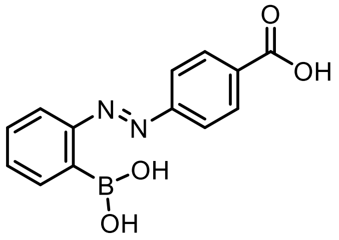

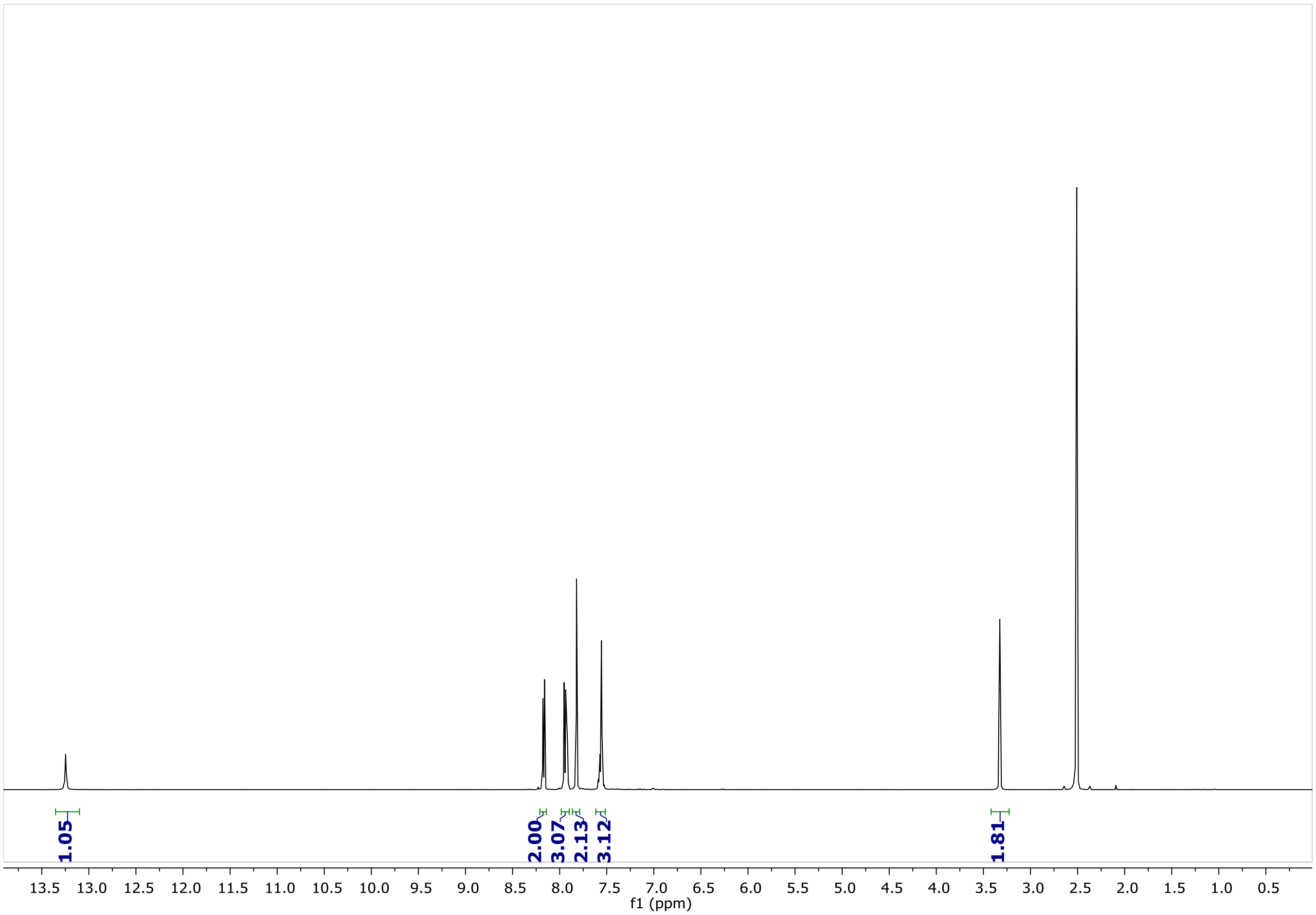

^{1}H NMR, DMSO-d_6

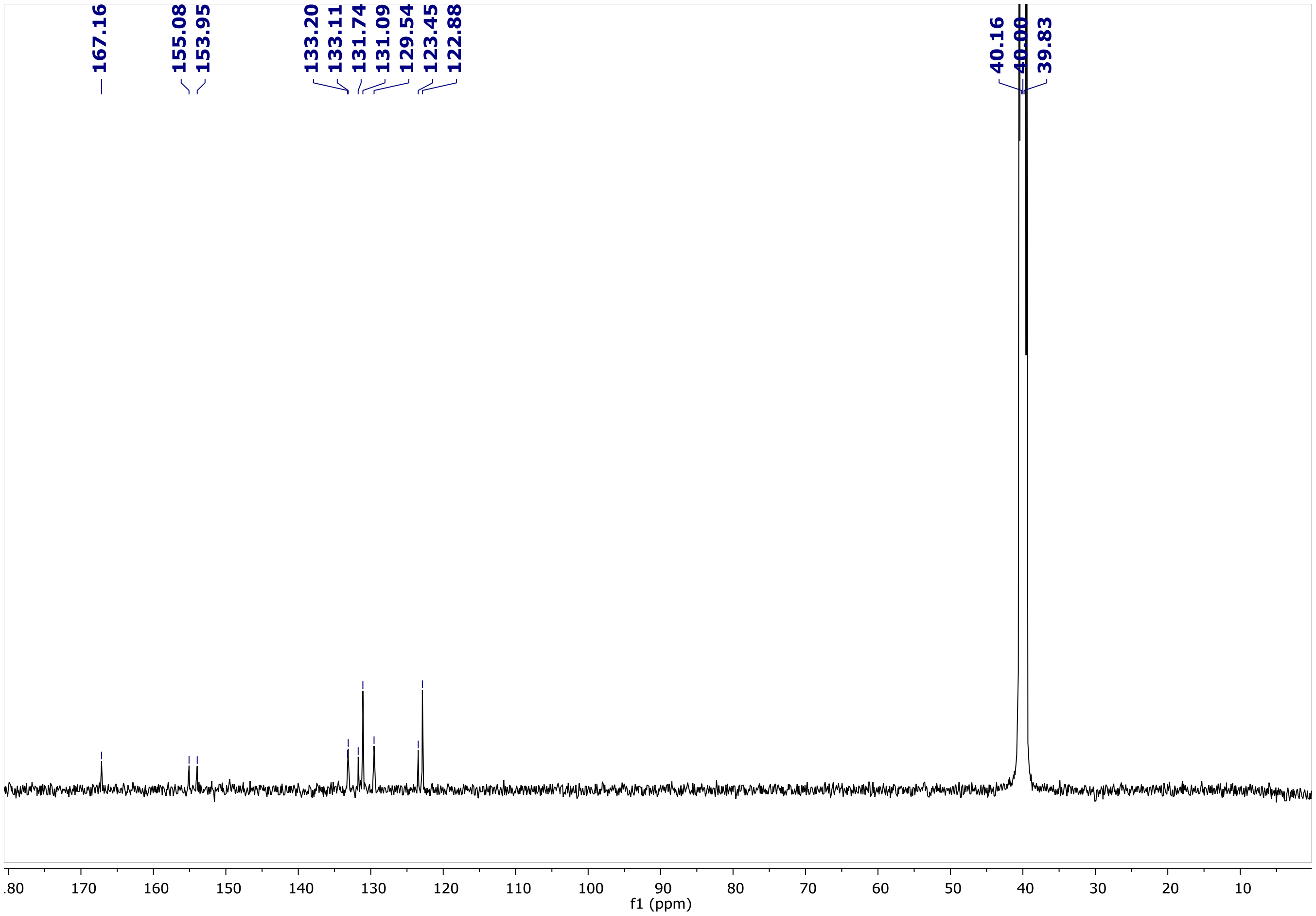

^{13}C NMR, DMSO-d_6

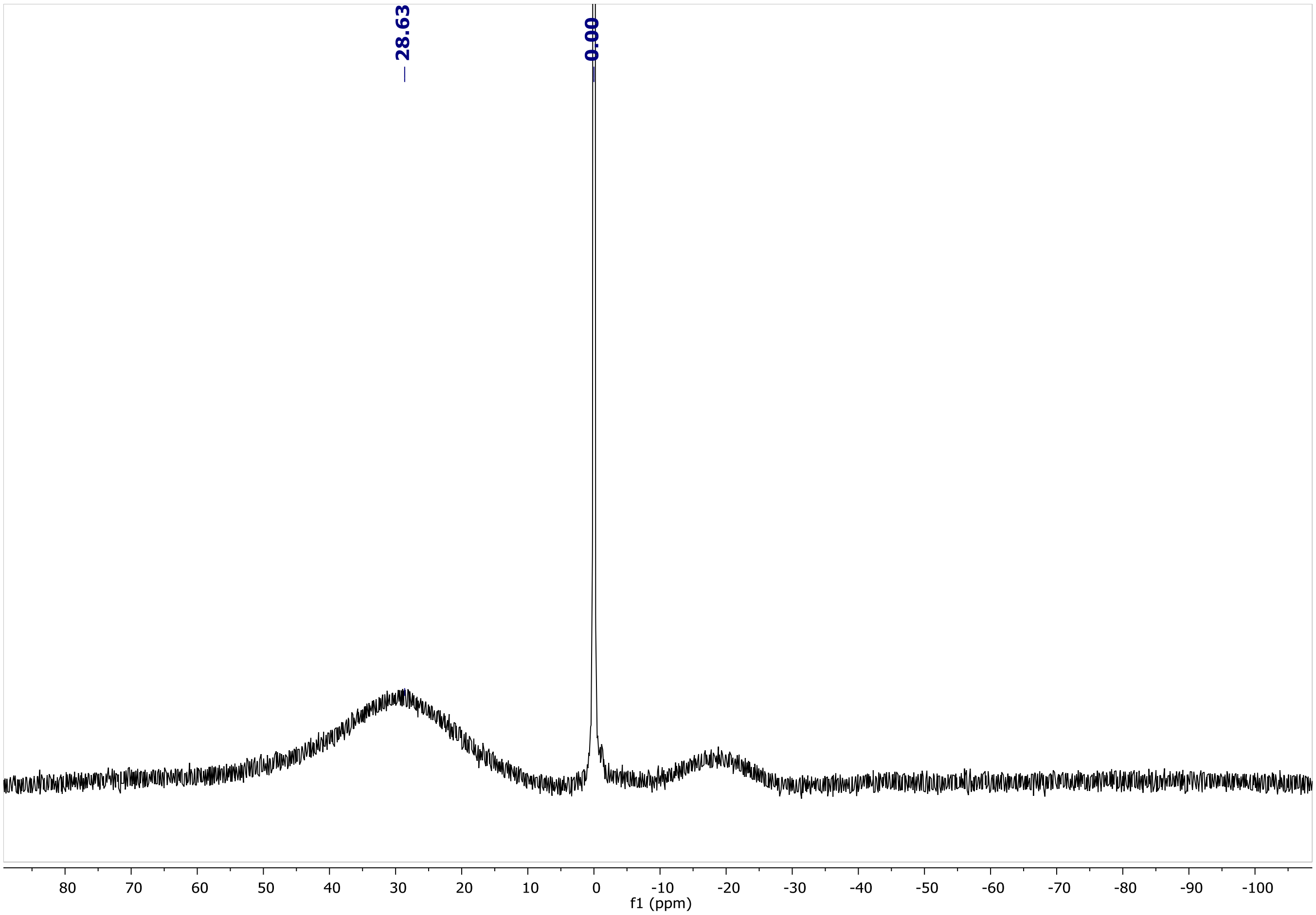

^{11}B NMR, DMSO-d_6

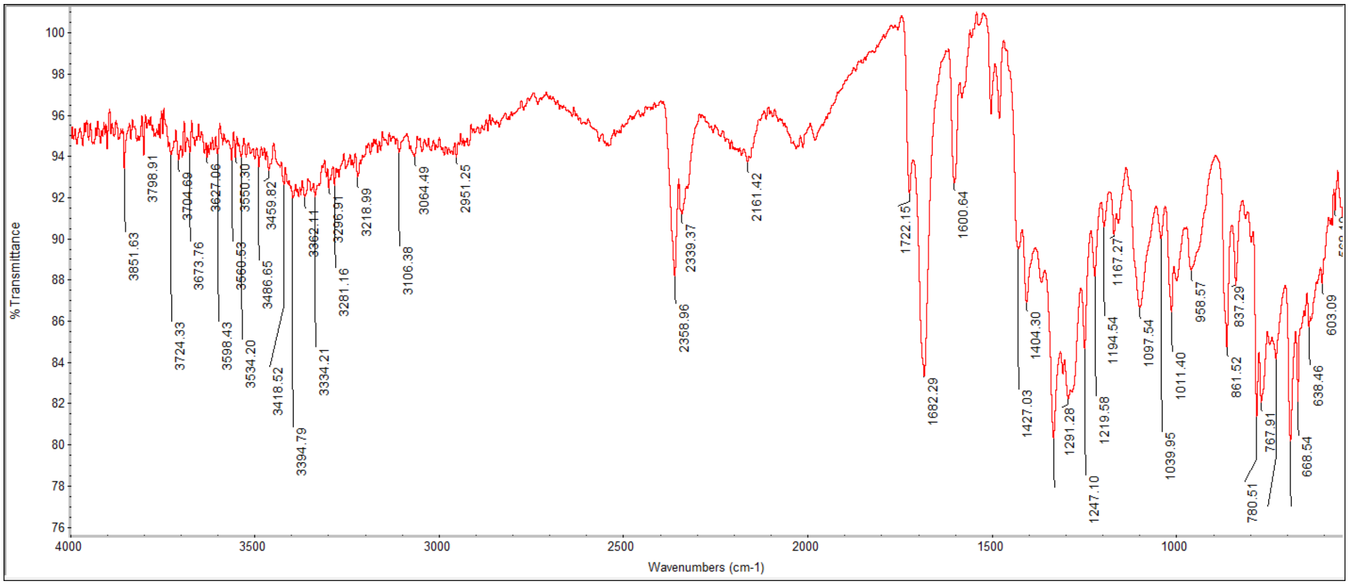

FTIR

SI-**3**

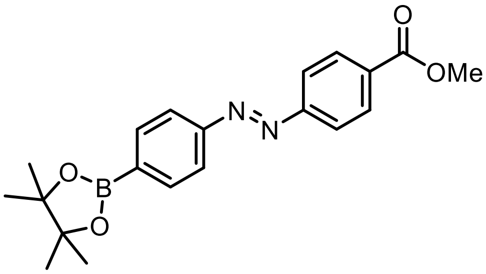

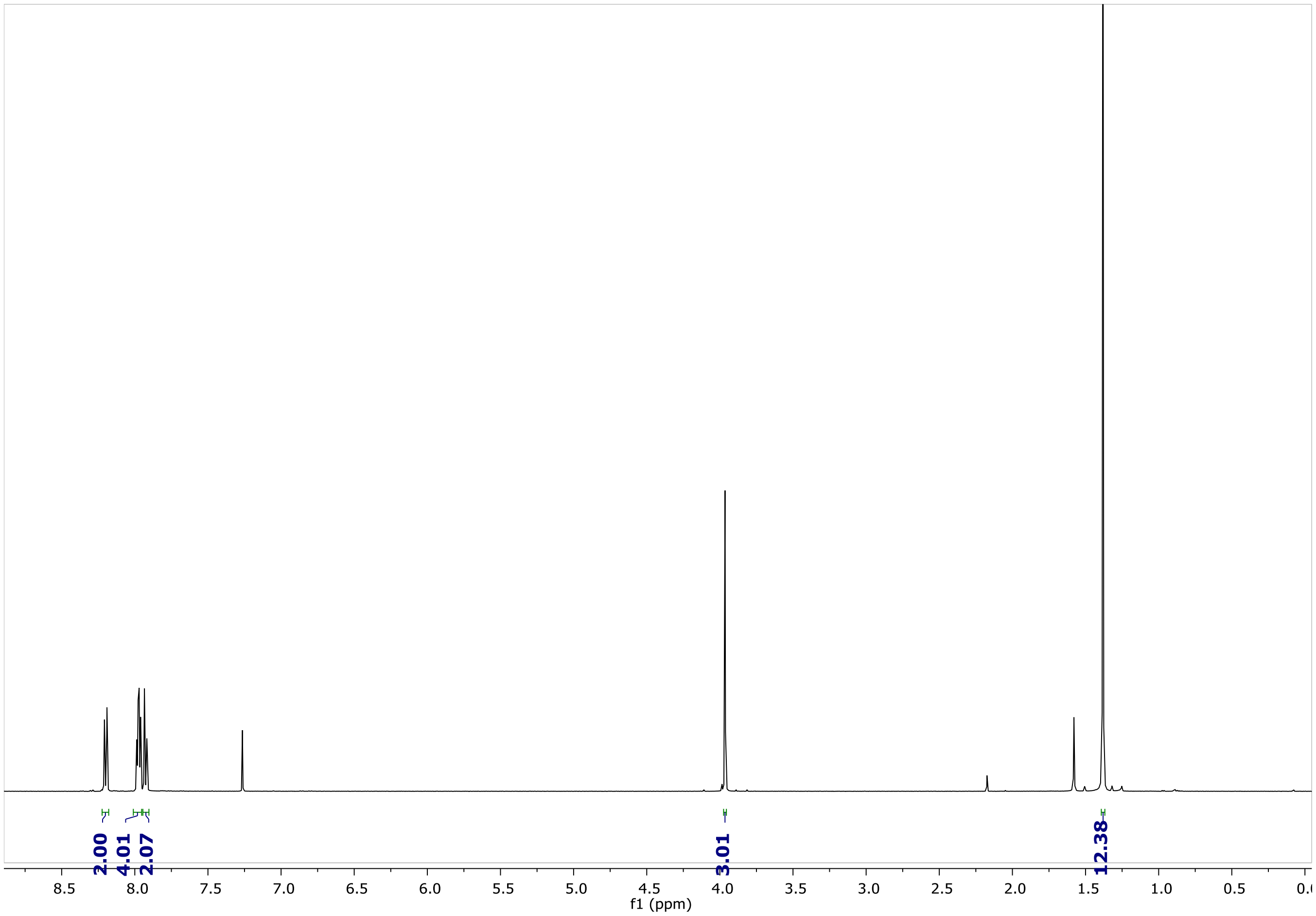

^{1}H NMR, $CDCl_3$

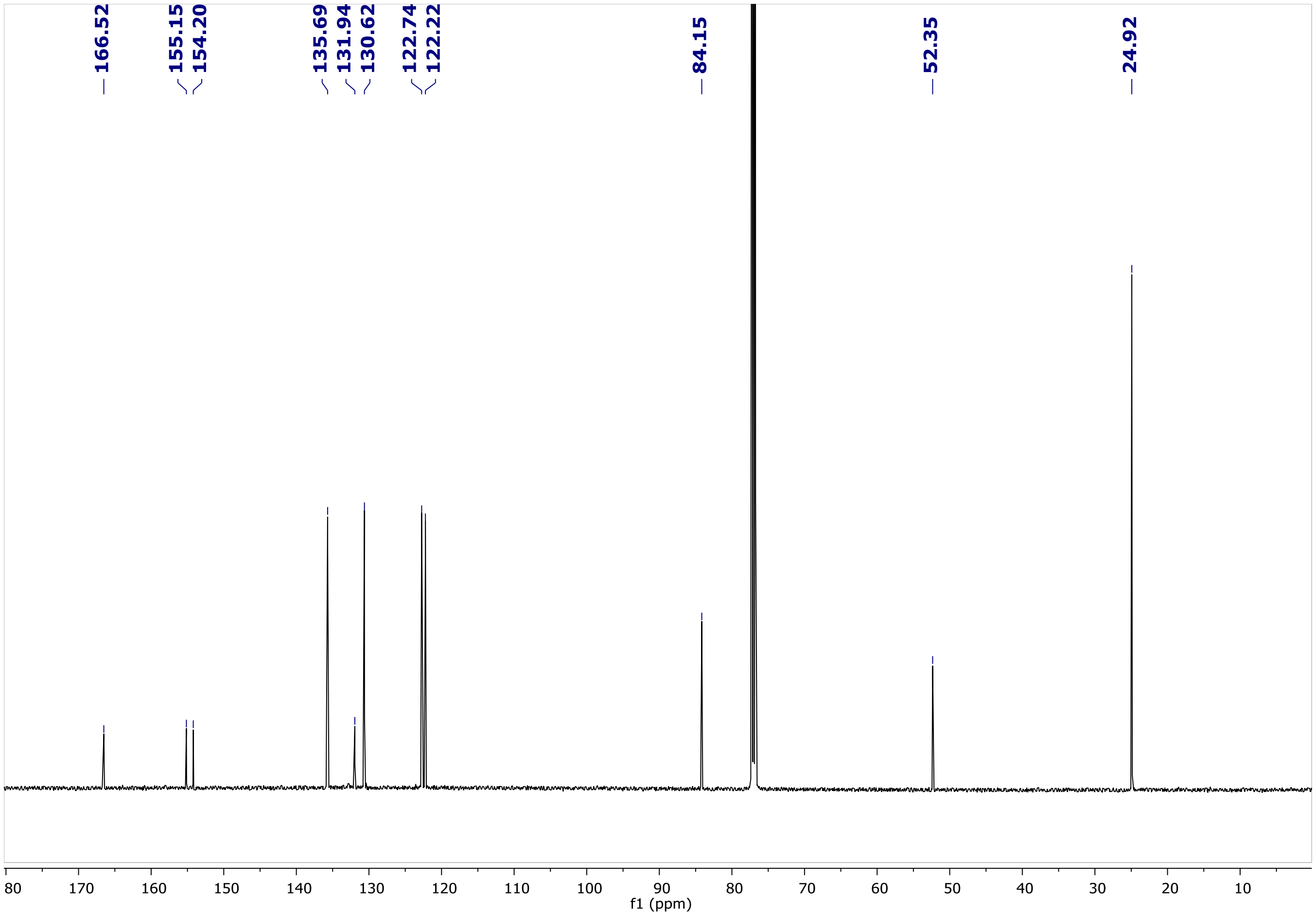

^{13}C NMR, $CDCl_3$

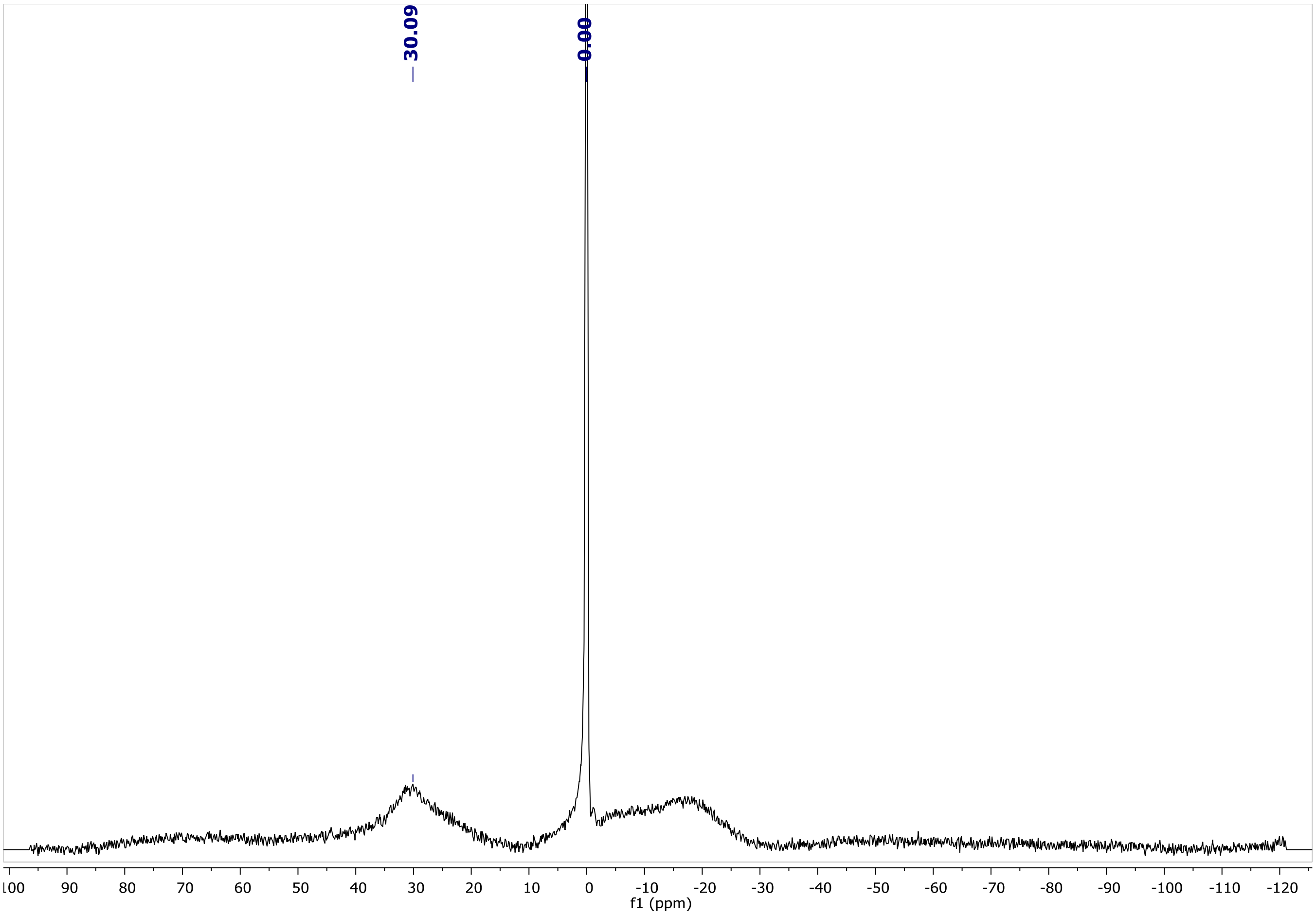

^{11}B NMR, $CDCl_3$

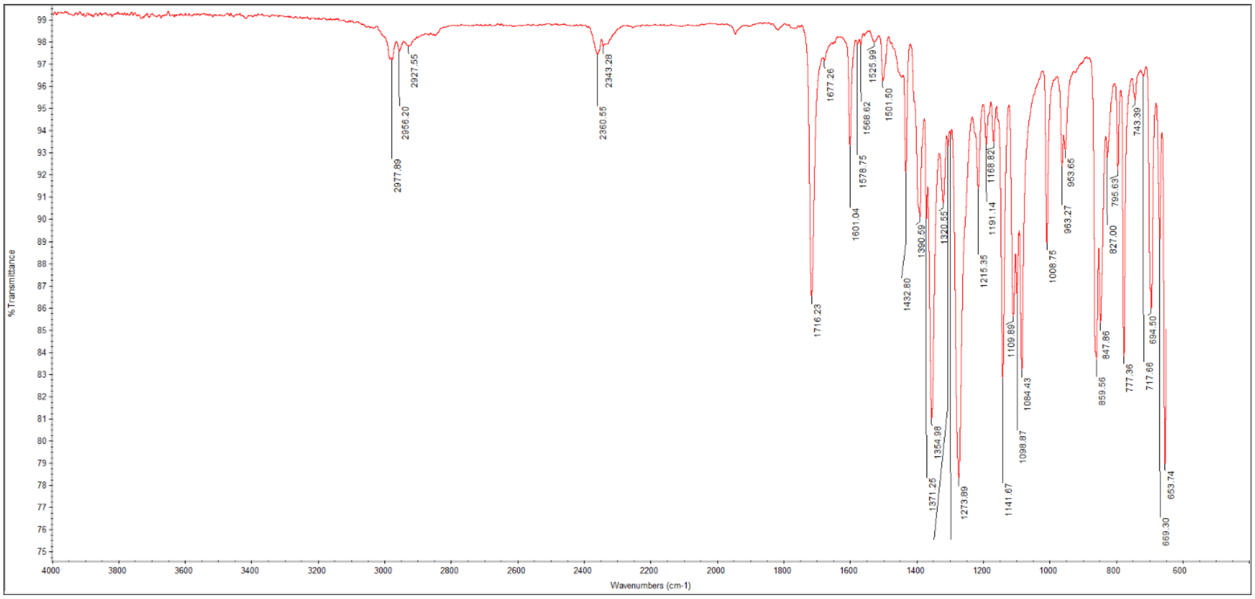

FTIR

SI-**4**

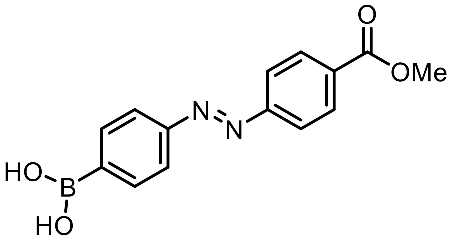

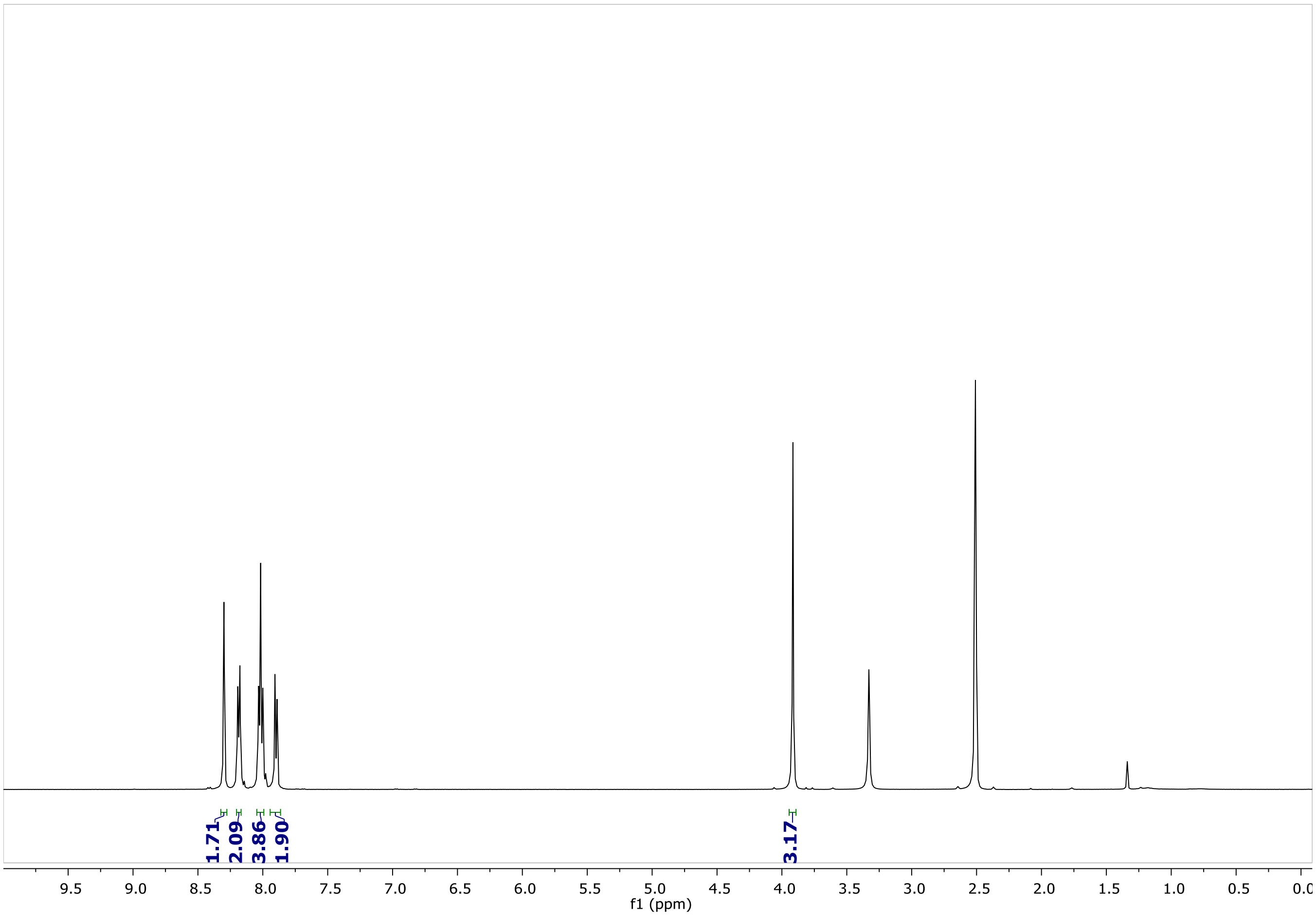

^{1}H NMR, DMSO-d_6

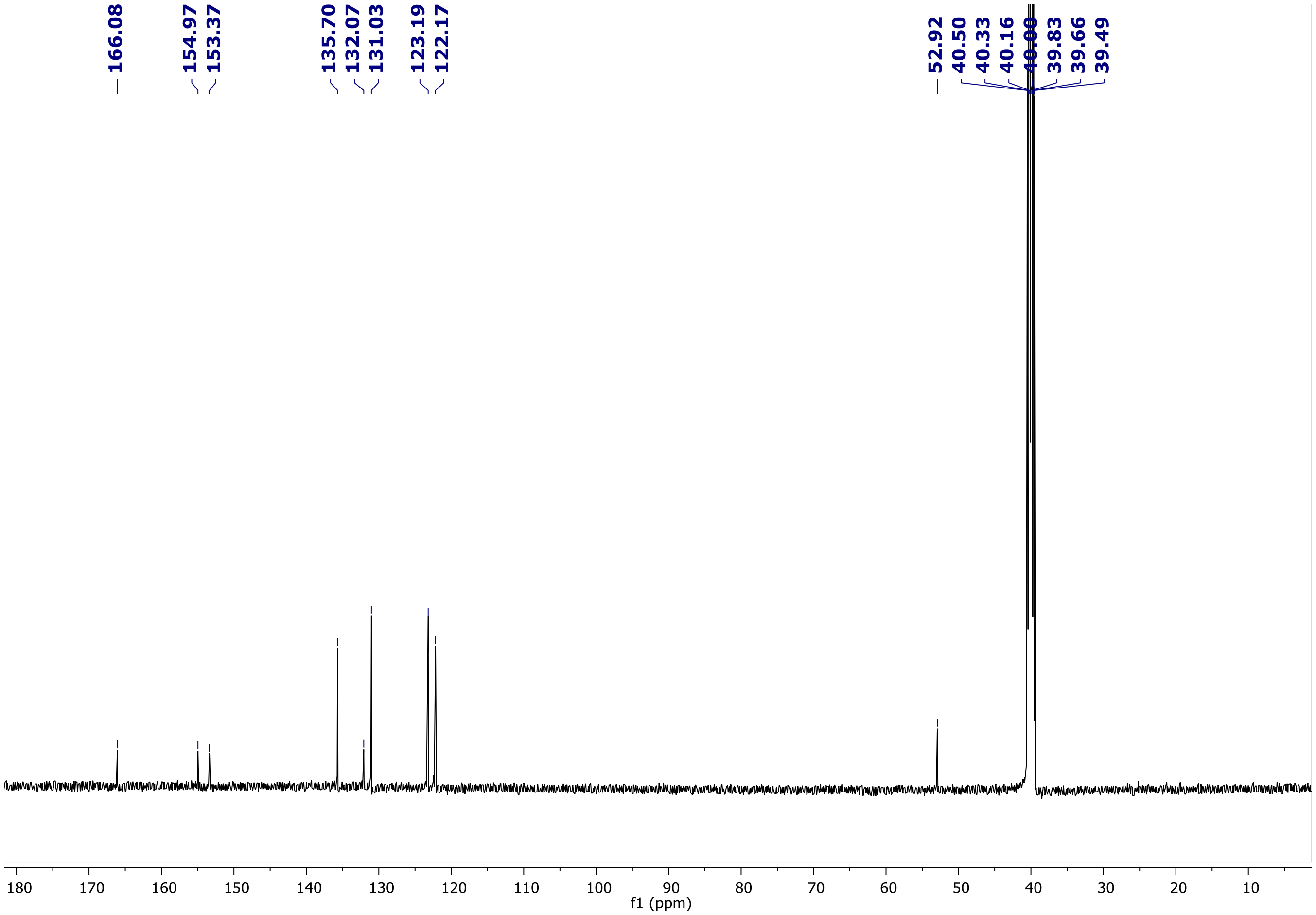

^{13}C NMR, DMSO-d_6

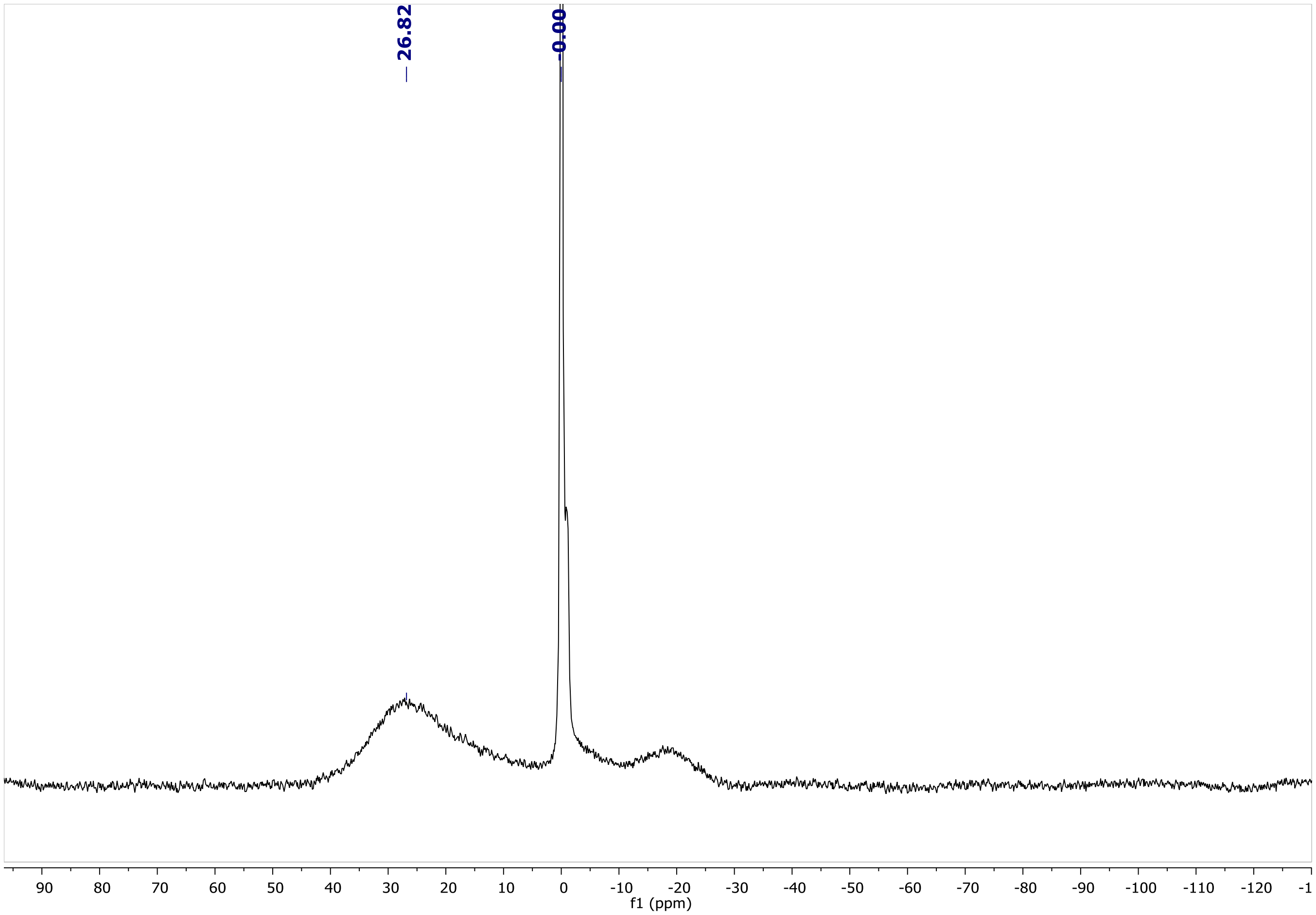

^{11}B NMR, DMSO-d_6

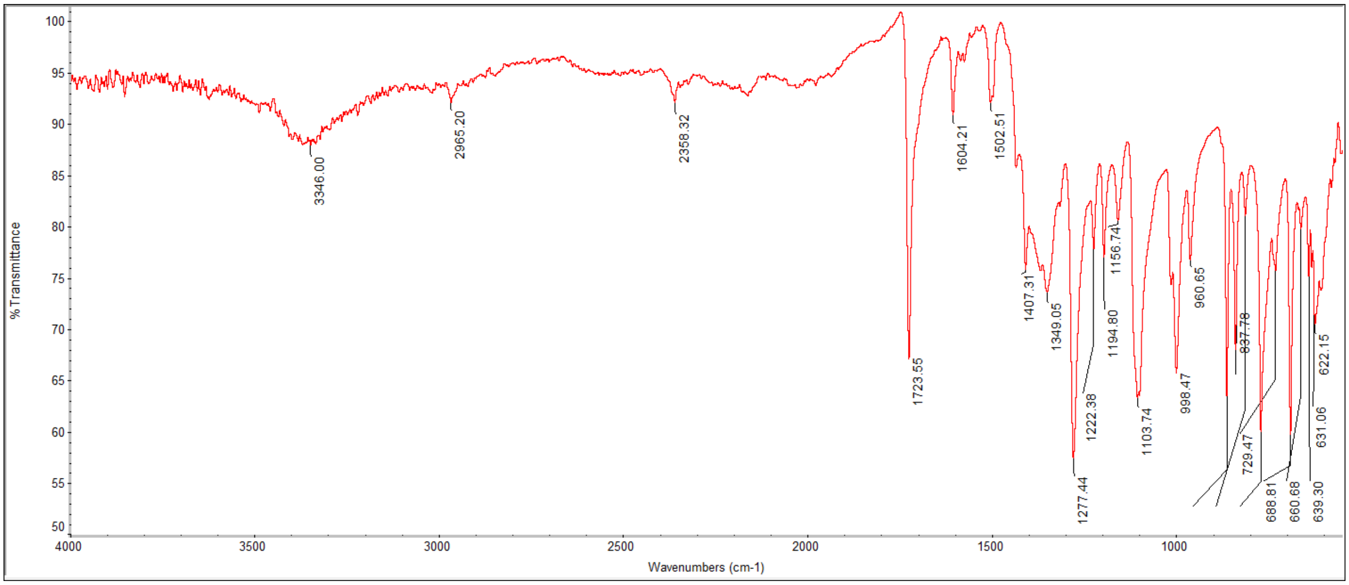

FTIR

SI-**5**

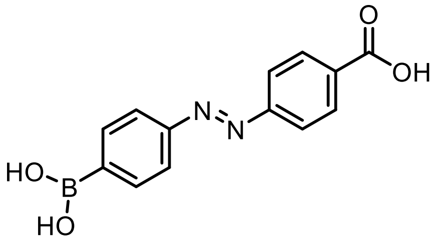

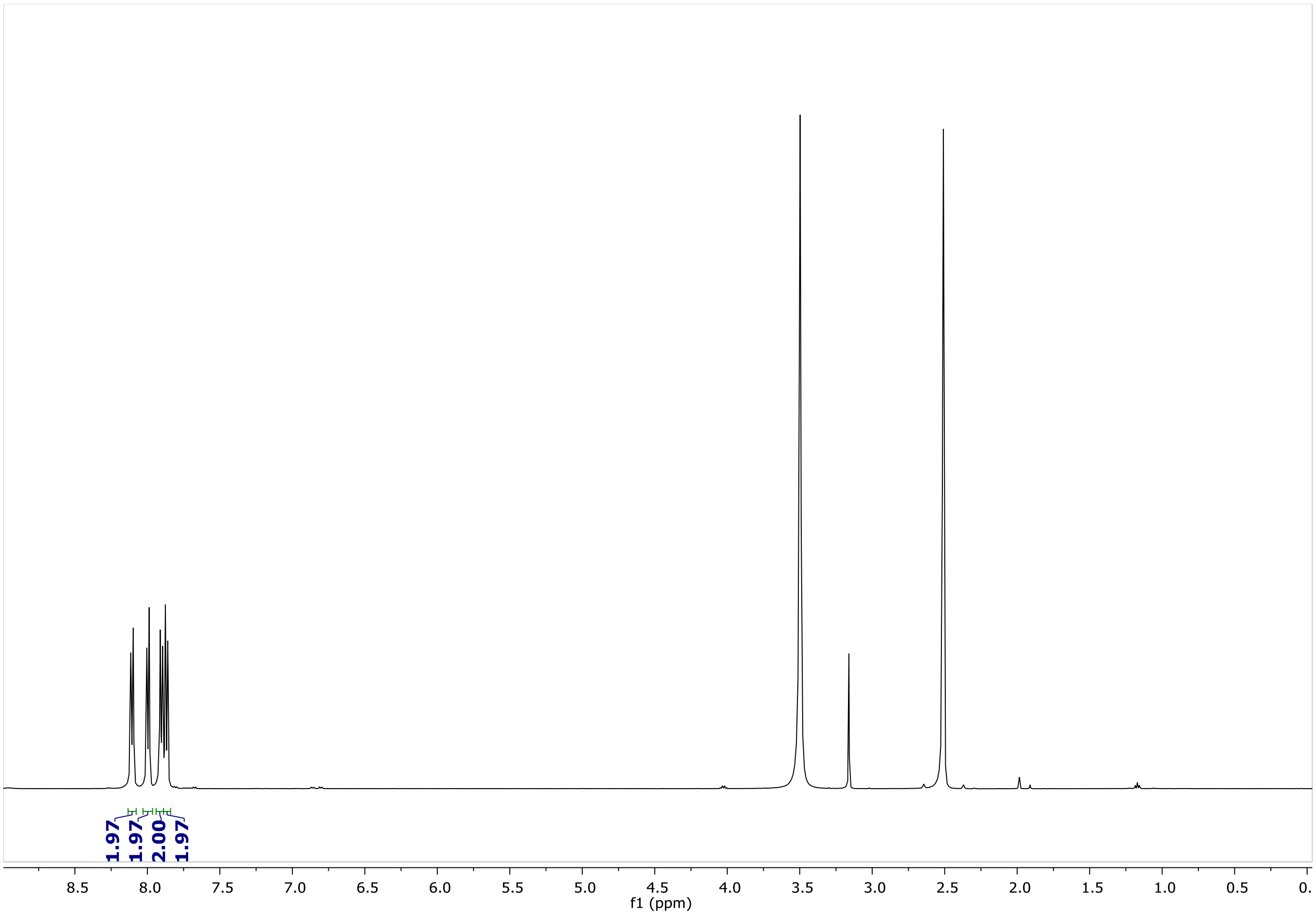

^{1}H NMR, DMSO-d_6

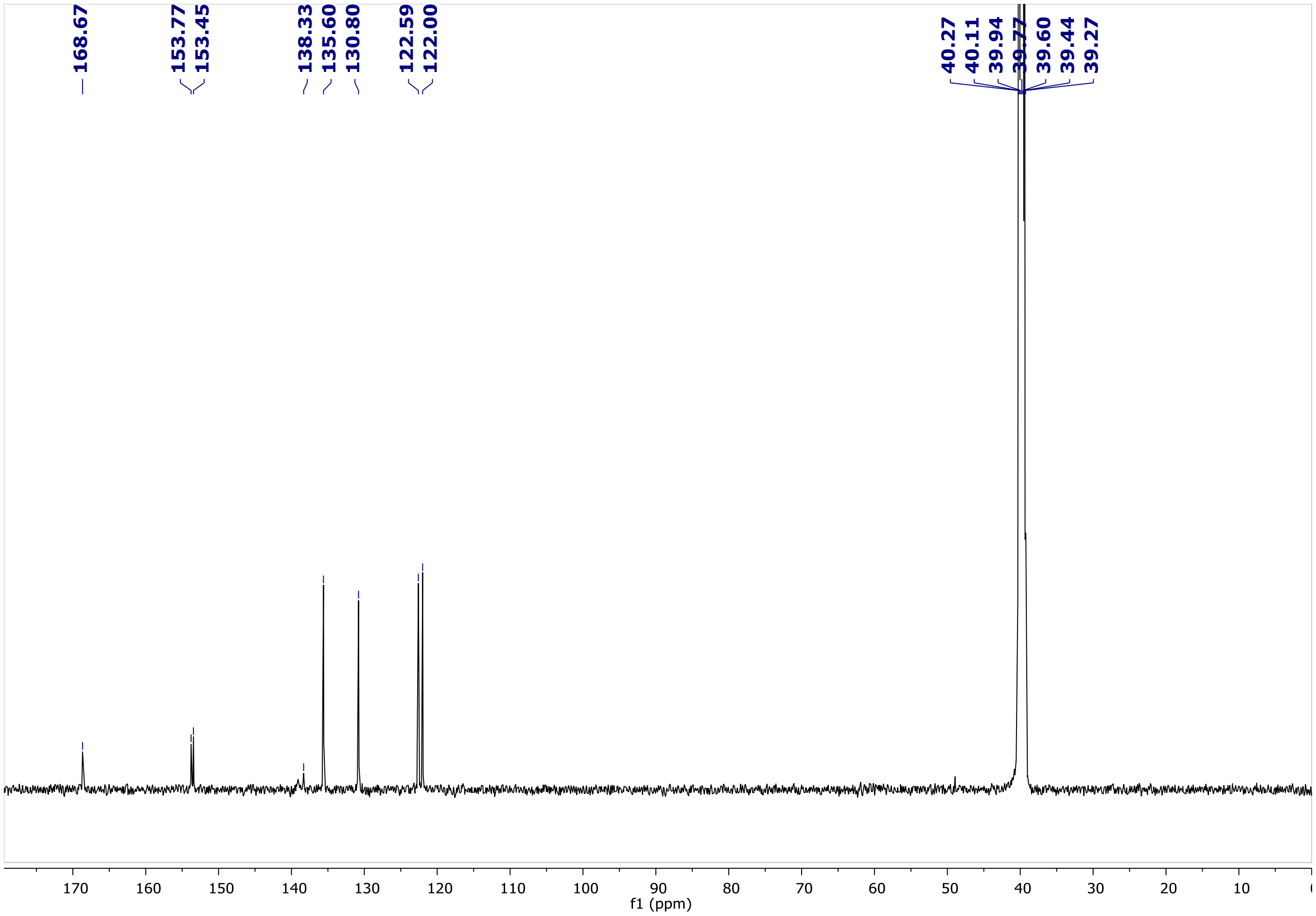

^{13}C NMR, DMSO-d_6

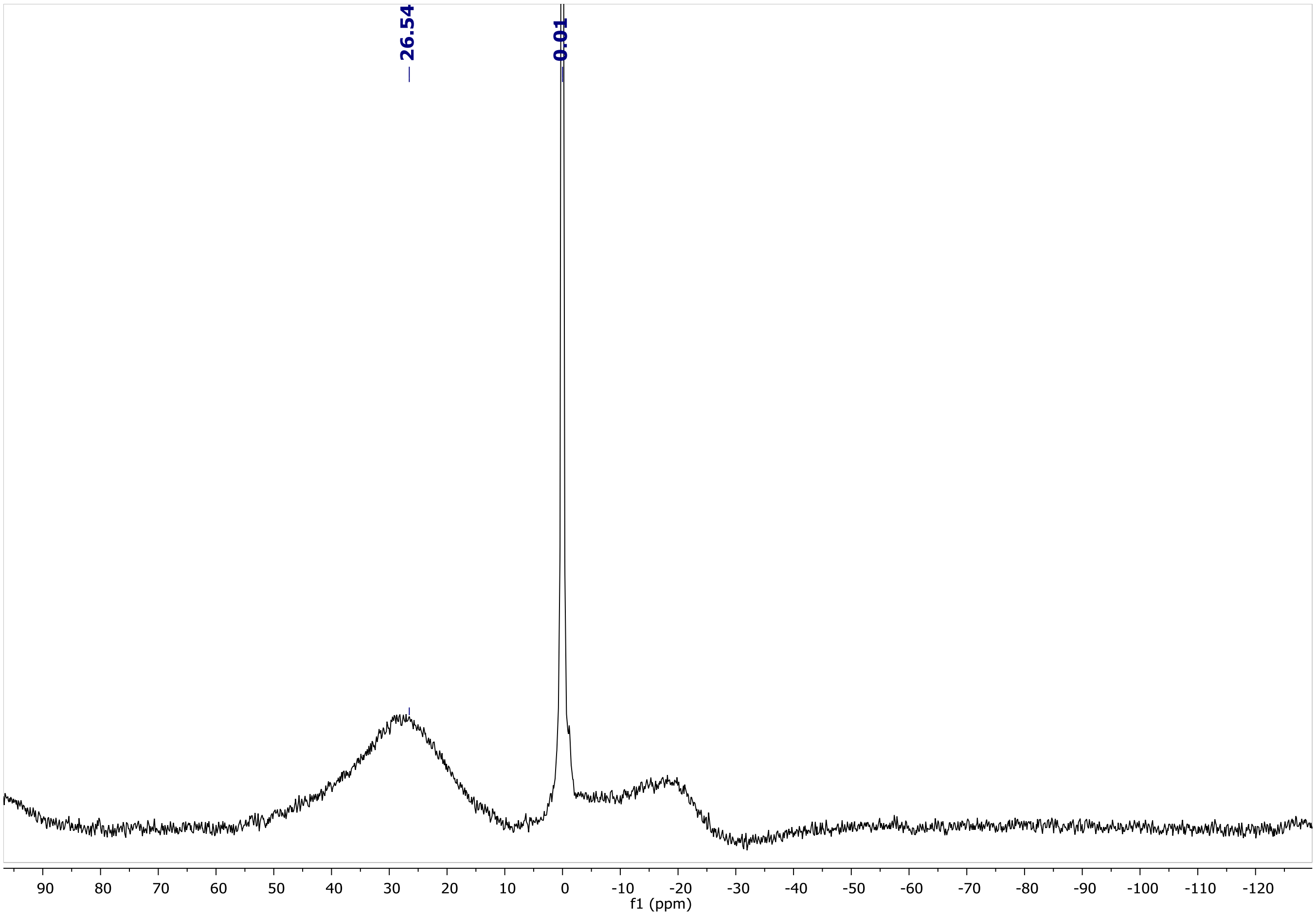

^{11}B NMR, DMSO-d_6

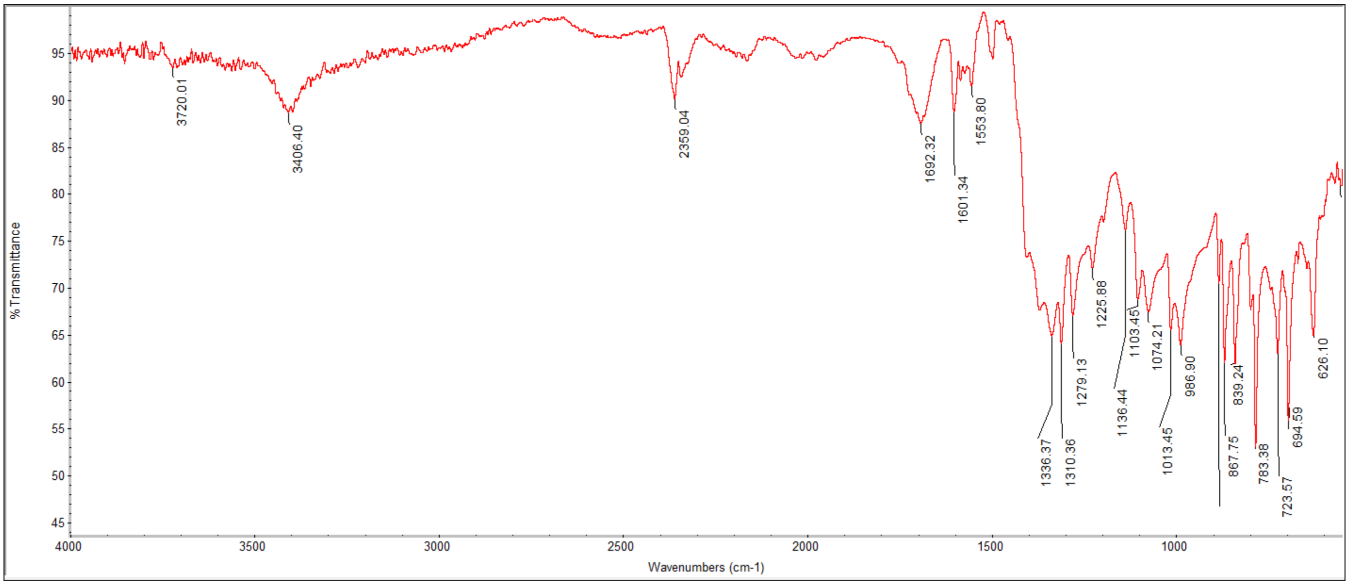

FTIR

SI-**8**

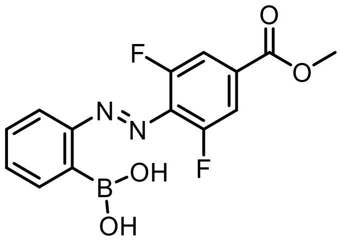

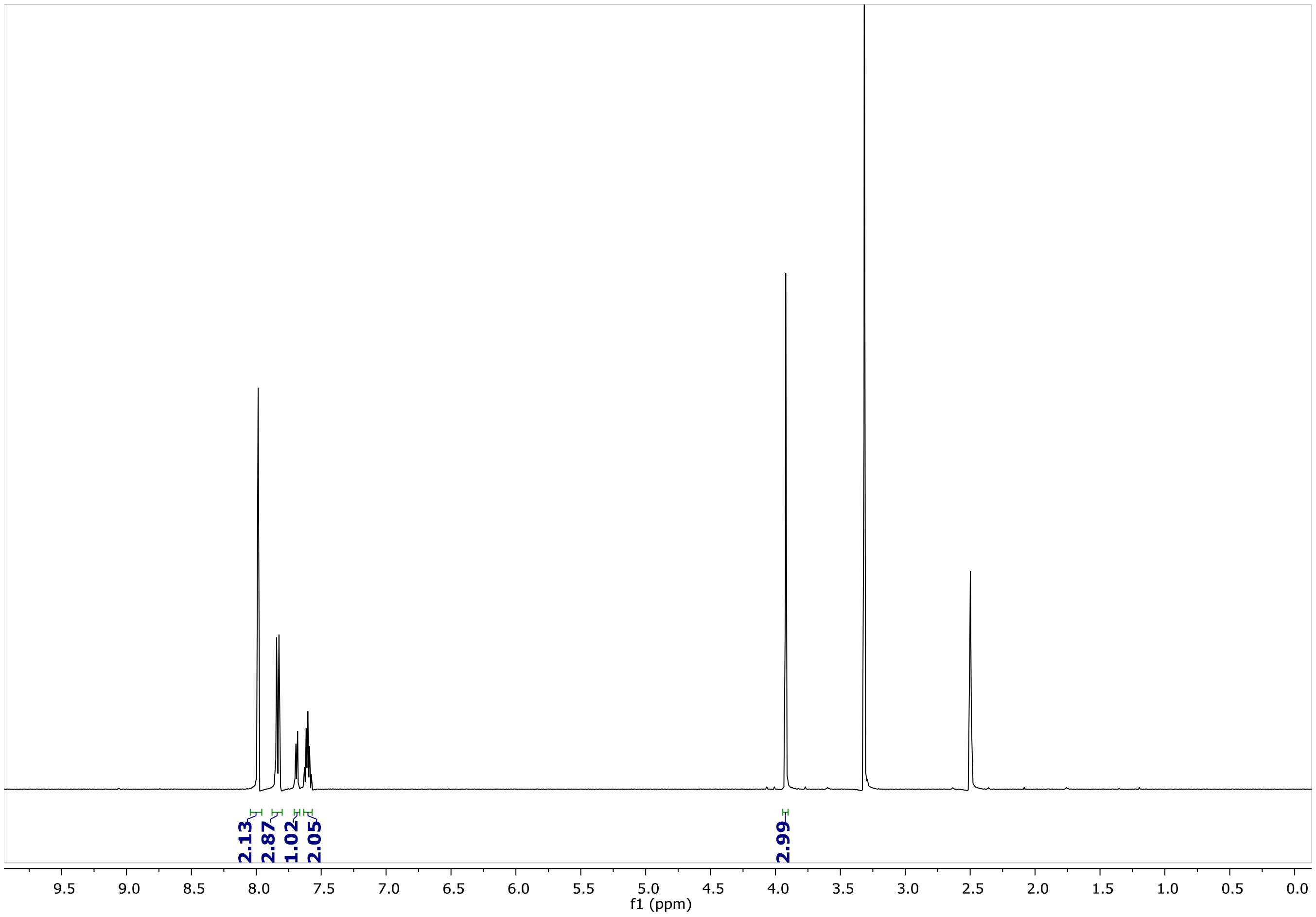

^{1}H NMR, DMSO-d_6

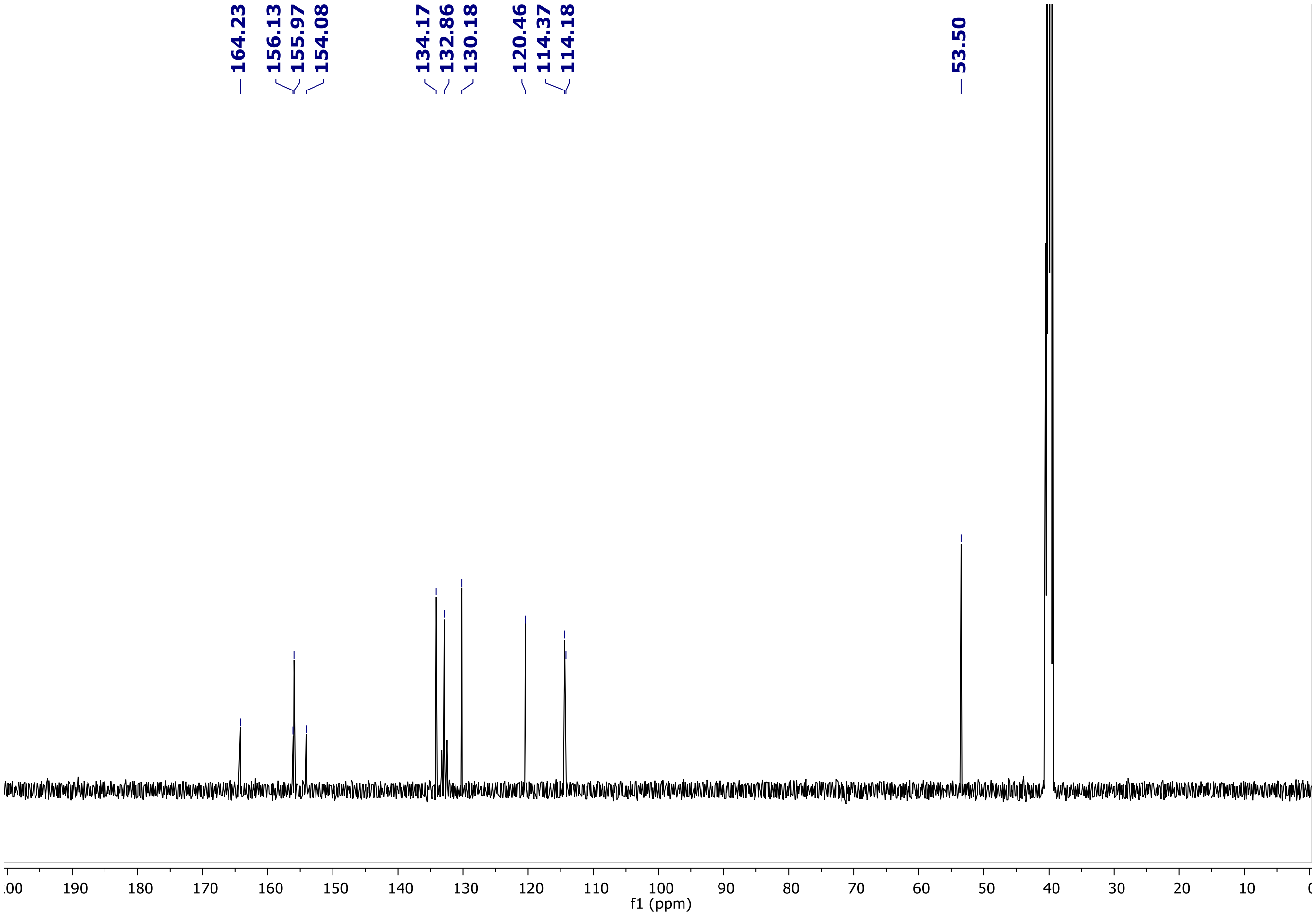

^{13}C NMR, DMSO-d_6

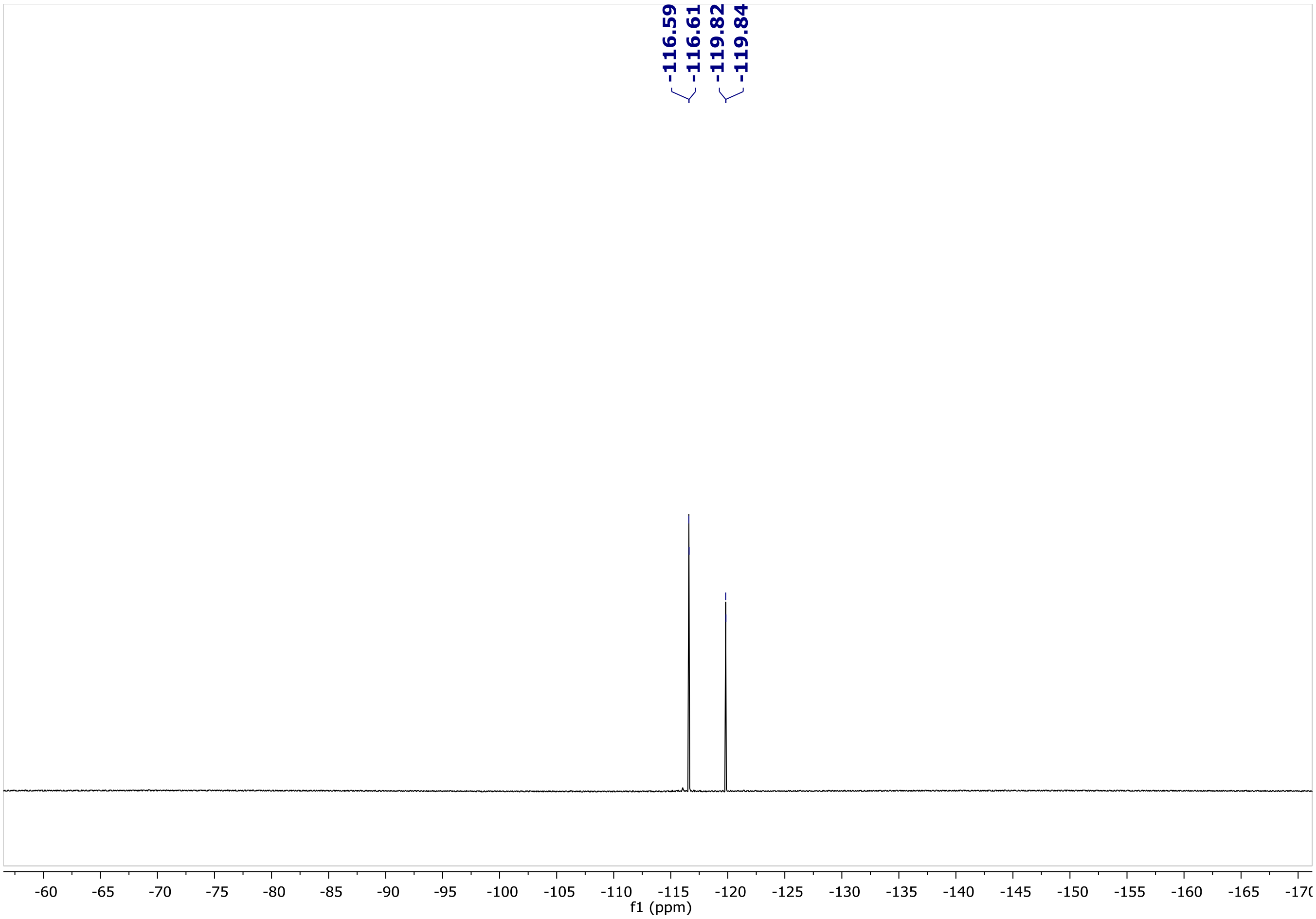

^{19}F NMR, DMSO-d_6

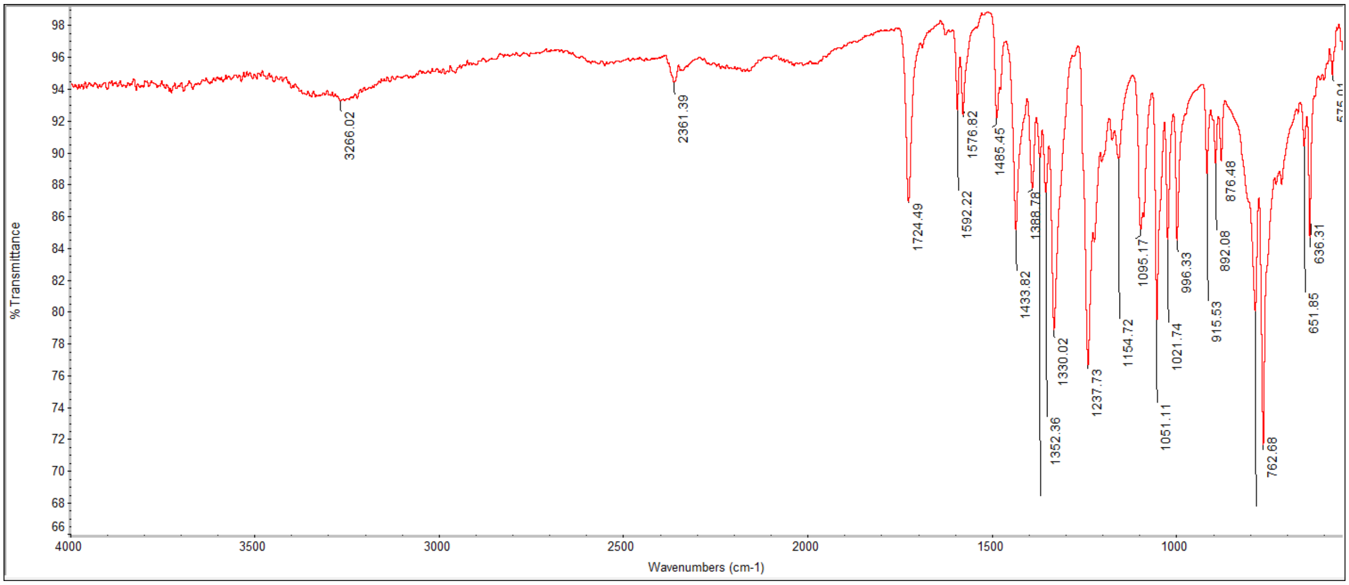

FTIR

SI-**9**

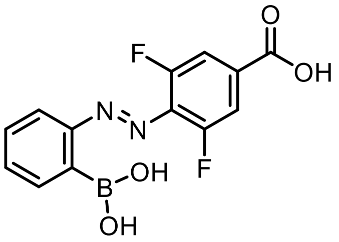

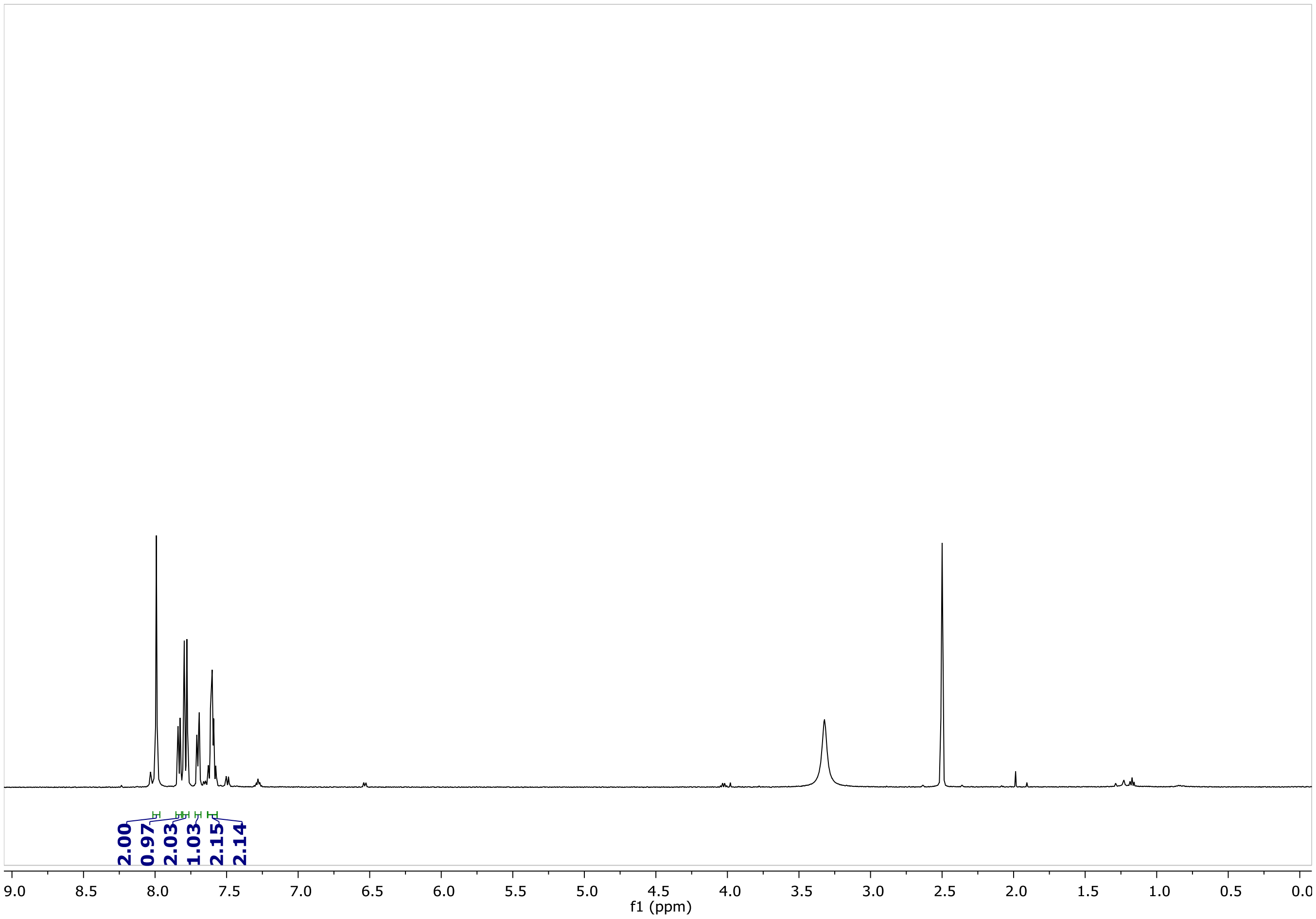

^{1}H NMR, DMSO-d_6

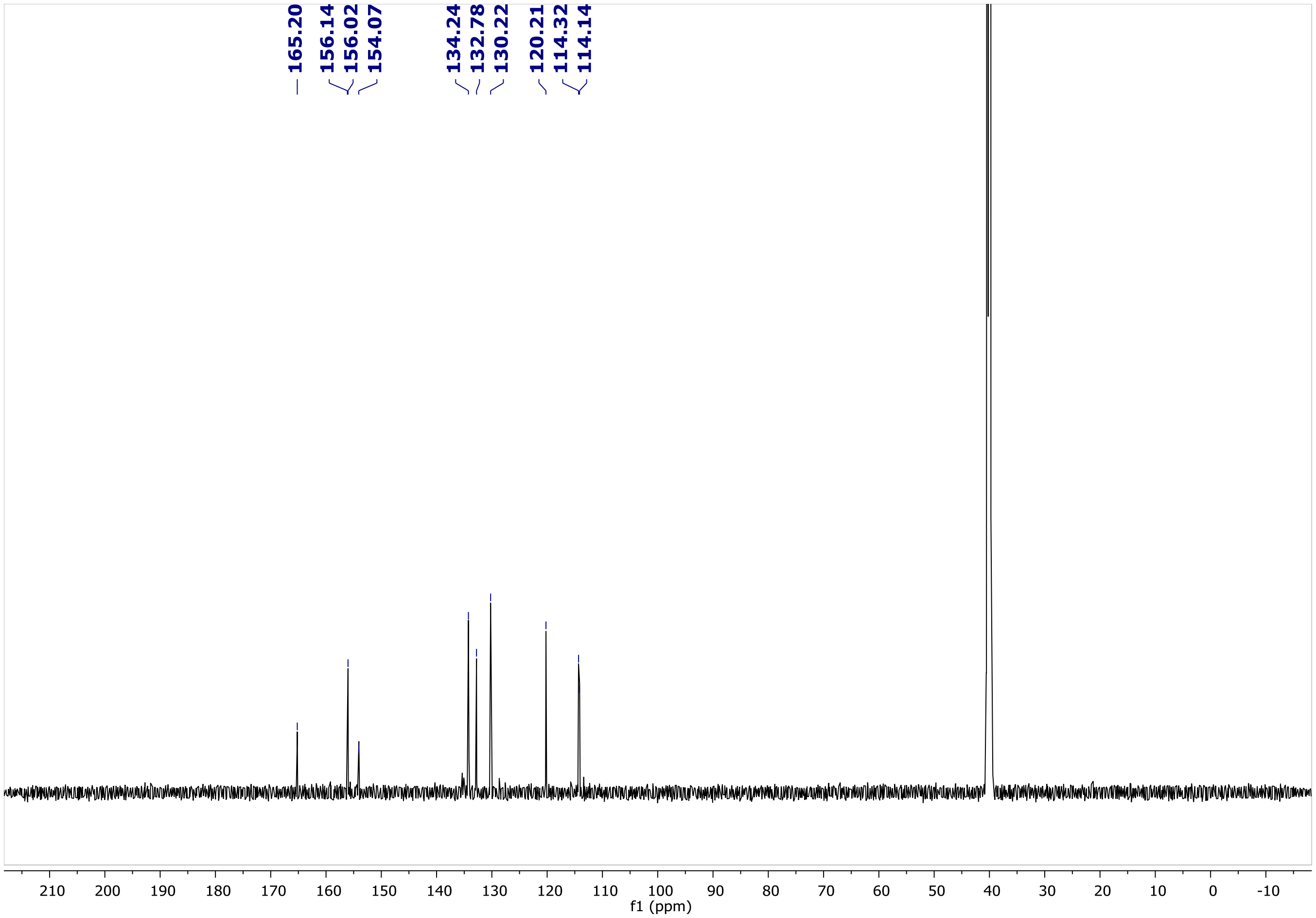

^{13}C NMR, DMSO-d_6

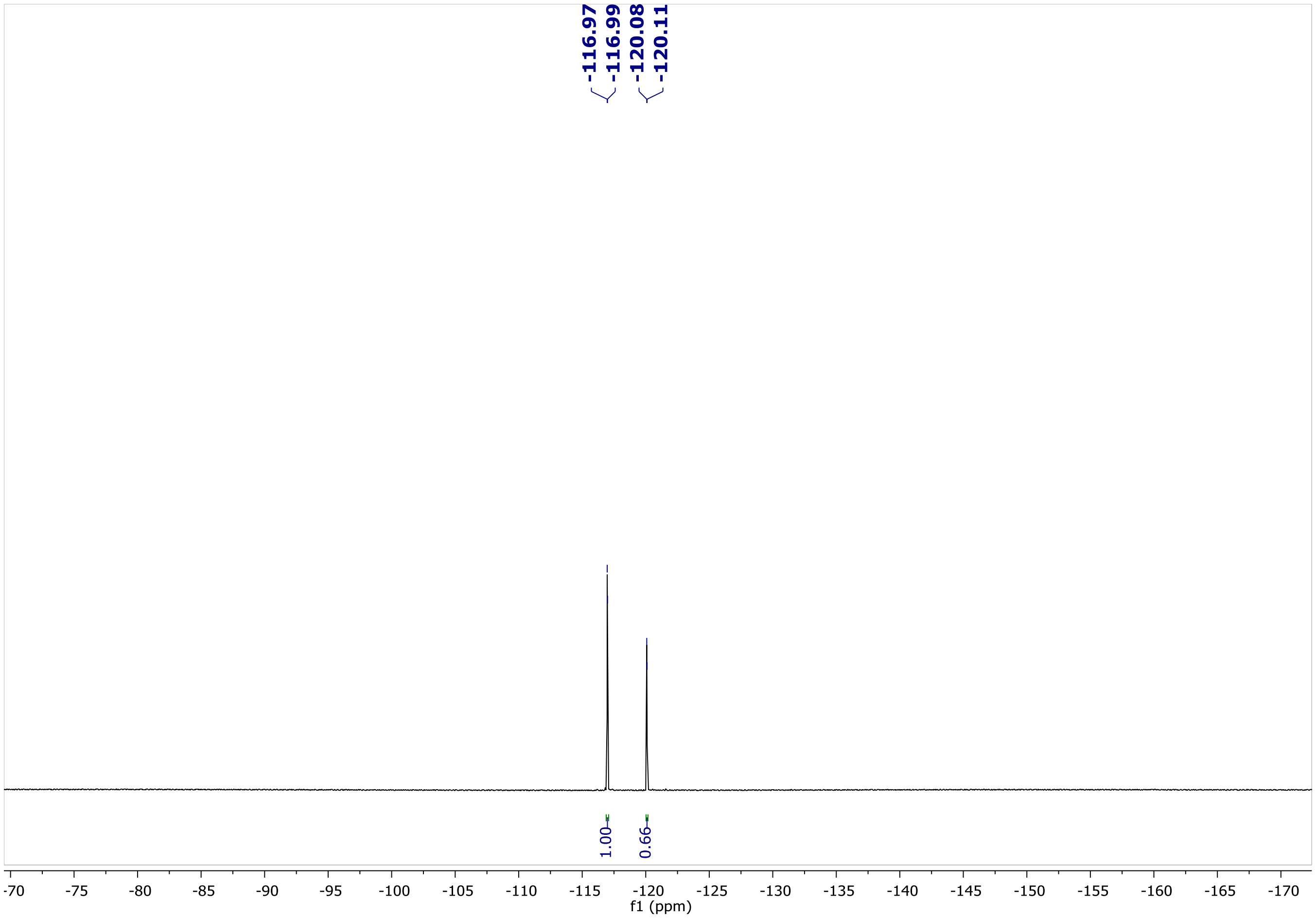

^{19}F NMR, DMSO-d_6

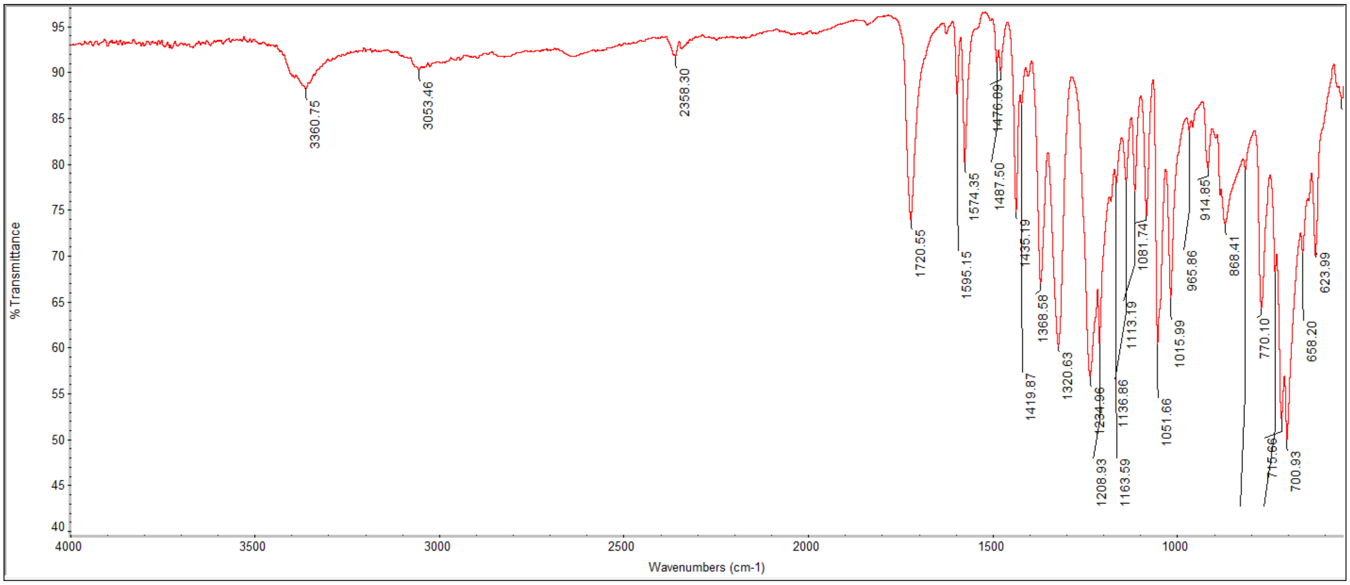

FTIR

P1

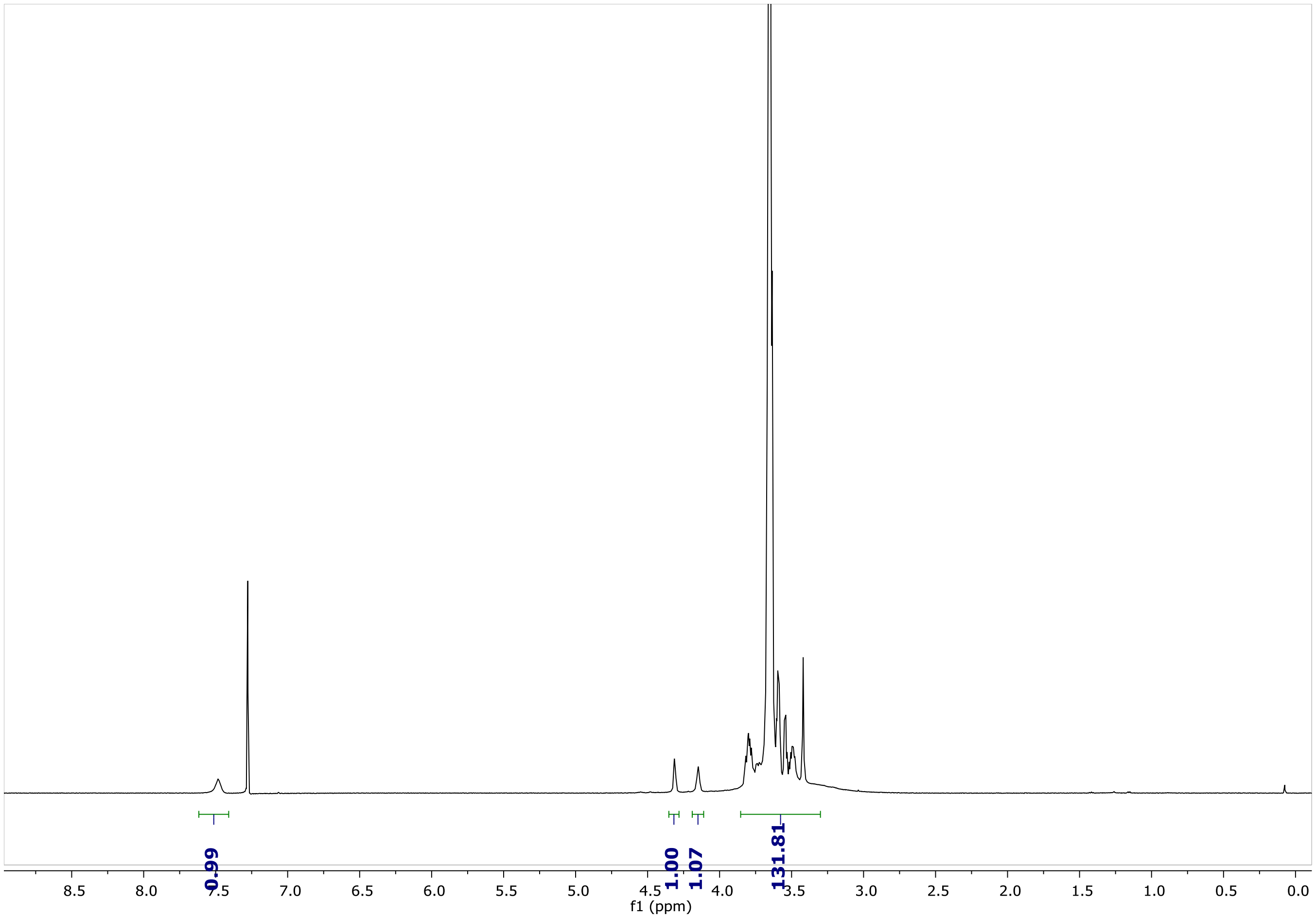

^{1}H NMR, $CDCl_3$

P2

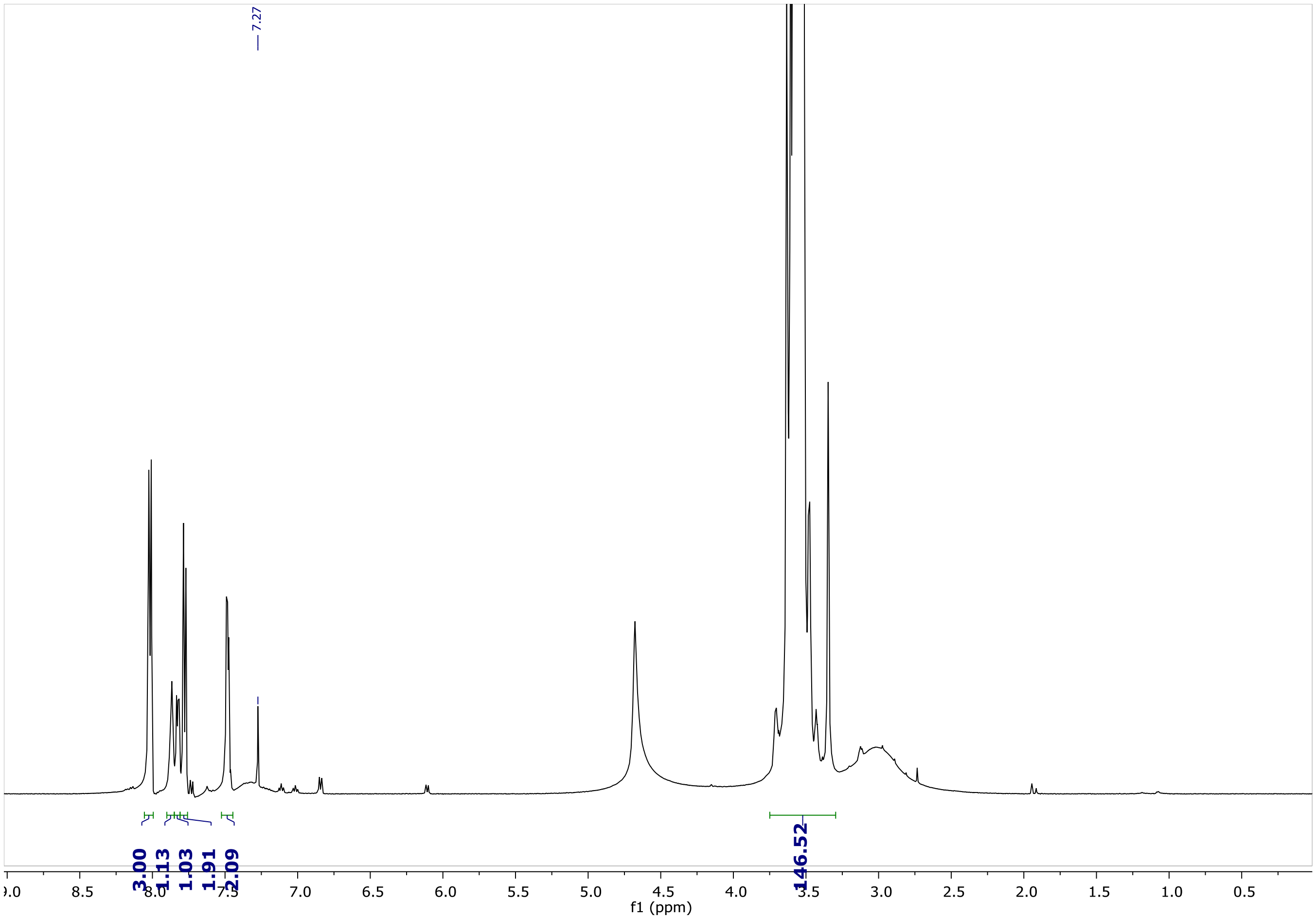

^{1}H NMR, $CDCl_3$

P2-F$_2$

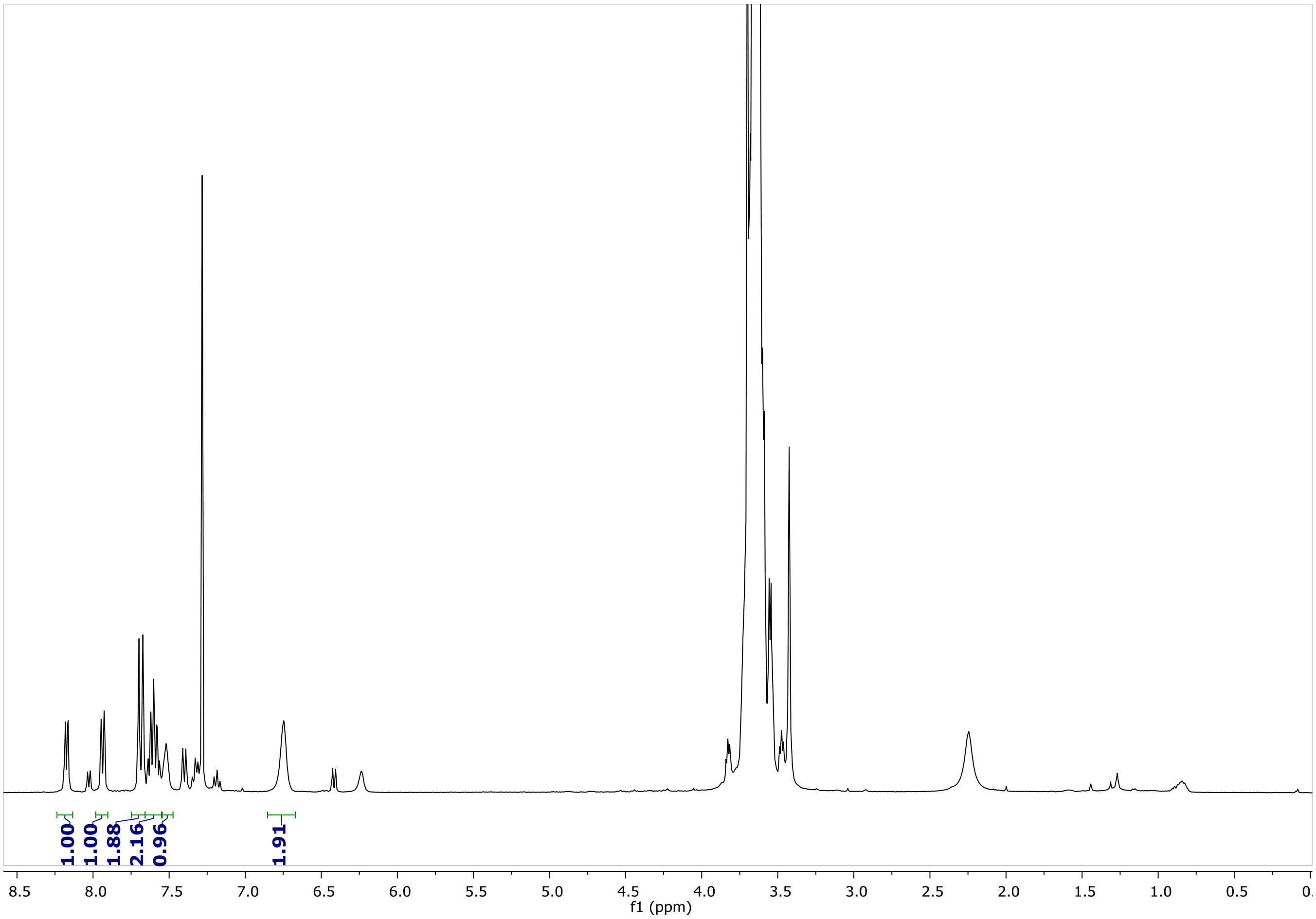

^{1}H NMR, $CDCl_3$

P3

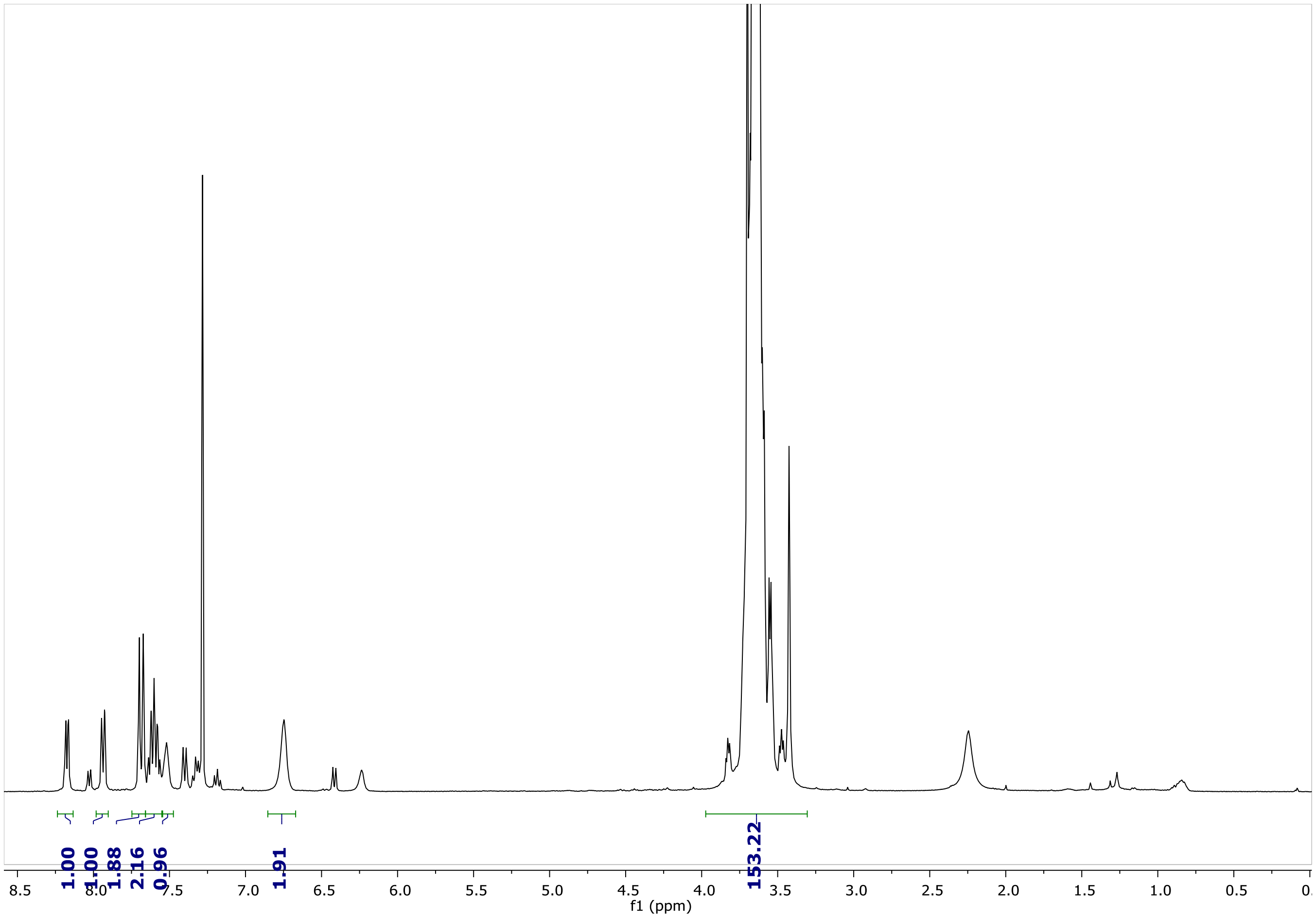

^{1}H NMR, $CDCl_3$

P4

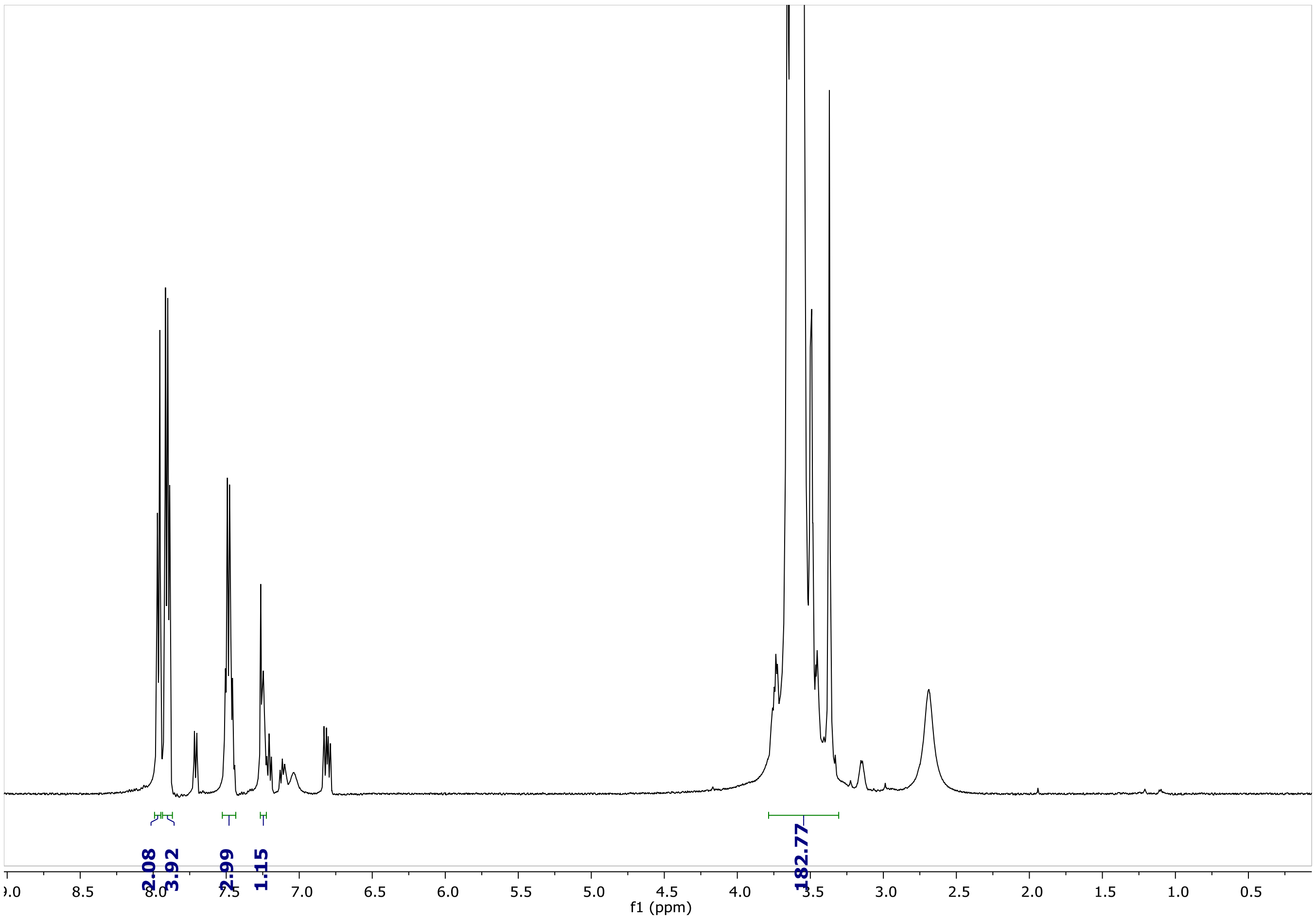

^{1}H NMR, $CDCl_3$

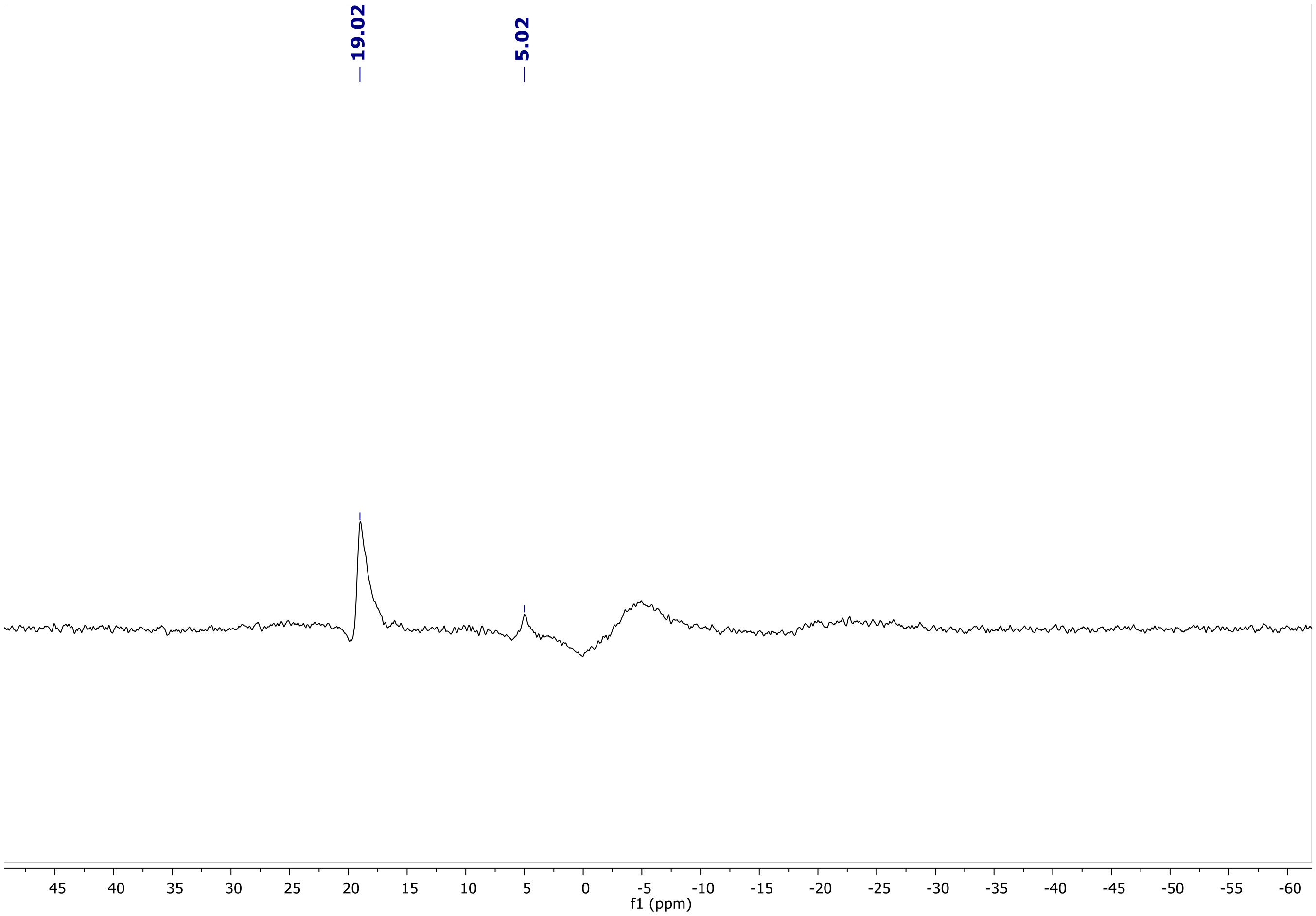

^{11}B NMR of **P2** in DI H_2O after twenty-four hours standing in solution under ambient light. Signals at 19.02 and 5.02 are consistent with the formation of boric acid, a decomposition pathway of **P2**. Protodeboronation has not been quantified, but the reversibility of photorheology experiment suggests the boric acid does not significantly affect gelation.

VIII. Photos

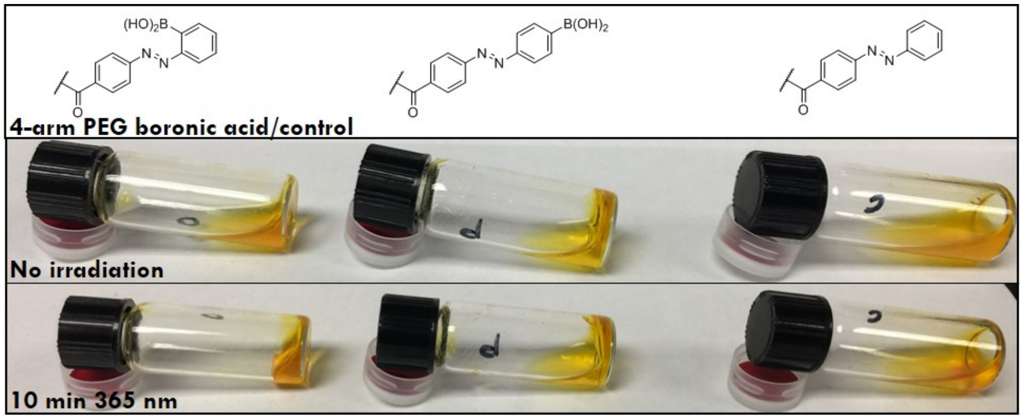

Figure S26. Photo of mixtures of **P1** and **P2** (left), **P1** and **P3** (middle), and **P1** and **P4** (right) (1:1, 10 w/v% in 0.1M PBS, pH 9) before and after ten minutes of 365 nm UV irradiation. A qualitative change in appearance is only seen in the mixture of **P1** and **P2**.

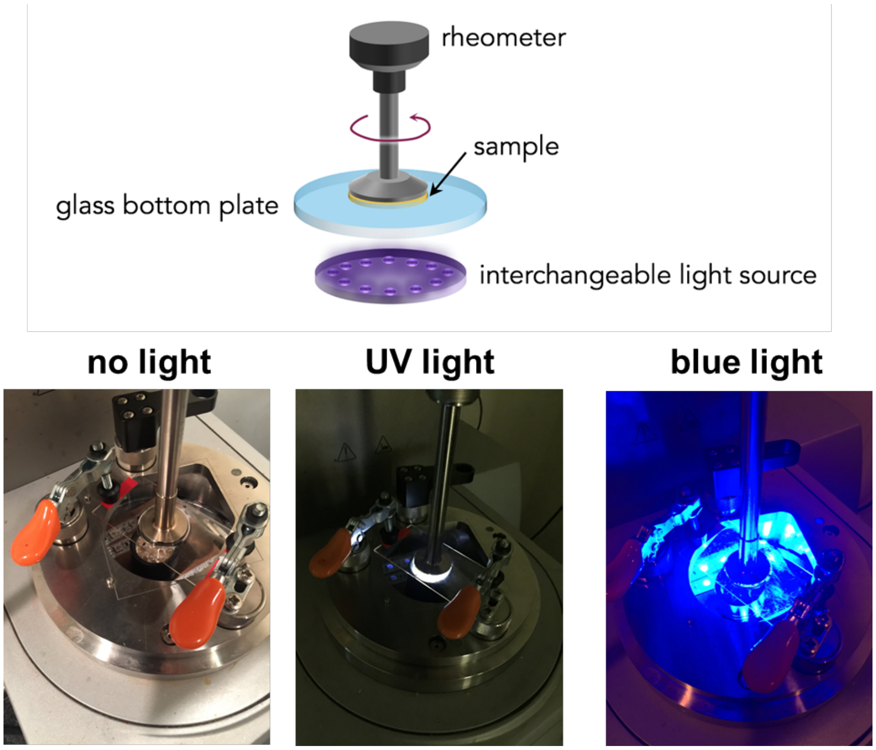

Figure S27. Pictures of the photorheology setup used to characterize photoresponsive hydrogels. The sample sits between the quartz slide and the cone plate. The 365 nm UV flashlight and 470 nm blue LED can be turned on and off and provide irradiation from below the sample. Green LEDs were installed in the same fashion as blue LEDs.

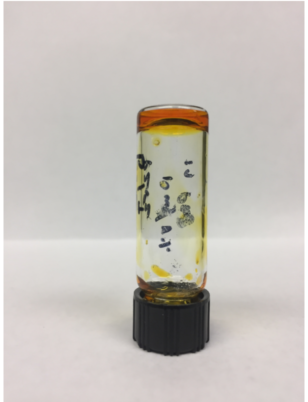

Figure S28. Photo of a gel formed from irradiation of **P1** and **P2**-$\mathbf{F_2}$ (1:1, 10 w/v% in 0.1M PBS, pH 7.5) for one hour with green LED, after standing one week in the dark at 23 °C. As indicated in by the flow inversion method, the network remains a gel.

IX. References

1. Priewisch, B.; Rück-Braun, K., *The Journal of Organic Chemistry.* **2005,** *70*, 2350.
2. Bléger, D.; Schwarz, J.; Brouwer, A. M.; Hecht, S., *J. Am. Chem. Soc.* **2012,** *134*, 20597.
3. Hartley, F. R., *Chem. Br.* **1984,** *20*, 148.
4. Yesilyurt, V.; Webber, M. J.; Appel, E. A.; Godwin, C.; Langer, R.; Anderson, D. G., *Adv. Mater.* **2016,** *28*, 86.
5. Yesilyurt, V.; Ayoob, A. M.; Appel, E. A.; Borenstein, J. T.; Langer, R.; Anderson, D. G., *Adv. Mater.* **2017,** *29*.
